# Long-term survival of children born with congenital anomalies: A systematic review and meta-analysis of population-based studies

**DOI:** 10.1371/journal.pmed.1003356

**Published:** 2020-09-28

**Authors:** Svetlana V. Glinianaia, Joan K. Morris, Kate E. Best, Michele Santoro, Alessio Coi, Annarita Armaroli, Judith Rankin

**Affiliations:** 1 Population Health Sciences Institute, Faculty of Medical Sciences, Newcastle University, Newcastle upon Tyne, United Kingdom; 2 Population Health Research Institute, St George’s, University of London, London, United Kingdom; 3 Institute of Clinical Physiology, National Research Council, Pisa, Italy; 4 Center for Clinical and Epidemiological Research, University of Ferrara, Ferrara, Italy; London School of Hygiene and Tropical Medicine, UNITED KINGDOM

## Abstract

**Background:**

Following a reduction in global child mortality due to communicable diseases, the relative contribution of congenital anomalies to child mortality is increasing. Although infant survival of children born with congenital anomalies has improved for many anomaly types in recent decades, there is less evidence on survival beyond infancy. We aimed to systematically review, summarise, and quantify the existing population-based data on long-term survival of individuals born with specific major congenital anomalies and examine the factors associated with survival.

**Methods and findings:**

Seven electronic databases (Medline, Embase, Scopus, PsycINFO, CINAHL, ProQuest Natural, and Biological Science Collections), reference lists, and citations of the included articles for studies published 1 January 1995 to 30 April 2020 were searched. Screening for eligibility, data extraction, and quality appraisal were performed in duplicate. We included original population-based studies that reported long-term survival (beyond 1 year of life) of children born with a major congenital anomaly with the follow-up starting from birth that were published in the English language as peer-reviewed papers. Studies on congenital heart defects (CHDs) were excluded because of a recent systematic review of population-based studies of CHD survival. Meta-analysis was performed to pool survival estimates, accounting for trends over time. Of 10,888 identified articles, 55 (*n* = 367,801 live births) met the inclusion criteria and were summarised narratively, 41 studies (*n* = 54,676) investigating eight congenital anomaly types (spina bifida [*n* = 7,422], encephalocele [*n* = 1,562], oesophageal atresia [*n* = 6,303], biliary atresia [*n* = 3,877], diaphragmatic hernia [*n* = 6,176], gastroschisis [*n* = 4,845], Down syndrome by presence of CHD [*n* = 22,317], and trisomy 18 [*n* = 2,174]) were included in the meta-analysis. These studies covered birth years from 1970 to 2015. Survival for children with spina bifida, oesophageal atresia, biliary atresia, diaphragmatic hernia, gastroschisis, and Down syndrome with an associated CHD has significantly improved over time, with the pooled odds ratios (ORs) of surviving per 10-year increase in birth year being OR = 1.34 (95% confidence interval [95% CI] 1.24–1.46), OR = 1.50 (95% CI 1.38–1.62), OR = 1.62 (95% CI 1.28–2.05), OR = 1.57 (95% CI 1.37–1.81), OR = 1.24 (95% CI 1.02–1.5), and OR = 1.99 (95% CI 1.67–2.37), respectively (*p* < 0.001 for all, except for gastroschisis [*p* = 0.029]). There was no observed improvement for children with encephalocele (OR = 0.98, 95% CI 0.95–1.01, *p* = 0.19) and children with biliary atresia surviving with native liver (OR = 0.96, 95% CI 0.88–1.03, *p* = 0.26). The presence of additional structural anomalies, low birth weight, and earlier year of birth were the most commonly reported predictors of reduced survival for any congenital anomaly type. The main limitation of the meta-analysis was the small number of studies and the small size of the cohorts, which limited the predictive capabilities of the models resulting in wide confidence intervals.

**Conclusions:**

This systematic review and meta-analysis summarises estimates of long-term survival associated with major congenital anomalies. We report a significant improvement in survival of children with specific congenital anomalies over the last few decades and predict survival estimates up to 20 years of age for those born in 2020. This information is important for the planning and delivery of specialised medical, social, and education services and for counselling affected families. This trial was registered on the PROSPERO database (CRD42017074675).

## Abstract 2

In a systematic review and meta-analysis, Svetlana V. Glinianaia and colleagues report on temporal trends in long-term survival of children born with specific congenital anomalies.

## Author summary

**Why was this study done?:**

- Following a reduction in global child mortality due to communicable diseases, the relative contribution of congenital anomalies to child mortality under age 5 years is increasing globally.
- Identifying and addressing the emerging priority of congenital anomalies, including for children aged 5–9 years, is one of the strategic directions for the post-2015 child health agenda.
- This research aimed to summarise and quantify the existing population-based evidence on long-term survival of children born with specific major congenital anomalies that manifest in childhood.

**What did the researchers do and find?:**

- This systematic review included 55 international studies that estimated survival beyond 1 year of age of children born with major congenital anomalies.
- Our meta-analysis results of 41 studies over the birth years 1970–2015 showed a statistically significant improvement in survival over time in children with spina bifida, oesophageal atresia, biliary atresia, congenital diaphragmatic hernia, gastroschisis, and Down syndrome associated with a congenital heart defect, but there was no evidence of improvement in those with encephalocele or biliary atresia with a native liver.
- The commonest significant independent predictors of reduced survival for any congenital anomaly type were presence of additional structural anomalies, low birth weight, and earlier birth year period.

**What do these findings mean?:**

- A significant improvement in survival of children with specific congenital anomalies over the last few decades reported by individual studies and identified by the meta-analysis has important public health, medical, social, and family implications.
- Information on predicted survival of children with congenital anomalies up to 20 years of age is important for planning specialised medical, social, and education services for these children and for estimating costs associated with special care needs in childhood and adulthood.

## Introduction

Globally, mortality in children aged under 5 years has halved since 1990, mainly because of a sharp reduction in deaths from communicable diseases as a result of targeted child health strategies and interventions of the United Nations (UN) Millennium Development Goals [1]. Following this worldwide reduction, the relative contribution of congenital anomalies to child mortality is increasing globally and is therefore outlined as an emerging priority to be addressed by the UN Sustainable Development Goals in the post-2015 child health agenda [2]. Although the contribution of congenital anomalies to infant mortality is well described, in particular for developed countries [3–5], there is less research focused on survival beyond the first year of life. However, this is of considerable public health importance, as according to evidence from North America and Europe, the mortality rate of individuals born with congenital anomalies significantly exceeds that of the general population after infancy as well [6–9]. In addition, a large variation in child death rates still exists between countries, even within Europe [10]. In 2012, the child death rates (age 0–14 years) were about 60% higher in the United Kingdom and Belgium compared to Sweden, with an additional 10 Western European countries being 30% higher than Sweden [10]. Currently, a quantitative summary of population-based studies of survival beyond infancy for specific congenital anomalies is lacking. Accurate estimates of long-term survival are important for clinicians counselling parents when a congenital anomaly is diagnosed pre- or postnatally and for public health commissioners to ensure adequate resources are in place to provide high-quality medical and social care for these individuals. Importantly, it is essential that estimates are provided according to type of congenital anomaly, given the diversity in aetiology, treatment, and prognosis.

We performed a systematic review and meta-analysis to summarise and quantify the existing population-based data on long-term survival (beyond infancy) of individuals born with specific major congenital anomalies that manifest in childhood and explore the risk factors associated with survival.

## Methods

### Search strategy

This study is reported as per the Preferred Reporting Items for Systematic Reviews and Meta-Analyses (PRISMA) guideline (S1 PRISMA Checklist). A protocol for this systematic review was registered on the International Prospective Register of Systematic Reviews (PROSPERO) database (CRD42017074675) (S1 Text). We conducted comprehensive literature searches using a combination of the following sources of information:

1. Electronic bibliographical databases: MEDLINE, EMBASE, Scopus, PsycINFO, CINAHL, ProQuest Natural, and Biological Science Collections and also the databases of the systematic reviews, i.e., PROSPERO, the JBI Database of Systematic Reviews and Implementation Reports. We used key words and subject headings (dependent on the database) combining the keywords for the population (birth, pregnancy, delivery), exposure (congenital anomaly, including specific anomaly groups), outcome (long-term survival, mortality), and study design (population-based studies), incorporating elements of the PICOS (Population/Patient, Intervention/Exposure, Comparator group, Outcome, Study design) framework into our systematic search strategy [11] (S1 Table). The final search results were limited to English papers and to humans, whereas the initial searches had no language limitations to examine whether there were any relevant studies we could have missed. We have identified 66 papers published in non-English language (79% from Europe) based on Medline search, but no papers met our inclusion criteria.
2. Manual searching of the reference lists of the included full papers and of the relevant previous literature reviews, including systematic, was performed.
3. Citation searching for studies that had referenced the included studies was performed via the Google Scholar citation function.
4. Keyword searches in key journals, including *Birth Defects Research*, *Archives of Disease in Childhood*, *Pediatrics*, *The Journal of Pediatrics*, and *Journal of Pediatric Surgery*, were also undertaken.
5. Authors were contacted if there was insufficient information to decide whether the study met the inclusion criteria or if additional information for the inclusion in the meta-analysis was needed.
6. Reference lists and citations of any new articles identified were further searched for any additional studies in the iterative process until no new studies were identified. Database searches were completed in March 2019 and updated in May 2020.

SVG conducted all searches and screened the titles and abstracts of all the identified records according to the inclusion criteria, and three other authors (MS, AC, JR) independently screened a random 10% sample of the records using the Rayyan software for systematic reviews [12]. Any discrepancies (*n* = 4) in the included studies were discussed amongst all authors and agreement reached.

### Definitions and classification of congenital anomalies

Major congenital anomalies in the included studies were classified according to the International Classification of Disease (ICD) revision 8 (ICD-8) [8], ICD-9 (majority of papers), ICD-10 [13–15], and British Paediatric Association (BPA-ICD-9) diagnosis coding [16–20] or surgical codes [21]. Some papers that included a long birth year period used more than one ICD version for the corresponding time periods [9,22–25]. The included studies reported the survival estimates for all congenital anomalies combined (e.g., ICD-9 codes 740.0–759.9) and/or by congenital anomaly group (the system affected, e.g., urinary system, ICD-9 753.0–753.9) and/or subtype (the individual disorder, e.g., spina bifida, ICD-9 741). Some European studies [14,15,17] classified major congenital anomalies according to European Surveillance of Congenital Anomalies (EUROCAT) guidelines [26,27]. We have presented the congenital anomaly subtypes within the major congenital anomaly groups according to the EUROCAT classification [26].

### Eligibility criteria

Studies meeting the following criteria were included: (1) being an original population-based peer-reviewed study that reported long-term (beyond 1 year of life) survival of children born with a major congenital anomaly that manifests in childhood; (2) reporting survival probability (or the number of patients born and the number or proportion alive at age >1 year) for these children that were followed up from birth; (3) being published from 1 January 1995 to 30 April 2020 to increase comparability of included birth cohorts in relation to medical care and treatment availability/policies; (4) involving humans only and published in the English language.

Studies were excluded if (1) they reported survival during the first year of life only; (2) patients were not followed up from birth, because this may have under-ascertained deaths occurring prior to follow-up (e.g., if follow-up began after surgical correction); (3) they were not population-based, as other study designs are more likely to incur ascertainment bias (e.g., hospital-based studies may capture more severe phenotypes); (4) they focused on individuals born with congenital heart defect (CHD), because there was a recently published systematic review covering these population-based studies [28]; (5) they followed up a restricted subgroup of patients (e.g., preterm births only or extracorporeal membrane oxygenation [ECMO] patients only). No exclusions were made based on the birth year of studied cohorts.

### Data extraction

Information on the following study characteristics was extracted: study location, birth year period, duration of follow-up/years of survival, congenital anomaly type and if isolated/non-isolated, sources of case ascertainment (e.g., congenital anomaly register) and sources of death identification (e.g., linkage with a mortality database), number of cases and deaths, Kaplan-Meier survival estimates reported, or the survival estimates calculated by the reviewers. Authors were contacted if survival estimates were reported for subgroups of patients only (e.g., by sex or age at operation), if it was not possible to calculate 95% confidence intervals (95% CIs) or extract survival estimates from the Kaplan-Meier curves, or if further information was required or clarification needed (*n* = 18). If the authors did not respond after two reminders or if the study was closed and access to the data was not possible, we calculated the lower and upper limits of the 95% CI according to the efficient-score method (corrected for continuity) described by Newcombe, 1998 [29], based on the procedure outlined by Wilson, 1927 [30] (http://www.vassarstats.net/survival.html). If survival estimates were not reported in the text or tables of the included paper, they were extracted from Kaplan-Meier survival curves, where available, using PlotDigitizer software [31]. If none of the above was possible, the study was excluded.

Data extraction and quality appraisal of the included studies were performed in duplicate, i.e., all by SVG and a subset of studies by each coauthor. Data were entered into piloted data extraction forms (S2 Table).

### Statistical analysis

Where three or more articles reported survival with the number of births (or where the numbers of births could be estimated from the 95% CIs provided) for a specific congenital anomaly, a meta-analysis was performed to estimate pooled survival at ages 1, 5, 10, and 20 (and 25, where available) years. The Stata program “gllamm” was used to fit univariate multilevel meta-analysis of longitudinal data to allow for the correlations in survival over several time periods within studies [32,33]. Survival according to age (0–25 years) was modelled using the logistic regression options within the gllamm program: family(binomial) and link(logit). The outcome of interest was the number of deaths occurring out of the total number of live births. The number of deaths at each time point, if not provided, was estimated from the published proportions surviving and the number of live births by assuming there was no loss to follow-up. Calculating the number of deaths in this way will be unbiased (as the proportion surviving is unbiased) but will result in slightly too narrow confidence intervals. To confirm that this is valid, an alternative method using the arcsine square root transformation [34] of the published survival estimate was applied and the estimated standard error was calculated, and a model was fitted in gllamm using the weighted regression options instead of the logistic regression above. Both methods reported consistent results, and hence, the results of the logistic regression models are reported here, as they enable the interpretation of the odds of increasing survival over time. Studies were treated as a random effect and cohort of birth and age at survival as fixed effects nested within the studies. Age was modelled as a continuous variable using a linear term or, where significant (according to a likelihood ratio test), a quadratic term. Cohort of birth was modelled as a continuous variable. Most included studies reported survival across distinct periods (e.g., between 2000 and 2009), so the mean year of birth was used (e.g., 2005). For studies that reported survival estimates for multiple cohorts (e.g., 2000–2004, 2005–2009), survival for both cohorts were entered into the model, again with average year of birth for each cohort (e.g., 2002 and 2007). Using the models, survival at ages 1, 5, 10, 20, and 25 years was estimated for patients born in 2000 and 2020. Models were fitted separately for each type of congenital anomaly. Odds ratios (ORs) representing the increase/decrease in survival per 10-year increase in time were extracted from the models. Where fewer than three studies reported survival for a specific congenital anomaly, the survival estimates were discussed narratively. The ages for which more than three studies reported a survival rate were plotted separately; often, the reports were at 5 or 10 years of age. This allows the reader to evaluate the changes that have occurred over time in the survival of the children up to 5 years of age and separately up to 10 years of age. All modelled survival curves, although plotted on two separate figures, are derived from the one model fitted on all the data.

Analysis was performed in Stata 15 (StataCorp), and *p* < 0.05 was considered statistically significant.

### Quality appraisal

The Newcastle-Ottawa Quality Assessment Scale (NOS) for cohort studies [35] was used to assess the quality of the included studies. The scale assesses information bias, selection bias, and confounding (S2 Table). Although a traditional cohort study can be awarded a maximum of nine stars, for survival population-based studies a comparison group is not a mandatory component of the study design; therefore, a maximum of six stars can be allocated to the majority of the included studies (S3 Table).

## Results

### Search results

**Fig 1:**
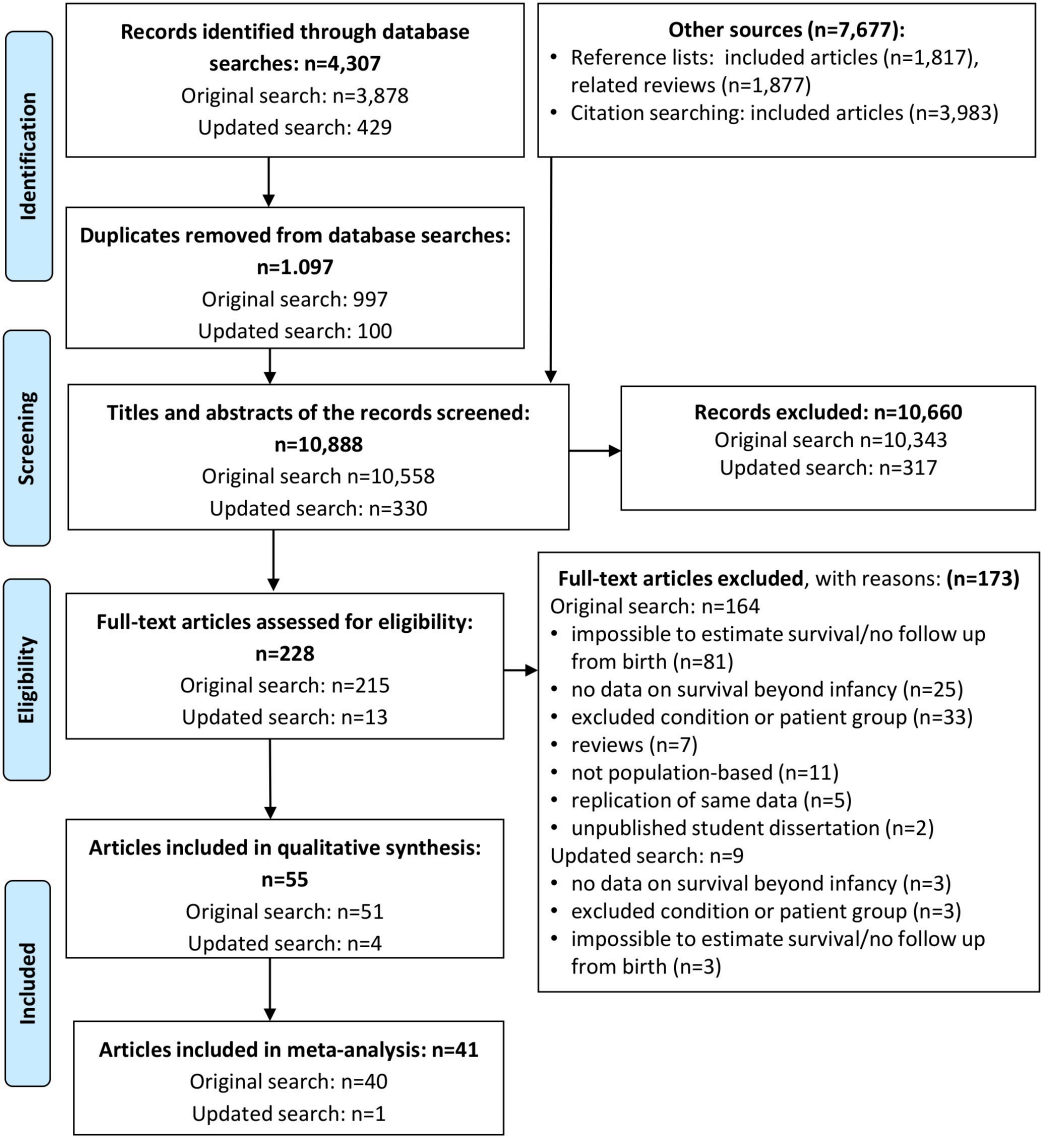
PRISMA flowchart of searches, screening, and study selection. PRISMA, Preferred Reporting Items for Systematic Reviews and Meta-Analyses.

A total of 10,888 records identified from the electronic database searches and other sources were available for screening titles and abstracts (Fig 1). After excluding 10,660 records, 228 were eligible for full text review. After further exclusion of 173 articles, 55 met the inclusion criteria, covering a total population of 367,801 live births with various types of major congenital anomalies. Earlier follow-up studies based on the same population were replaced by more recent ones if they also reported survival at a younger age (*n* = 2 [36,37]). However, if survival at a more advanced age only was reported in the later article [38], the earlier article was also included (*n* = 1 [39]).

### Characteristics of included studies

Table 1 provides the description of 55 studies included in this review. Further detail on the sources of case ascertainment and death identification and the description of the comparison group, if any, are given in S4 Table. Nine studies analysed long-term survival of all congenital anomalies combined: seven with [6,8,15,17,40–42] and two without [7,43] stratification by congenital anomaly group/subtype (Table 1). Other studies (*n* = 46) focused on specific groups or subtypes of congenital anomalies: the central nervous system (*n* = 5 [44–49]), including spina bifida [44–46,48,49] and encephaloсele [44,47]; orofacial clefts (*n* = 1 [16]); anomalies of the digestive system (*n* = 22), including oesophageal atresia [9,50,51], anorectal malformations [52], congenital diaphragmatic hernia (CDH) [18,23,51,53,54], biliary atresia [36–39,55–64], and Hirschsprung disease [24]; abdominal wall defects (*n* = 1 [21]); chromosomal anomalies (*n* = 12), including trisomy 21 [14,19,22,65–69,70,71], trisomy 13 [25,72], and trisomy 18 [25,72]; skeletal dysplasias (*n* = 2 [13,20]); and Prader-Willi syndrome (PWS) (*n* = 1 [73]). The included studies were conducted in Europe (*n* = 29 [8,9,13–15,17,21–24,36–39,44,45,50–54,56–58,60,61,64,65,68]), the United States of America (*n* = 12 [7,18–20,40,41,43,46–48,70,72]), Australia (*n* = 7 [16,42,59,66,67,69,73]), Canada (*n* = 3 [6,25,63]), Japan (*n* = 1 [62]), Brazil (*n* = 1 [55]), and Hong Kong (*n* = 1 [71]). One international study reported survival of children with spina bifida from a number of registries from Europe and the USA [49]. As all included studies were population-based, sources of case ascertainment for most studies (*n* = 39) were congenital anomaly registers or surveillance programmes that either included all types of major congenital anomalies or were anomaly-specific. The majority of these studies linked their congenital anomaly data with death registration data to ascertain data on age at death (S4 Table).

**Table 1:** Description of included studies.

Author, publication year, reference, location	Congenital anomaly (CA) group/subtype	Birth year period	Duration and completeness of follow-up (FU)	Inclusion of additional anomalies/exclusions	Reporting of survival estimates	Study quality total score*
Agha, 2006 [6], Ontario, Canada	All anomalies and by group	1979–1986	10 years for all anomalies	Multiple births excluded	1- and 5-year estimates by CA group reported, 10-year survival for all CAs extracted from Kaplan-Meier (K-M) curves	9
Bakker, 2019 [49], 5 European and 4 USA registries‡	Spina bifida International Classification of Diseases Revision 10 (ICD-10) Q05 and ICD-9 741	2001–2012 (for 7 out of 8 included registers)	Up to 5 and ≥5 years, depending on the registry	Only registries with FU beyond 1 year and using linkage to vital records (*n* = 9) are included in this review. Cases excluded when present with anencephaly. Both isolated and syndromic cases are included	Survival estimates calculated using mortality rates reported	6
Bell, 2016 [16], Western Australia (WA)	Orofacial clefts	1980–2010	FU to 20 years for 1980–1992, low loss to FU (approximately 2.8%)	Estimates for isolated and those with additional CA	1-year estimates by cleft type (for 1980–2010 cohort) and 20-year estimates (for 1980–1992) reported	8
Berger, 2003 [7], Michigan, USA	All anomalies (not stratified by group)	1992–1998	Up to 7 complete years of FU (for those born in 1992, 97%)	Multiple births excluded	Reported mortality for each birth year, survival estimated by reviewer	8
Borgstedt-Bakke, 2017 [45], western Denmark	Spina bifida (myelomeningocele)	1 Jan 1970 to 30 Jun 2015	Up to 20 years, censored on 9 Nov 2015; median age at death: 1 year of age	Excluded cases with incomplete mortality or clinical data (*n* = 16)	Survival estimates extracted from K-M curves by birth year period: 1970–1979, 1980–1989, and 1990–2015	7
Brodwall, 2018 [22], Norway	Down syndrome (DS)	1994–2009	Complete FU to 5 years for those traced (5.5% lost to FU—censored)	Isolated DS and with associated (congenital heart defect [CHD] and/or extracardiac malformation) anomalies included	K-M survival estimates reported in the paper or obtained from authors on request	8
Burgos, 2017 [23], Sweden	Congenital diaphragmatic hernia (CDH)	1987–2013	FU up to 20 years for the whole period, up to 10 years for 2000–2013, complete for 98.7%	Patients who were diagnosed of CDH after the neonatal period were excluded	1-year and overall (beyond 1 year) mortality reported; 1-, 5-, and 10-year survival extracted from K-M curves	6
Cassina, 2016 [50], Northeast Italy (NEI)	Oesophageal atresia (ICD-9 750.3)	1981 to 31 July 2012	FU up to age 25 years (minimum 3 months) or censored at 31 Oct 2012, survival traced in 91.7% (330/360)	Chromosomal anomalies (*n* = 42, 10.3%) excluded, other non-isolated cases included	Survival estimates reported for 1 and 25 years, for 5 and 10 years extracted from K-M curves	6
Cassina, 2019 [52], NEI	Anorectal malformations	1981–2014	Survival status was traced for patients born between 1 Jan 1990 and 31 Jul 2012 up to 20 years (88.2%)	Those with non-isolated anomalies were included (*n* = 216, 50.5%), isolated (*n* = 212) included 7 patients with trisomy 21	Overall K-M survival estimates (with 95% confidence interval [95% CI]) reported for 1 and 20 years, for 10 years separately for isolated and non-isolated	5
Chardot, 2013 [36], France	Biliary atresia (BA)	1986–2009	Median FU in survivors 9.5 years (range 3 months to 24.6 years)	Only cases with corrected diagnosis of BA, including those with BA splenic malformation syndrome (BASM)	K-M survival estimates reported for 5, 10, 15, and 20 years, 95% CI calculated using reported SE	6
Chua, 2020 [71], Hong Kong	DS (ICD-9 code 758.0)	1995–2014	FU from birth until the age of 5 years, up to 30 Jun 2017, or the date of death (FU range 0.01–22.0 years)	All with DS, with or without associated anomalies	K-M survival estimates reported for 6 months, 1 and 5 years	6
Dastgiri, 2003 [17], Glasgow, Scotland	All anomalies and by group	1980–1997	5 years’ FU for all (97% complete)	Isolated anomalies only included	K-M survival estimates reported for 1 and 5 years and 95% CI provided by authors on request	6
Davenport, 2011 [37], England and Wales	BA	1999–2009	Vital status assessed in Jan 2010—up to 10 years of age, none lost to FU	BA cases with BASM and other associated anomalies (*n* = 84) included	Actuarial survival estimates reported for 5 and 10 years, extracted from survival curve for 4 years	6
De Carvalho, 2010 [55], Brazil	BA	Jul 1982 to Dec 2008	FU between Jul 1982 and Dec 2008, loss to FU not reported	BA cases with BASM or other associated anomalies (*n* = 61) included	K-M survival estimates (without 95% CI) reported for 4 years	5
De Vries, 2011 [56], the Netherlands	BA	1977–1988	20-year FU: median 23.8 (range 20.2–31.4), 2 lost to FU	All BA cases (including BASM, *n* = 7) included, no other anomalies reported	20-year survival reported	6
Eide, 2006 [8], Norway	All anomalies and by selected subgroup	1967–1979; FU 1967–1998	FU 18 years for all birth years, 6.2% (*n* = 24,355) untraceable from the whole cohort of 393,570	Male patients and live singleton births only included. CAs ascertained during the first week after birth only, selection bias possible	No survival analysis performed, mortality by age 18 years (military draft) reported, survival estimated by reviewers assuming no censoring	8
Folkestad, 2016 [13], Denmark	Osteogenesis imperfecta (OI)	1977–2012	FU to 31 Dec 2013, up to 20 years (for this review)	All patients with OI diagnosis on National Patient Register included, survival up to 20 years for patients born from 1977 included in this review	Survival estimated by reviewers using data on deaths and number at risk provided by authors on request	9
Frid, 1999 [65], northern Sweden	DS	1973–1980, FU 1973–1997	Complete FU to age 14.5 years (*n* = 213, 95.1%)	All with DS, with or without associated anomalies	Mortality reported, survival estimated by reviewers	6
Garne, 2002 [51], Funen County, Denmark	Gastrointestinal anomalies (atresias, abdominal wall defects, and CHD)	1980–1993, FU 1980–98	FU of all patients to 5 years of age	All patients with and without associated anomalies	Number of deaths and survivors reported, survival estimated by reviewers	6
Glasson, 2016 [66], WA	DS	1980–2010, censored to end 2013	FU to 31 Dec 2013, up to 25 years for birth years 1980–2010	From the survival analysis, deaths within the first 24 hours excluded (*n* = 11)	1-, 5-, 10-, 20-, and 25-year K-M survival estimates with 95% CI reported	7
Grizelj, 2010 [57], Croatia	BA	1992–2006	FU to 31 Dec 2006, (median 2.65 years, range 0.2–14.3) (6.9% [2/29] lost to FU)	1 inoperable patient excluded from survival analysis	K-M 5- and 10-year native liver survival (NLS) estimates with 95% CI reported; all deaths included by reviewers for the overall survival	6
Gudbjartsson, 2008 [53], only Iceland centre included	CDH	1983–2002	FU 1983 to Apr 2005, 3-year FU of all patients (mean FU 5 years)	Only early presenters (diagnosed within first 24 hours, *n* = 19) included	3-year survival reported for early presenters, overall survival estimated by reviewers (*n* = 23)	6
Halliday, 2009 [67], Victoria, Australia	DS	2 birth cohorts: 1988–1990 and 1998–2000	FU to 2005, 5-year FU for all births (unless the child died interstate; percentage of migration < 2%)	Patients with associated anomalies (*n* = 121 in 1988–1990 and *n* = 89 in 1998–2000) included	K-M 5-year survival reported, 1-year survival estimated by reviewers	6
Hayes, 1997 [68], Dublin, Ireland	DS	1980–1989	FU data collected in 1992 (range 3–12 years) (vital status unavailable in 1.3%, *n* = 5)	Patients with associated anomalies (*n* = 212) included (data on additional CAs available in 365/389, 93.6%)	K-M survival reported for 1980–1989, and for 1980–1994 and 1985–1989	6
Hinton, 2017 [18], Atlanta, USA	CDH	1979–2003	FU to death or censored at 31 Dec 2006; 3-year survival complete for all cases	Excluded children with known chromosomal anomalies or syndromes	K-M overall survival reported by various factors, K-M survival curves plotted for White and Black ethnicity by birth period, poverty, and CHD	6
Jaillard, 2003 [54], France	CDH	1991–1998	FU to 2 years of all the surviving infants with CDH	Patients with associated lethal CAs (*n* = 9) excluded	Early (<2 months) and late deaths (between 2 months and 3 years) reported, 2-year survival with 95% CI estimated by reviewers	6
Kucik, 2013 [19], 10 regions, USA	DS	1983–2003	FU ranged from 9 to 22 years between the regions (8 regions with up to 11+ years, 4 with 20–22 years)	Cases with additional anomalies (e.g., CHD) included	K-M survival estimates with 95% CI reported for 1, 5, 10, and 20 years	6
Lampela, 2012 [60], Finland	BA	1987–2010	FU to 4 full years for all live births with BA	All BA cases included: with BASM (*n* = 9, 14%), with other anomalies (*n* = 6, 9%)	Actuarial 4-year survival estimates reported and final figures provided by author on request, 95% CI calculated by reviewers	6
Leonard, 2000 [69], WA	DS	1980–1996	FU to 10 years for all born in 1980–1985, to 10 years for 1986–1990, and to 5 years for 1991–1996	Cases with additional anomalies (e.g., CHD) included	K-M 1-, 5-, and 10-year survival estimates reported, overall and by 3 birth periods	6
Leonhardt, 2011 [61], Germany	BA	2001–2005	FU to 2 full years (16/183 lost to FU, 8.7%)	All with BA diagnosis included	2-year K-M survival estimates after Kasai hepatoportoenterostomy (KP) or liver transplantation reported, overall survival (including 3 initial deaths) calculated by reviewers	5
Lionti, 2012 [73], Victoria, Australia	Prader-Willi syndrome (PWS)	1950 to 31 May 2010	FU to 35 years of age, loss to FU not reported	Only patients with diagnosed PWS included, infant deaths may have been missed by the register	K-M survival estimates with 95% CI reported for 10, 20, 30, and 35 years, estimates for 1, 5, 15, and 25 years extracted from K-M curves	5
Löf Granström, 2017 [24], Sweden	Hirschsprung disease (HSCR)	1964–2013	FU to 31 Dec 2013 (up to 50 years of age), median 19 years (range 2–49), loss to FU not reported†	Only those with confirmed diagnosis of HSCR included (*n* = 739), those with HSCR and DS also included	K-M survival curves with 95% CI presented up to 50 years, survival estimates up to 25 years extracted by reviewers	8
McKiernan, 2000 [39], UK and Ireland	BA	Mar 1993 to end Feb 1995	FU up to 5 years (median 3.5 years, range 0.3–5.4), lost to FU 2.2%	Those with additional CAs included (*n* = 20, *n* = 9 BASM)	Actuarial survival estimated by K-M method and 5-year overall survival and NLS reported	6
McKiernan, 2009 [38], UK and Ireland	BA	Mar 1993 to end Feb 1995	FU: median age at last FU 12 years (range 0.25–14), only 2 lost to FU (2.2%)	Those with additional CAs included (*n* = 20, *n* = 9 BASM)	Actuarial survival estimated by K-M method and 13-year overall survival and NLS reported	6
Meyer, 2016 [72], 9 states, USA	Trisomy 13 and trisomy 18	1999–2007	FU 1999–2008, birth years 1999–2005 included for survival estimation to 5 years, loss to FU not reported†	All cytogenetic variants included; different birth years included in different states	K-M survival estimates with 95% CI (<28 days, <1 year, and <5 years) reported	6
Nelson, 2016 [25], Ontario, Canada	Trisomy 13 and trisomy 18	1991–2012	FU 1991–2013, up to 7,000 days (1.6%, *n* = 7 lost to FU)	All cytogenetic variants included (90.2% unspecified, 3.5% mosaic, 6.3% translocation)	K-M survival estimates with 95% CI for 1, 5, and 10 years reported	6
Nembhard, 2010 [43], Texas, USA	All CAs, not stratified by group	1996–2003	FU to 2005, 5-year survival analysed; loss to FU not reported†	3.7% (unduplicated *n* = 1,877) excluded: trisomy 13 or 18 (*n* = 511); not born to non-Hispanic White (NHW), non-Hispanic Black (NHB), or Hispanic mother (*n* = 1,340); deaths with no date of death (*n* = 50)	5-year K-M survival estimates with 95% CI for NHW, NHB, and Hispanic ethnicity for term and preterm births reported and by size at birth	6
Nio, 2003 [62], Japan	BA	1989–1999	1989 only: compete FU for 10-year survival; 1989–1994: complete FU for 5-year survival, 2.6% lost to FU (*n* = 19)	BA cases with additional anomalies included (19.6% including *n* = 33 with BASM)	5- and 10-year survival estimates reported only for those birth years with complete FU	6
Oddsberg, 2012 [9], Sweden	Oesophageal atresia	1964–2007	Complete FU of the nationwide cohort by birth year, up to 25 years for 1964–1969 (percentage missing negligible)	Patients older than 1 year at diagnosis excluded to avoid misclassification; cases with associated CAs included	K-M survival estimates up to 20 years by time period extracted from K-M curves by reviewers	9
Pakarinen,2018 [58], Nordic countries	BA	1 Jan 2005 to 30 Jun 2016	FU for at least 4 months, median 4.9 (IQR 1.8–7.9 years)	Noncurable CHD or central nervous system CA (*n* = 4) withdrawn from treatment and excluded from the survival analysis, other associated CAs (*n* = 41, BASM *n* = 19) included	K-M 5- and 10-year survival estimates reported for 154 included cases, survival estimated by reviewers based on all 158 BA patients for consistency	6
Rankin, 2012 [14], Northern England	DS	1985–2003	FU to 29 Jan 2008, 95.3% traced (669/702)	All live-born patients with DS—full trisomy 21, mosaicism, and translocation—were included	K-M survival estimates with 95% CI reported for 1, 5, 10, and 20 years	6
Rasmussen, 2006 [70], Metropolitan Atlanta, USA	DS	1979–1998	1979–1999, FU complete for 1979–1988 for 10-year survival, censored by 20 years (loss to FU not reported†)	47 (of 692) excluded: cytogenetic results unavailable (22), complex rearrangements involving chromosome 21 (7), mosaicism (16), and not DS (2)	K-M survival estimates with 95% CI reported for 1 and 10 years, 5- and 20-year estimates with 95% CI extracted from K-M curves by reviewers	6
Risby, 2017 [21], southern Denmark	Gastroschisis	1997–2009	FU to 5 years for the whole cohort (between Jun 2013 and Apr 2014)	All cases with gastroschisis included	1- and 5-year survival estimated by reviewers using mortality data	6
Schneuer, 2019 [42], New South Wales (NSW), Australia	All anomalies, by group and subtype by European Surveillance of Congenital Anomalies (EUROCAT) classification	2004–2009	FU to death, 5 years of age, or until 31 Mar 2014, whichever came first	Excluded cases without linked birth records (*n* = 701), mothers residents outside NSW (*n* = 110), born at 19 weeks of gestation (*n* = 3)	K-M 1- and 5-year survival estimates with 95% CI reported	6
Schreiber, 2007 [63], Canada	BA	1985–2002	FU up to 10 years, 7% missing survival data for 1985–1995, no missing for 1996–2002	All with confirmed diagnosis of BA included, including 27 (14%) with BASM phenotype	K-M survival estimates with 95% CI reported for 4 and 10 years	6
Shin, 2012 [46], 10 regions, USA	Spina bifida:	1979–2003	FU to 2004 (up to 20 years for 1983–2003) for 8 registries, loss to FU not reported†	Cases with associated anomalies (e.g., major CHD) included	K-M 1-, 5-, and 20-year survival reported for 1983–2003; other: extracted from K-M curves by reviewers	6
Siffel, 2003 [47], Atlanta, USA	Encephalocele	1979–1998	FU 1979–1999 (for survivors censored at 31 Dec 1999); loss to FU not reported†	Excluded 8 cases: trisomy 13 (1), trisomy 18 (1), amniotic bands (3), coded with ‘possible’ diagnosis (3); with other major CAs included (*n* = 17)	K-M survival estimates reported for 1, 5, and 20 years—overall and by risk factor	6
Simmons, 2014 [20], Texas, USA	Achondroplasia	1996–2005	FU to 31 Dec 2007 up to age 10 years (minimal 2-year FU for all patients), none lost to FU	All with confirmed diagnosis of achondroplasia included	Mortality reported, 2-year survival with 95% CI estimated by reviewers (no censoring, as all FU to age 2 years)	6
Sutton, 2008 [44], Dublin, Ireland	Spina bifida, encephalocele	1976–1987	Retrospective data collection between Aug 1989 and Apr 1990 for 5-year survival (1.1% [*n* = 6] lost to FU)	Excluded: those with anencephaly and with spina bifida occulta; infants lost to FU immediately after birth (*n* = 6)	K-M 1- and 5-year survival estimates (no 95% CI) reported	6
Tennant, 2010 [15], Northern England	All anomalies, by group and subtype	1985–2003	FU to 29 Jan 2008, up to 20 years; 99% traced (10,850/10,964)	Excluded individuals with unavailable data on survival status (114; 1%); those with chromosomal anomalies outside the EUROCAT range (ICD codes Q940-59)	K-M survival estimates with 95% CI reported for EUROCAT CA groups and subtypes for 1, 5, 10, 15, and 20 years	6
Tu, 2015 [59], South Australia	BA	1989–2000	The median FU period 13.4 years (IQR, 6.2–18.2; range 0.6–21), no loss to FU	Excluded 2 patients, as the initial KP was performed interstate	K-M 5-year survival estimates with 95% CI reported by authors for both overall survival and NLS	6
Wang, 2011 [40], New York state, USA	All anomalies and by group	1983–2006	FU to end 2008 for up to 25 years (assuming alive if no death by 31 Dec 2008), loss to FU not reported	Only Congenital Malformations Registry cases matched to their birth certificates (97%) included (*n* = 57,002), cases with additional anomalies included	K-M survival estimates with 95% CI reported for selected CA groups and subtypes for 1, 5, 15, and 25 years	5
Wang, 2015 [41], 12 states, USA	All anomalies and by group	1999–2007	FU to end 2008 (ranging from 1 to 9 years), loss to FU not reported	All live births with a major CA included (*n* = 98,833); infants with multiple defects were included in each relevant birth defect category	K-M survival estimates with 95% CI reported for selected CA groups and subtypes for <1, <2, and <8 years	5
Wildhaber, 2008 [64], Switzerland	BA	1994–2004	Median FU 58 months (range 5–124); no loss to FU	All patients, including those with associated anomalies, were included: BASM (*n* = 4), other associated anomalies or disease (*n* = 6)	K-M 5-year survival estimates (overall and NLS) with SE reported, 95% CI calculated by reviewers	6
Wong, 2001 [48], Atlanta, USA	Spina bifida	1979–1994	FU 1979–1996, loss to FU not reported†	Excluded cases associated with anencephaly or trisomies 13 or 18	K-M survival estimates with 95% CI to age 18 years (1, 5, 10, 15, 18)	6

*Study data quality was measured using Newcastle-Ottawa Quality Assessment Scale for cohort studies—maximum 9, maximum 6 for those with no comparison group/nonexposed cohort. Scores of <5 indicated high risk of bias [95].

^†^Loss to FU likely to be low as the linkage system for tracing deaths is well established (involving linkage with the National Death Index in the USA studies for deaths outside the state).

^‡^Data from Atlanta, USA, are not included, as they are part of the cohort used by Wang and colleagues [41].

As our literature search was restricted to years between January 1995 and April 2020, the publication years ranged between 1997 [68] and 2020 [71], whereas patients were mostly born between 1970 and 2010, with the earliest birth year in 1950 [73] and the latest ending in June 2016 [58]. Table 1 also describes the duration of follow-up, the survival age analysed, and whether survival was reported in the papers (with or without 95% CI) or estimated by our reviewers. Table 1 also gives the NOS scores that range between 5 and 8 respective of the use of the comparison group that is not mandatory for the survival studies (see S3 Table for detailed scoring). According to NOS, all studies were of low risk of bias.

### Survival of children with different congenital anomalies

Table 2 shows survival estimates overall and by birth cohort, where reported, for individuals up to 25 years of age for studies estimating survival for all congenital anomalies combined and by different group/type. S5 Table presents more detail for studies reporting survival estimates by other risk factors (e.g., ethnicity or presence of additional anomalies). Most studies reported 1- and 5-year survival estimates only. Survival varied considerably according to anomaly; therefore, survival estimates are presented by different groups and subtypes (Table 2). The 5-year survival for all anomalies combined varied from 85% to 95%, owing to different inclusion and exclusion criteria. It was not considered appropriate to pool survival estimates for all congenital anomalies combined, because of the diversity of the contributing congenital anomaly groups.

**Table 2:** Survival estimates by congenital anomaly type at age 1–25 years, overall and by birth cohort.

Congenital anomaly group/subtype	Study and birth year	*N* deaths/live births	Survival estimates percentage (95% confidence interval [95% CI])					
			1 year	5 years	10 years	15 years	20 years	25 years
All congenital anomalies								
International Classification of Diseases Revision 9 (ICD-9) codes 740.0–759.9	Agha, 2006 [6], 1979–1986, Canada	3620/45,200	93.4	92.5	***92*.*3***	**—**	**—**	**—**
ICD-9 codes 740–759	Berger, 2003 [7], 1992–1998, USA	2182/43,708	95.7	*95*.*0*	**—**	**—**	**—**	**—**
British Paediatric Association (BPA)-ICD-9 codes 740–759	Dastgiri, 2003 [17], 1980–1997, Scotland	740/6153	89.11	87.95	**—**	**—**	**—**	**—**
ICD-8 codes (740–759)	Eide, 2006 [8], 1967–1979, Norway	1169/9186	**—**	**—**	**—**	**—**	*87*.*4*^a^	**—**
ICD-9 740.00–758.090	Nembhard, 2010 [43], 1996–2003, USA	3518/48,391	*93*.*7*	*92*.*7*	**—**	**—**	**—**	**—**
ICD-10 (Q00-Q99)	Tennant, 2010 [15], 1985–2003, Northern England	1465/10,850	**—**	**—**	**—**	**—**	85.5 (84.8–86.3)	**—**
ICD-9 codes 740–759	Wang, 2011 [40], 1983–2006, USA	9112/57,002	87.1 (86.8–87.4)	85.2 (84.9–85.5)	**—**	83.9 (83.6–84.2)	**—**	82.7 (82.4–83.1)
Neural tube defects								
Including anencephaly	Dastgiri, 2003 [17], 1980–1997, Scotland	40/144	72.2 (64.9–79.5)^b^	71.5 (63.8–79.3)^b^	**—**	**—**	**—**	**—**
Including anencephaly	Schneuer, 2019 [42], 2004–2009, New South Wales (NSW), Australia	34/110	69.1 (60.5–77.7)	69.1 (60.5–77.7)	**—**	**—**	**—**	**—**
Including anencephaly	Tennant, 2010 [15], 1985–2003, Northern England	87/226	65.0 (58.4–70.9)	62.8 (56.2–68.8)	62.4 (55.7–68.3)	62.4 (55.7–68.3)	63.4 (53.4–66.7)	**—**
Excluding anencephaly	Sutton, 2008 [44], 1976–1987, Ireland	313/543	43.7	40.8	**—**	**—**	**—**	**—**
Anencephaly								
ICD-9 code 740.0–740.2	Agha, 2006 [6], 1979–1986, Canada	183/	4.8	4.6	**—**	**—**	**—**	**—**
	Schneuer, 2019 [42], 2004–2009, NSW, Australia	19/19	0.0	**—**	**—**	**—**	**—**	**—**
	Tennant, 2010 [15], 1985–2003, Northern England	17/17	0.0	**—**	**—**	**—**	**—**	**—**
ICD-9 740.0–740.1	Wang, 2011 [40], 1983–2006, USA	447/479	7.3 (5.2–9.9)	6.8 (4.8–9.3)	**—**	6.5 (4.5–9.0)	**—**	6.5 (4.5–9.0)
Spina bifida								
ICD-9 code 741.0–741.9	Agha, 2006 [6], 1979–86, Canada	182/	78.5	75.3	**—**	**—**	**—**	**—**
ICD-10 Q05 and ICD-9 741	Bakker, 2019 [49], 2001–2012, Czech Republic	/139	*91*.*4*	*90*.*0*	*88*.*6*^c^	**—**	**—**	**—**
	Malta Congenital Anomaly Registry	/28	*92*.*8*	*92*.*8*	**—**	**—**	**—**	**—**
	Sweden	/263	*92*.*5*	*92*.*1*	*91*.*7*^c^	**—**	**—**	**—**
	UK–Wales	/78	*91*.*0*	*89*.*7*	*89*.*7*^c^	**—**	**—**	**—**
	USA–Arkansas	/177	*87*.*0*	*84*.*2*	*83*.*1*^c^	**—**	**—**	**—**
	USA–Texas	/1,578	*91*.*6*	*90*.*5*	*90*.*1*^c^	**—**	**—**	**—**
	USA–Utah	/213	*90*.*7*	*90*.*7*	*90*.*2*^c^	**—**	**—**	**—**
	USA–Atlanta, 2001–2008	/112	*95*.*5*	*95*.*5*	*95*.*5*^c^	**—**	**—**	**—**
	Italy–Lombardy, 2003–2012	/25	100.0	96.0	**—**	**—**	**—**	**—**
Myelomeningocele	Borgstedt-Bakke, 2017 [45], 1970–1979, Denmark	16/58	***84*.*5***	***84*.*5***	***82*.*8***	***79*.*4***	***79*.*4***	**—**
	1980–1989	5/39	***97*.*5***	***92*.*4***	***92*.*4***	***92*.*4***	***89*.*8***	**—**
	1990–2015	6/90	***95*.*6***	***95*.*6***	***94*.*5***	***92*.*8***	***92*.*8***	**—**
Spina bifida (ICD-8 code 741)	Eide, 2006 [8], 1967–79, Norway	56/113	**—**	**—**	**—**	**—**	*50*.*4*^a^	**—**
Spina bifida	Schneuer, 2019 [42], 2004–2009, NSW, Australia	11/56	80.4 (70.0–90.8)	80.4 (70.0–90.8)	**—**	**—**	**—**	**—**
ICD-9 741.0 and 741.9	Shin, 2012 [46], 1997–2003, USA	162/2,259	92.8 (91.7–93.8)	**—**	**—**	**—**	**—**	**—**
	1983–1987		***87*.*1***	***84*.*5***	***82*.*7***	***80*.*7***	***80*.*4***	**—**
	1988–1992		***90*.*4***	***87*.*6***	***86*.*7***	***85*.*7***	**—**	**—**
	1993–1997		***89*.*9***	***88*.*2***	***87*.*2***	**—**	**—**	**—**
	1998–2003		***92*.*8***	***90*.*8***	**—**	**—**	**—**	**—**
Myelomeningocele and spinal meningocele	Sutton, 2008 [44], Ireland	/373	50.4	47.3	**—**	**—**	**—**	**—**
Spina bifida, ICD-10 Q05	Tennant, 2010 [15], 1985–2003, Northern England	63/195	70.8 (63.8–76.6)	69.2 (62.2–75.2)	68.7 (61.6–74.7)	68.7 (61.6–74.7)	66.4 (58.9–72.9)	**—**
ICD-9 741.0, 741.9	Wang, 2011 [40], 1983–2006, USA	324/1999	88.5 (87.0–89.8)	86.4 (84.8–87.8)	**—**	83.8 (82.0–85.4)	**—**	82.2 (80.1–84.0)
Spina bifida without anencephaly	Wang, 2015 [41], 1999–2007, USA	318/3903	91.9 (90.9–92.7)	**—**	90.2 (89.0–91.2)^d^	**—**	**—**	**—**
	Wong, 2001 [48], USA, 1979–1994	45/235	87.2 (83.1–91.6)	83.8 (79.2–88.6)	80.9 (75.8–86.3)	78.4 (72.4–84.7)	78.4 (72.4–84.7)^a^	**—**
	1979–1983		83 (75–91)	82 (73–90)	79 (71–88)	**—**	76 (68–86)^a^	**—**
	1984–1988		89 (92–96)	85 (78–93)	81 (73–90)	**—**	**—**	**—**
	1989–1994		91 (85–98)	84 (75–94)	**—**	**—**	**—**	**—**
Encephalocele								
	Siffel, 2003 [47], 1979–1998, USA	25/83	72.2 (62.6–81.9)	70.8 (60.9–80.7)	**—**	**—**	67.3 (55.7–78.8)	**—**
	Sutton, 2008 [44], 1976–1987, Ireland	/64	32.9	27.3				
	Tennant, 2010 [15], 1985–2003, Northern England	7/14	64.3 (34.3–83.3)	50.0 (22.9–72.2)	50 (22.9–72.2)	50 (22.9–72.2)	**—**	**—**
ICD-9 742.0	Wang, 2011 [40], 1983–2006, USA	171/556	75.7 (71.9–79.1)	72.1 (68.1–75.6)	**—**	69.7 (65.6–73.4)	**—**	67.2 (62.7–71.3)
	Wang, 2015 [41], 1999–2007, USA	254/909	72.1 (69.0–74.9)	**—**	69.9 (66.1–73.3)^d^	**—**	**—**	**—**
Hydrocephalus								
	Eide, 2006 [8], 1967–1979, Norway	29/59	**—**	**—**	**—**	**—**	*50*.*8*^a^	**—**
	Schneuer, 2019 [42], 2004–2009, NSW, Australia	15/60	75.0 (64.0–86.0)	75.0 (64.0–86.0)	**—**	**—**	**—**	**—**
	Tennant, 2010 [15], 1985–2003, Northern England	32/108	76.9 (67.8–83.7)	75.0 (65.7–82.1)	71.2 (61.3–79.0)	69.8 (59.6–77.8)	66.4 (54.5–75.9)	**—**
742.3	Wang, 2011 [40], 1983–2006, USA	1,314/5,378	82.7 (81.6–83.7)	78.5 (77.4–79.6)	**—**	75.3 (74.1–76.5)	**—**	73.4 (72.1–74.7)
Orofacial clefts								
Cleft palate and cleft lip (749.0–749.9)	Agha, 2006 [6], 1979–1986, Canada	188/	90.2	88.2	**—**	**—**	**—**	**—**
Orofacial clefts (749.0–749.9)	Bell, 2016 [16], 1980–2010, Western Australia	113/1,509	92.5 *(91*.*0–93*.*8)*	**—**	**—**	**—**	**—**	**—**
Orofacial clefts	1980–1992	73/585	**—**	*87*.*5 (84*.*5–90*.*0)*	**—**	**—**	**—**	**—**
Cleft lip only (BPA-ICD-9–749.10–749.19)	1980–2010 for 1 year, 1980–2007 for 5 years; 1980–1992 for 20 years		95.8 (all) 99.7 (isolated)	*95*.*8* (all) 99.7 (isolated)	**—**	**—**	97.7 (all) 100.0 (isolated)	**—**
Cleft lip and palate (749.20–749.27, 749.29)	1980–2010 for 1 year, 1980–2007 for 5 years, 1980–1992 for 20 years		91.2 (all) 99.1 (isolated)	*99*.*1* (isolated)	**—**	**—**	84.5 (all); 98.0 (isolated)	**—**
Cleft palate (749.00–749.09)	1980–2010 for 1 year, 1980–1992 for 20 years		91.7 (all) 99.2 (isolated)	**—**	**—**	**—**	83.5 (all); 97.2 (isolated)	**—**
Cleft lip with/without palate	Dastgiri, 2003 [17], 1980–1997, Scotland	5/278	98.2 (96.8–99.6)^b^	98.2 (96.6–99.8)^b^	**—**	**—**	**—**	**—**
Cleft lip	Eide, 2006 [8], 1967–1979, Norway	6/250	**—**	**—**	**—**	**—**	*97*.*6*^a^	**—**
Cleft palate		9/151	**—**	**—**	**—**	**—**	*94*.*0*^a^	**—**
Cleft lip and palate		19/357	**—**	**—**	**—**	**—**	*94*.*7*^a^	**—**
Orofacial clefts	Schneuer, 2019 [42], 2004–2009, NSW, Australia	7/575	99.0 (98.1–99.8)	98.8 (97.9–99.7)	**—**	**—**	**—**	**—**
Cleft lip and palate		0/188	100.0	100.0	**—**	**—**	**—**	**—**
Orofacial clefts	Tennant, 2010 [15], 1985–2003, Northern England	14/584	97.8 (96.2–98.7)	97.8 (96.2–98.7)	97.6 (95.9–98.6)	97.6 (95.9–98.6)	97.6 (95.9–98.6)	**—**
Cleft lip		1/140	99.3 (95.0–99.9)	99.3 (95.0–99.9)	99.3 (95.0–99.9)	99.3 (95.0–99.9)	99.3 (95.0–99.9)	**—**
Cleft lip and palate		5/227	98.2 (95.4–99.3)	98.2 (95.4–99.3)	97.7 (94.6–99.1)	97.7 (94.6–99.1)	97.7 (94.6–99.1)	**—**
Cleft palate		8/217	96.3 (92.8–98.1)	96.3 (92.8–98.1)	96.3 (92.8–98.1)	96.3 (92.8–98.1)	96.3 (92.8–98.1)	**—**
Cleft lip with or without cleft palate		6/367	98.6 (96.8–99.4)	98.6 (96.8–99.4)	98.3 (96.3–99.2)	98.3 (96.3–99.2)	98.3 (96.3–99.2)	**—**
Cleft palate without cleft lip (ICD-9 749.0)	Wang, 2011 [40], 1983–2006, USA	410/3,719	91.0 (90.0–91.8)	89.6 (88.6–90.6)	**—**	88.9 (87.8–89.9)	**—**	88.3 (87.1–89.4)
Cleft lip with/without cleft palate (ICD-9 749.1–749.2)		454/4,691	91.7 (90.9–92.5)	90.8 (89.9–91.6)	**—**	90.2 (89.3–91.0)	**—**	90.0 (89.1–90.8)
Cleft palate without cleft lip	Wang, 2015 [41], 1999–2007, USA	660/7,356	91.0 (90.4–91.7)	**—**	90.3 (89.5–91.1)^d^	**—**	**—**	**—**
Cleft lip with or without cleft palate		999/11,862	91.6 (91.1–92.1)	**—**	90.8 (90.1–91.4)^d^	**—**	**—**	**—**
Digestive system anomalies								
Oesophageal atresia								
ICD-9 code 750.3	Cassina, 2016 [50], 1981–2012 (all), Northeast Italy	/330	88.4 (84.9–91.9)	**—**	**—**	**—**	**—**	85.1 (80.8–89.4)
	1981–1996 (isolated)		***96*.*1***	***94*.*6***	***94*.*6***	***90*.*6***	***90*.*6***	***90*.*6***
	1997–2012 (isolated)		***95*.*3***	***95*.*3***	***95*.*3***	***95*.*3***	**—**	**—**
	1981–1996 (non-isolated)		63.0 (49.1–76.9)^e^	***58*.*7 (44*.*4–73*.*0)***	58.7 (44.4–73.0)^e^	***58*.*7 (44*.*4–73*.*0)***	***58*.*7 (44*.*4–73*.*0)***	58.7 (44.4–73.0)
	1997–2012 (non-isolated)		88.4 (82.7–94.1)^e^	***87*.*3 (81*.*2–93*.*4)***	87.3 (81.2–93.4)^e^	***87*.*3 (81*.*2–93*.*4)***	**—**	**—**
	Garne, 2002 [51], Denmark	11/27	—	59.3 (*39*.*0–77*.*0*)	—	—	—	—
ICD-7 756.21, ICD-8 750.20, 750.28, ICD-9 750D, ICD-10 Q39.0–Q39.2.	Oddsberg, 2012 [9], 1964–2007, Sweden	227/1,126	*82*.*1*	*80*.*7*	*80*.*6*	*80*.*5*	*80*.*1*	
	1964–1969		*62*.*1*	***62*.*1***	***62*.*1***	***62*.*1***	***58*.*5***	***58*.*5***
	1970–1979		***77*.*2***	***75*.*6***	***75*.*6***	***75*.*2***	***75*.*2***	***75*.*2***
	1980–1989		*82*.*5*	***82*.*1***	***81*.*9***	***81*.*9***	***80*.*5***	—
	1990–1999		*86*.*1*	***85*.*1***	***85*.*1***	***84*.*9***	—	—
	2000–2007		***87*.*8***	***87*.*6***	—	—	—	—
	Schneuer, 2019 [42], 2004–2009, NSW, Australia	0/51	100.0	100.0	—	—	—	—
	Tennant, 2010 [15], 1985–2003, northern England	7/105	95.2 (88.9–98.0)	93.3 (86.5–96.8)	93.3 (86.5–96.8)	93.3 (86.5–96.8)	93.3 (86.5–96.8)	—
ICD-9 750.3	Wang, 2011 [40], 1983–2006, USA	336/1,580	81.5 (79.5–83.4)	79.5 (77.4–81.4)	—	78.6 (76.4–80.5)	—	78.3 (76.1–80.3)
	Wang, 2015 [41], 1999–2007, USA	476/3,084	84.6 (83.2–85.8)	—	83.8 (82.1–85.2)^d^	—	—	—
Anorectal malformations								
ICD-9/BPA 752.1–752.4, cloaca—751.55	Cassina, 2019 [52], Northeast Italy, 1990–2012	/253	89.7 (85.2–92.9)	—	—	—	86.7 (81.6–90.4)	—
Anorectal atresia or stenosis								
	Tennant, 2010 [15], 1985–2003, Northern England	2/83	98.8 (91.8–99.8)	98.8 (91.8–99.8)	98.8 (91.8–99.8)	96.6 (86.1–99.2)	96.6 (86.1–99.2)	—
ICD-9 751.2	Wang, 2011 [40], 1983–2006, USA	374/2,654	87.7 (86.4–88.9)	86.5 (85.2–87.8)	—	85.9 (84.5–87.2)	—	84.8 (83.1–86.4)
	Wang, 2015 [41], 1999–2007, USA	702/5,400	87.0 (86.1–87.9)	—	86.1 (85.0–87.2)^d^	—	—	—
Hirschsprung disease								
ICD-7: 756.31, ICD-8: 751.39, ICD-9: 751D, ICD-10: Q431	Löf Granström, 2017 [24], 1964–2013, Sweden	22/739	***99*.*3 (98*.*7–99*.*8)***	***98*.*3 (97*.*4–99*.*2)***	***98*.*3 (97*.*4–99*.*2)***	***97*.*9 (96*.*9–99*.*0)***	***97*.*7 (96*.*5–98*.*8)***	***97*.*7 (96*.*5–98*.*8)***
	Schneuer, 2019 [42], 2004–2009, NSW, Australia	5/90	96.7 (93.0–100)	94.4 (89.7–99.2)	—	—	—	—
	Tennant, 2010 [15], 1985–2003, Northern England	4/61	93.4 (83.5–97.5)	93.4 (83.5–97.5)	93.4 (83.5–97.5)	93.4 (83.5–97.5)	93.4 (83.5–97.5)	—
Biliary atresia								
Overall survival								
	Chardot, 2013 [36], 1986–2009, France	228/1,107	**—**	80.8 (78.4–83.2)	79.7 (77.2–82.2)	78.6 (75.9–81.3)	77.6 (74.5–80.7)	**—**
	1986–1996		**—**	72.1 (68.0–76.2)	**—**	**—**	**—**	**—**
	1997–2002		**—**	88.0 (84.1–91.9)	**—**	**—**	**—**	**—**
	2003–2009		**—**	88.5 (84.8–92.2)	**—**	**—**	**—**	**—**
	Davenport, 2011 [37], 1999–2009, England and Wales	41/443	**—**	90 (88–93)	89 (86–93)	**—**	**—**	**—**
	De Carvalho, 2010 [55], 1982–2008, Brazil	166/513	**—**	67.6^f^	**—**	**—**	**—**	**—**
	De Vries, 2011 [56], the Netherlands							
	1977–1982	32/49	**—**	**—**	**—**	**—**	34.7 (*22*.*1–49*.*7)*	
	1983–1988	27/55	**—**	**—**	**—**	**—**	50.9 (*37*.*2–64*.*5)*	
	Grizelj, 2010 [57], 1992–2006, Croatia	7/29	**—**	*75*.*9 (56*.*1–89*.*0)*	*75*.*9 (56*.*1–89*.*0)*	**—**	**—**	**—**
	Lampela, 2012 [60], 1987–2010, Finland	27/72	**—**	62.5 (*50*.*3–73*.*4*)^h^	**—**	**—**	**—**	**—**
	Leonhardt, 2011 [61], 2001–2005, Germany	31/183	*81*.*9 (75*.*4–87*.*0)*^k^	**—**	**—**	**—**	**—**	**—**
	McKiernan, 2000 [39], 1993–1995, UK and Ireland	14/93	**—**	85.0 (77.7–92.3)	—	**—**	**—**	**—**
	McKiernan, 2009 [38], UK and Ireland	15/93	—	—	83.8 (76.2–91.4)^l^	**—**	**—**	**—**
	Nio, 2003 [62], Japan							
	1989 birth year	35/108	**—**	**—**	66.7	**—**	**—**	**—**
	1989–1994	182/735	**—**	75.3	**—**	**—**	**—**	**—**
	Pakarinen, 2018 [58], 2005–2016, Nordic countries	21/158	**—**	87.3 (80.9–91.9)	86.7 (80.2–91.4)	**—**	**—**	**—**
	Schreiber, 2007 [63], Canada							
	1985–2002	81/349		77 (72–92)^h^	75 (70–80)	**—**	**—**	**—**
	1985–1995	55/199		74 (67–79)^h^	**—**	**—**	**—**	**—**
	1996–2002	26/150		82 (75–88)^h^	**—**	**—**	**—**	**—**
	Tennant, 2010 [15], 1985–2003, Northern England	3/14	85.7 (53.9–96.2)	85.7 (53.9–96.2)	**—**	**—**	**—**	**—**
	Tu, 2015 [59], 1989–2000, South Australia	13/29	—	89.7 (71.5–97.3)	—	—	—	—
	Wildhaber, 2008 [64], 1994–2004, Switzerland	4/48	91.5 (83.5–99.5)^k^	91.5 (83.5–99.5)	91.5 (83.5–99.5)	—	—	—
Biliary atresia								
Survival with native liver (NLS)								
	Chardot, 2013 [36], 1986–2009, France	(99 + 542)^g^/1,035	**—**	40.0 (36.9–43.1)	35.8 (32.7–38.9)	32.1 (28.8–35.4)	29.6 (25.7–33.5)	**—**
	1986–1996		**—**	38.2 (32.9–43.5)	**—**	**—**	**—**	**—**
	1997–2002		**—**	43.1 (37.0–49.2)	**—**	**—**	**—**	**—**
	2003–2009		**—**	39.0 (32.5–45.5)	**—**	**—**	**—**	**—**
	Davenport, 2011 [37], 1999–2009, England and Wales	(24 + 179)^g^/424	**—**	46 (41–51)	40 (34–46)	**—**	**—**	**—**
	De Carvalho, 2010 [55], 1982–2008, Brazil	(94 + 165)^g^/392	**—**	36.8^h^	**—**	**—**	**—**	**—**
	De Vries, 2011 [56], the Netherlands							
	1977–1982	(31 + 8)^g^/49	**—**	**—**	**—**	**—**	20.4 (*10*.*7–34*.*8*)^g^	**—**
	1983–1988	(21 + 16)^g^/55	**—**	**—**	**—**	**—**	32.7 *(21*.*0–46*.*8*)^g^	**—**
	Grizelj, 2010 [57], 1992–2006, Croatia	(6 + 6)/28	**—**	51.7 (40.6–62.8)	38.8 (24.9–52.7)	**—**	**—**	**—**
	Lampela, 2012 [60], 1987–2010, Finland	(19 + 25)/72	**—**	38.9 (*27*.*8–51*.*1*)^h^	**—**	**—**	**—**	**—**
	Leonhardt, 2011 [61], 2001–2005, Germany	(28 + 105)/167	20.4 *(14*.*7–27*.*4)*^k^	**—**	**—**	**—**	**—**	**—**
	McKiernan, 2000 [39], 1993–95, UK and Ireland	(14 + 33)/93	**—**	*49*.*5 (39*.*0–60*.*0)*	**—**	**—**	**—**	**—**
	McKiernan, 2009 [38], UK and Ireland	(10 + 42)/93	**—**	**—**	43.8 (33.3–54.1)^l^	**—**	**—**	**—**
	Nio, 2003 [62], Japan							
	1989 birth year	51/108	**—**	**—**	52.8	**—**	**—**	**—**
	1989–1994	/735	**—**	59.7	**—**	**—**	**—**	**—**
	Pakarinen, 2018 [58], 2005–2016, Nordic countries	72/154	**—**	53 (45–62)	45 (35–55)	**—**	**—**	**—**
	Schreiber, 2007 [63], Canada	(81 + 169)/349		33 (28–38)^h^	24 (19–29)	**—**	**—**	**—**
	1985–1995	(55 + 98)/199		31 (31–38)^h^	**—**	**—**	**—**	**—**
	1996–2002	(26 + 71)/150		36 (28–45)^h^	**—**	**—**	**—**	**—**
	Tu, 2015 [59], 1989–2000, South Australia		—	55.2 (36.0–73.0)	—	—	—	—
	Wildhaber, 2008 [64], 1994–2004, Switzerland	(4 + 27)/48	40.5 (26.0–55.0)^k^	32.7 (18.6–46.8)	—	—	—	—
CDH^o^								
ICD-9 756.6, ICD-10 Q79.0 and Q79.1	Burgos, 2017 [23], 1987–2013 (all fatalities)	314/861	*65*.*4 (62*.*1–68*.*5)*	*63*.*5 (60*.*2–66*.*7)*^m^	**—**	**—**	**—**	**—**
	1987–1999 (all fatalities)	210/480		*56*.*3 (51*.*7–60*.*7)*^m^	**—**	**—**	**—**	**—**
	2000–2013 (all fatalities)	104/381		*72*.*7 (67*.*9–77*.*1)*^m^	**—**	**—**	**—**	**—**
	Garne, 2002 [51], 1980–1993	10/17	**—**	41.2 (*19*.*4–66*.*5*)	**—**	**—**	**—**	**—**
	Gudbjartsson, 2008 [53], 1983–2002, Iceland	8/23	**—**	*65*.*2 (42*.*8–82*.*8)*^j^	**—**	**—**	**—**	**—**
BPA code 756.610	Hinton, 2017 [18], 1979–2003, USA							
	Overall survival (up to 20 years, minimum of 3 years for all cases)							
	<1988	22/37	**—**	**—**	40.5 (23.4–57.6)	**—**	***40*.*5 (23*.*4–57*.*6)***	**—**
	≥1988	41/113	**—**	**—**	58.3 (46.0–70.6)	**—**	**—**	**—**
	Jaillard, 2003 [54], 1991–1998, France	34/85	*60*.*0 (48*.*9–70*.*3*)^j^	**—**	**—**	**—**	**—**	**—**
	Schneuer, 2019 [42], 2004–2009, NSW, Australia	24/90	73.3 (64.2–82.5)	73.3 (64.2–82.5)	**—**	**—**	**—**	**—**
	Tennant, 2010 [15], 1985–2003, Northern England	69/161	58.4 (50.4–65.6)	57.1 (49.1–64.4)	57.1 (49.1–64.4)	57.1 (49.1–64.4)	57.1 (49.1–64.4)	**—**
ICD-9 756.6	Wang, 2011 [40], 1983–2006, USA	586/1,541	63.5 (61.0–65.8)	62.6 (60.1–64.9)	**—**	62.1 (59.6–64.5)	**—**	61.4 (58.8–63.8)
	Wang, 2015 [41], 1999–2007, USA	1,017/3,248	68.7 (67.1–70.3)	**—**	68.0 (66.0–69.9)^d^	**—**	**—**	**—**
Limb anomalies								
Limb reduction defects								
	Schneuer, 2019 [42], 2004–2009, NSW, Australia	5/52	90.4 (82.4–98.4)	90.4 (82.4–98.4)	**—**	**—**	**—**	**—**
Upper-limb reduction								
	Tennant, 2010 [15], 1985–2003, Northern England	1/111	100.0	99.1 (93.8–99.9)	99.1 (93.8–99.9)	99.1 (93.8–99.9)	99.1 (93.8–99.9)	**—**
ICD-9 755.2	Wang, 2011 [40], 1983–2006, USA	199/1,752	90.7 (89.2–92.0)	89.4 (87.9–90.8)	**—**	89.0 (87.4–90.4)	**—**	87.7 (85.8–89.4)
	Wang, 2015 [41], 1999–2007, USA	387/3,602	89.3 (88.2–90.2)	**—**	88.2 (86.9–89.4)^d^	**—**	**—**	**—**
Lower-limb reduction								
	Tennant, 2010 [15], 1985–2003, Northern England	3/42	92.9 (79.5–97.6)	92.9 (79.5–97.6)	92.9 (79.5–97.6)	92.9 (79.5–97.6)	92.9 (79.5–97.6)	**—**
ICD-9 755.3	Wang, 2011 [40], 1983–2006, USA	136/1,044	88.6 (86.5–90.4)	87.3 (85.2–89.2)	**—**	87.1 (84.9–89.0)	**—**	86.7 (84.4–88.6)
	Wang, 2015 [41], 1999–2007, USA	219/1,913	88.6 (87.0–89.9)	**—**	88.2 (86.4–89.8)^d^	**—**	**—**	**—**
Abdominal wall defects								
Abdominal wall defects	Eide, 2006 [8], 1967–1979, Norway	72/206	**—**	**—**	**—**	**—**	*65*.*0*^a^	**—**
	Schneuer, 2019 [42], 2004–2009, NSW, Australia	14/139	90.6 (85.8–95.5)	89.9 (84.9–94.9)	**—**	**—**	**—**	**—**
Gastroschisis								
Surgical code DQ79.3, JAG10	Risby, 2017 [21], 1997–2009, South Denmark	7/71	*93*.*0 (83*.*7–97*.*4)*	*91*.*5 (81*.*9–96*.*5)*	**—**	**—**	**—**	**—**
	Schneuer, 2019 [42], 2004–2009, NSW, Australia	9/109	91.7 (86.6–96.9)	91.7 (86.6–96.9)	**—**	**—**	**—**	**—**
	Tennant, 2010 [15], 1985–2003, Northern England	12/190	93.7 (89.2–96.4)	93.7 (89.2–96.4)	93.7 (89.2–96.4)	93.7 (89.2–96.4)	93.7 (89.2–96.4)	**—**
ICD-9 756.73	Wang, 2011 [40], 1983–2006, USA	116/777	87.8 (85.3–89.9)	85.5 (82.8–87.8)	**—**	84.8 (82.0–87.2)	**—**	81.7 (74.0–87.3)
	Wang, 2015 [41], 1999–2007, USA	266/3,698	92.8 (91.9–93.6)	**—**	92.1 (91.0–93.2)^d^	**—**	**—**	**—**
Omphalocele								
	Tennant, 2010 [15], 1985–2003, Northern England	6/47	87.2 (73.8–94.1)	87.2 (73.8–94.1)	87.2 (73.8–94.1)	87.2 (73.8–94.1)	87.2 (73.8–94.1)	**—**
ICD-9 756.72	Wang, 2011 [40], 1983–2006, USA	200/639	69.5 (65.8–72.9)	68.8 (65.1–72.3)	**—**	68.6 (64.9–72.1)	**—**	68.6 (64.9–72.1)
	Wang, 2015 [41], 1999–2007, USA	367/1,281	71.4 (68.8–73.7)	**—**	71.2 (68.0–74.1)^d^	**—**	**—**	**—**
Urinary-system anomalies								
ICD-9 753.0–753.9	Agha, 2006 [6], 1979–1986, Canada	451/	68.8	67.2	**—**	**—**	**—**	**—**
	Dastgiri, 2003 [17], 1980–1997, Scotland	69/618	89.0	88.8	**—**	**—**	**—**	**—**
Bilateral renal agenesis	Schneuer, 2019 [42], 2004–2009, NSW, Australia	5/5	0.0	**—**	**—**	**—**	**—**	**—**
Cystic kidney disease		9/83	89.2 (82.5–95.8)	89.2 (82.5–95.8)	**—**	**—**	**—**	**—**
ICD-10 Q60-Q64	Tennant, 2010 [15], 1985–2003, Northern England	84/1,258	93.9 (92.4–95.1)	93.5 (86.6–94.2)	93.4 (91.9–94.6)	93.2 (91.6–94.5)	93.2 (91.6–94.5)	**—**
Bilateral renal agenesis		21/21	0.0	**—**	**—**	**—**	**—**	**—**
Cystic kidney disease		20/225	92.0 (87.6–94.9)	91.1 (86.6–94.2)	91.1 (86.6–94.2)	91.1 (86.6–94.2)	91.1 (86.6–94.2)	**—**
Renal agenesis or dysgenesis—ICD-9 753.0	Wang, 2011 [40], 1983–2006, USA	693/1,946	66.1 (63.9–68.1)	64.8 (62.6–66.9)	**—**	64.2 (62.0–66.3)	**—**	63.8 (61.6–66.0)
Down syndrome								
759.3 (ICD-8), 758.0 (ICD-9) and Q90.0, Q90.1, Q90.2 or Q90.9 (ICD-10)	Brodwall, 2018 [22], 1994–2009, Norway	78/1,251	96.3	94.2	**—**	**—**	**—**	**—**
	1994–1999		94.2^b^	91.8^e^	**—**	**—**	**—**	**—**
	2000–2009		97.5^b^	95.8^e^	**—**	**—**	**—**	**—**
758.0 (ICD-9)	Chua, 2020 [71], 1995–2014, Hong Kong	83/1,010	94.4 *(92*.*7–95*.*7)*	*91*.*8*^b^ *(89*.*9–93*.*4)*	**—**	**—**	**—**	**—**
	Dastgiri, 2003 [17], 1980–1997, Scotland	33/210	87.1 (82.6–91.7)^b^	84.3 (78.3–90.3)^b^	**—**	**—**	**—**	**—**
	Frid, 1999 [65], 1973–1980, Sweden	54/213	85.4 (*79*.*8–89*.*8*)	***77*.*4***	76.5 (*70*.*1–81*.*9*)	74.6 (*68*.*2–80*.*2*)^i^		
	Glasson, 2016 [66], 1953–2010, Western Australia	245/1,378	**—**	88 (86–90)	87 (85–89)	**—**	**—**	83 (80–85) at 30 years
	1980–2010	78/772						
	1980–1990		93 (89–96)	86 (81–89)	85 (80–89)		84 (79–88)	82 (77–87)
	1991–2000		97 (94–99)	96 (92–98)	95 (91–97)		94 (90–96)	94 (90–96)
	2001–2010		96 (92–98)	94 (90–96)	94 (90–96)	94 (90–96)	94 (90–96)	94 (90–96)
	Halliday, 2009 [67], Australia							
	1988–1990	25/236	*94*.*1*	89.4	**—**	**—**	**—**	**—**
	1998–2000	10/165	*94*.*5*	93.9	**—**	**—**	**—**	**—**
	Hayes, 1997 [68], 1980–1989, Ireland	63/389	88.2 (85–91)	***83 (79–87)***	83 (79–87)	**—**	**—**	**—**
	1980–1984		87	82	**—**	**—**	**—**	**—**
	1985–1989		90	86	**—**	**—**	**—**	**—**
BPA codes, or both BPA and ICD-9-CM, or ICD9-CM only (North Carolina and Colorado)	Kucik, 2013 [19], 1983–2003 (20-year survival), USA	1,584/16,506	92.9 (92.5–93.2)	91.0 (90.5–91.4)	90.7 (90.2–91.1)	**—**	88.1 (87.0–89.0)	**—**
	1983–1989 (20-year survival)	334/2,454	91.3 (90.0–92.4)	88.1 (86.8–89.3)	87.4 (86.0–88.6)	**—**	85.7 (84.1–87.1)	**—**
	1990–1996 (10-year survival)	624/5,441	91.2 (90.5–92.0)	89.2 (88.3–90.0)	88.4 (87.6–89.3)	**—**	**—**	**—**
	1997–2003 (5-year survival)	608/8,611	94.3 (93.8–94.8)	92.5 (91.9–93.0)	**—**	**—**	**—**	**—**
	Leonard, 2000 [69], 1980–1996, Western Australia	/440	91.7 (88.7–94.0)	87.0 (83.0–89.0)	85.0 (81.0–89.0)	**—**	**—**	**—**
	1980–1985		89	80 (72–86)^e^	79	**—**	**—**	**—**
	1986–1990		92	86 (79–91)^e^	85	**—**	**—**	**—**
	1991–1996		94	93 (88–96)^e^	**—**	**—**	**—**	**—**
Q900–Q902	Rankin, 2012 [14], Northern England, 1985–1990	54/235	86.0 (80.8–89.8)	79.2 (73.4–83.8)	78.3 (72.5–83.0)	**—**	77.5 (71.6–82.3)	**—**
	1991–1996	36/193	83.9 (78.0–88.4)	82.4 (76.2–87.1)	81.9 (75.7–86.6)	**—**	80.6	**—**
	1997–2003	21/241	94.2 (90.4–96.5)	91.7 (87.4–94.6)	91.2 (86.8–94.2)	**—**	90.7	**—**
ICD-9-CM (758.000–758.090)	Rasmussen, 2006 [70], 1979–1998, USA	70/645	92.9 (90.9–94.9)	***89*.*9 (87*.*3–92*.*1)***	88.6 (85.0–92.2)	**—**	87.4 (84.3–90.5)	**—**
	Schneuer, 2019 [42], 2004–2009, NSW, Australia	30/425	94.1 (91.9–96.4)	92.9 (90.5–95.4)	**—**	**—**	**—**	**—**
ICD-9 758.0	Wang, 2011 [40], 1983–2006, USA	754/6,819	92.0 (91.3–92.6)	89.9 (89.1–90.6)	**—**	88.9 (88.1–89.7)	**—**	87.5 (86.5–88.5)
	Wang, 2015 [41], 1999–2007, USA	944/15,939	94.1 (93.7–94.4)	**—**	92.8 (92.3–93.2)^d^	**—**	**—**	**—**
Trisomy 13								
	Meyer, 2016 [72], 1999–2007, USA	625/693	11.5 (9.3–14.1)	9.7 (7.2–12.5)	**—**	**—**	**—**	**—**
ICD-9, 758.1 or ICD-10, Q91.4–Q91.7	Nelson, 2016 [25], 1991–2012, Canada	/174	19.8 (14.2–26.1)	15 (10–21)	12.9 (8.4–18.5)	**—**	**—**	**—**
	Tennant, 2010 [15], 1985–2003, Northern England	26/29	13.8 (4.4–28.6)	**—** ^o^	**—**	**—**	**—**	**—**
ICD-9 758.1	Wang, 2011 [40], 1983–2006, USA	437/525	21.3 (17.9–24.9)	18.4 (15.3–21.9)	**—**	16.2 (13.0–19.7)		15.2 (12.0–18.8)
Trisomy 18								
	Meyer, 2016 [72], 1999–2007, USA	984/1,113	13.4 (11.5–15.5)	12.3 (10.1–14.8)	**—**	**—**	**—**	**—**
ICD-9, 758.2 or ICD-10, Q91.0-Q91.3	Nelson, 2016 [25], 1991–2012, Canada	/254	12.6 (8.9–17.1)	11 (8–16)	9.8 (6.4–14.0)			
	Schneuer, 2019 [42], 2004–2009, NSW, Australia	28/34	20.6 (7.0–34.2)	17.6 (4.8–30.5)	**—**	**—**	**—**	**—**
	Tennant, 2010 [15], 1985–2003, Northern England	62/63	1.6 (0.1–7.5)	**—** ^o^	**—**	**—**	**—**	**—**
ICD-9 758.2	Wang, 2011 [40], 1983–2006, USA	667/773	18.8 (16.1–21.6)	15.2 (12.8–17.8)	**—**	13.2 (10.9–15.8)	**—**	12.3 (9.8–15.1)
Skeletal dysplasia								
Osteogenesis imperfecta ICD-10 Q78.0	Folkestad, 2016 [13], 1977–2012, Denmark	24/366 (up to 20 years)	*94*.*8 (91*.*8*–*96*.*8)*	*94*.*8 (91*.*8*–*96*.*8)*	**—**	**—**	*91*.*6 (88*.*2*–*94*.*2)*	**—**
Skeletal dysplasia	Schneuer, 2019 [42], 2004–2009, NSW, Australia	15/75	80.0 (70.9–89.1)	80.0 (70.9–89.1)	**—**	**—**	**—**	**—**
Achondroplasia BPA code 756.430	Simmons, 2014 [20], 1996–2005, USA	4/106	*96*.*2 (90*.*1*–*98*.*8)*	*96*.*2 (90*.*1*–*98*.*8)*^k^	**—**	**—**	**—**	**—**
Achondroplasia/Hypochondroplasia	Tennant, 2010 [15], 1983–2003, Northern England	2/22	95.5 (71.9–99.4)	90.9 (68.3–97.7)	90.9 (68.3–97.7)	90.9 (68.3–97.7)	**—**	**—**
Prader-Willi syndrome								
	Lionti, 2012 [73], 1950–2010, Australia	15/163 (to 35 years)	***98*.*6 (95*.*2***–***99*.*7)***	***98*.*6 (95*.*2***–***99*.*7)***	97 (93–99)	***96*.*3 (91*.*1***–***98*.*4)***	94 (88–97)	***89*.*4 (80*.*8***–***94*.*5)***
ICD-10 Q87.1	Tennant, 2010 [15], 1983–2003, Northern England	1/10	100.0	90.0 (47.3–98.5)	90.0 (47.3–98.5)	**—**	**—**	**—**

Congenital anomaly subtypes were presented within the major congenital anomaly groups according to the European Surveillance of Congenital Anomalies (EUROCAT) classification [26].

Estimates (or 95% CI) in italics were not reported in the article but were estimated from the raw data provided and in italics, and bold values were extracted from Kaplan-Meier or actuarial survival curves. For calculation of 95% CIs, we used the efficient-score method (corrected for continuity) described by Newcombe, 1998 [29], based on the procedure outlined by Wilson, 1927 [30].

^a^18-year survival values.

^b^Provided by authors on request or confirmed by authors.

^c^Survival at ≥5 years reported.

^d^8-year survival values.

^e^*p*-Values < 0.05.

^f^Overall survival reported, including all deaths (also without operation or liver transplantation), without specifying age at survival.

^g^Deaths and secondary liver transplantation used in calculation of NLS.

^h^4-year survival values.

^i^14.5-year survival values.

^j^3-year survival values.

^k^2-year survival values.

^l^13-year survival values.

^m^Overall survival (beyond 1 year of age) for all live births reported.

^n^This article (Rankin, 2012 [14]) was included despite being a subset of the larger study analysing all types of congenital anomalies (Tennant and colleagues [15]) because it reported survival by year period and explored predictors of survival. To avoid duplication in reporting, survival for Down syndrome from Tennant and colleagues [15] was included in neither the tables of this review nor the meta-analysis.

^o^Survival not reported as <5 cases at risk at the end of the time period.

### Congenital anomalies of the nervous system

Survival in live births with anencephaly analysed by four studies was extremely low and varied from 0% [15,42] to 7.3% [40] by year 1 (Table 2).

**Fig 2:**
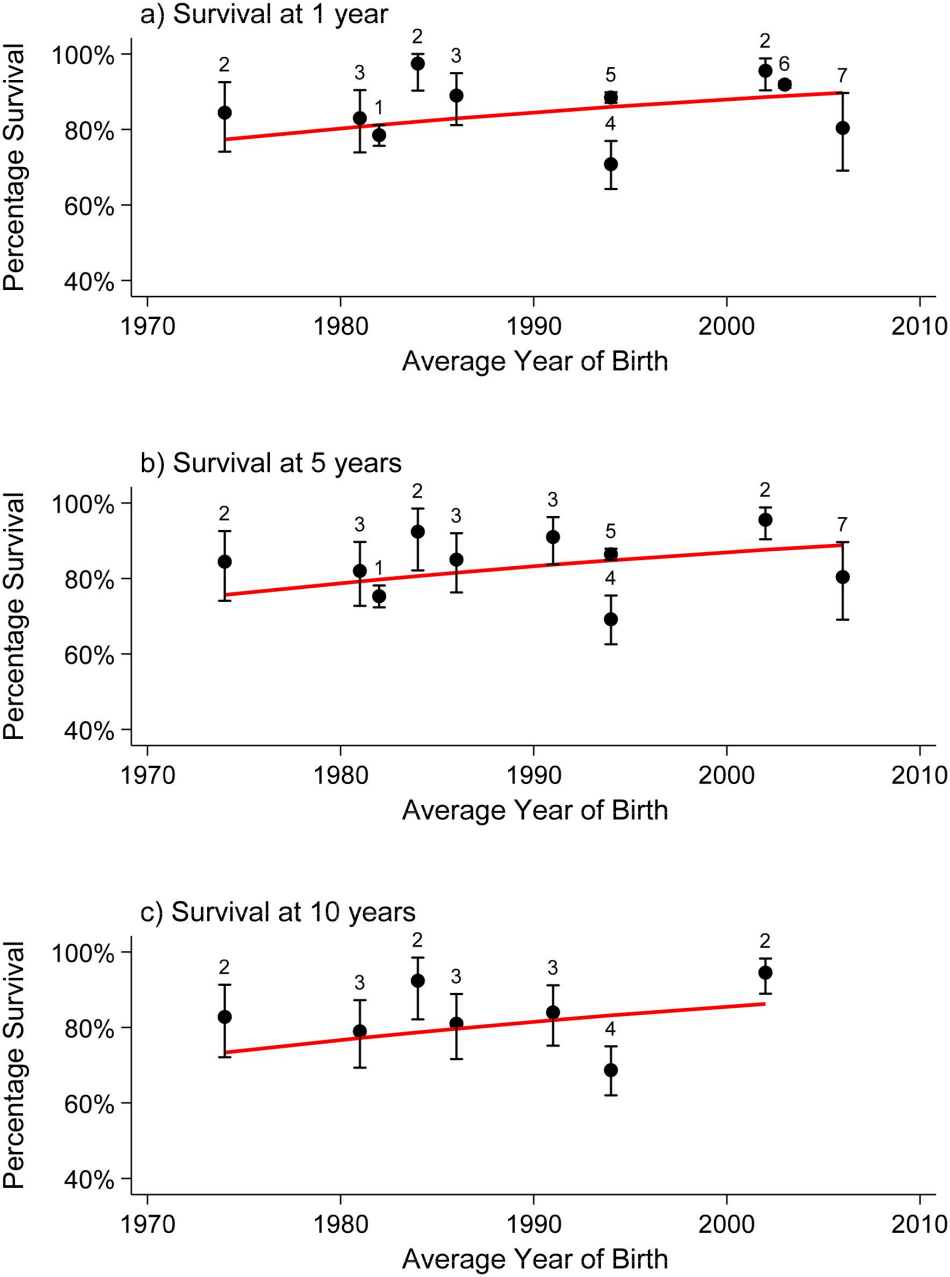
Survival estimates (with 95% confidence intervals) of children with spina bifida at 1 (a), 5 (b), and 10 (c) years of age over time (10 birth cohorts from 7 studies). The numbers at survival points indicate the included study, which may appear more than once if survival was reported for more than one birth cohort: 1 –Agha, 2006, Canada; 2 –Borgstedt-Bakke, 2017, western Denmark; 3 –Wong, 2001, Atlanta, USA; 4 –Tennant, 2010, Northern England; 5 –Wang, 2011; USA, 6 –Wang, 2015, USA; 7 –Schneuer, 2019, New South Wales, Australia.

Seven studies of survival in children born with spina bifida [6,15,40–42,45,48] including 7,422 live births were summarised in a meta-analysis, with pooled survival estimates of 92%, 91%, 89%, and 88% at ages 5, 10, 20, and 25 years predicted for children born in 2020 (Table 3). Survival has improved significantly over time, with an increased OR per 10-year increase in birth year 1.34 (95% CI 1.24–1.46, *p* < 0.001) (Table 3 and Fig 2).

**Table 3:** Predicted survival estimates for children born with selected congenital anomalies in 2000 and 2020 (results of the meta-analysis).

Congenital anomaly subtype (*n* of studies)	Survival period	Survival estimates for infants born in 2000, %	Survival estimates for infants born in 2020, %	Trend in survival over time	
				Relative odds (95% confidence interval)	*p*-Value
Spina bifida (*n* = 7)				1.34 (1.24–1.46)*	<0.001
	1 year	88 (87–89)	93 (91–94)		
	5 years	87 (86–88)	92 (90–94)		
	10 years	86 (84–87)	91 (89–93)		
	20 years	82 (80–85)	89 (86–92)		
	25 years	81 (77–83)	88 (84–91)		
Encephalocele (*n* = 4)				0.98 (0.95–1.01)*	0.19
	1 year	73 (73–74)	73 (71–74)		
	5 years	73 (73–74)	72 (71–74)		
	10 years	73 (72–74)	72 (70–74)		
	20 years	72 (71–73)	71 (69–74)		
	25 years	72 (71–73)	71 (68–74)		
Oesophageal atresia (*n* = 7)				1.50 (1.38–1.62)*	<0.001
	1 year	86 (85–87)	93 (92–94)		
	5 years	86 (85–87)	93 (91–94)		
	10 years	85 (84–87)	93 (91–94)		
	20 years	85 (82–87)	92 (90–94)		
	25 years	84 (82–87)	92 (89–94)		
Biliary atresia (*n* = 14)					
Overall survival				1.62 (1.28–2.05)*	<0.001
	1 year	87 (85–90)	95 (90–97)		
	5 years	85 (81–89)	94 (87–97)		
	10 years	82 (74–87)	92 (83–97)		
	20 years	73 (59–84)	88 (70–96)		
Survival with native liver				0.96 (0.88–1.03)*	0.26
	1 year	44 (41–47)	41 (35–48)		
	5 years	43 (38–47)	41 (33–49)		
	10 years	42 (36–48)	40 (30–50)		
	20 years	40 (31–50)	38 (26–52)		
Congenital diaphragmatic hernia (*n* = 9)				1.57 (1.37–1.81)*	<0.001
	1 year	67 (66–69)	84 (78–88)		
	5 years	67 (65–69)	83 (78–88)		
	10 years	67 (64–69)	83 (77–88)		
	20 years	66 (63–69)	83 (76–88)		
	25 years	66 (62–69)	83 (75–88)		
Gastroschisis (*n* = 5)					
	1 year	90 (90–91)	94 (90–96)	1.24 (1.02–1.50)*	0.029
	5 years	90 (89–91)	93 (89–96)		
	10 years	89 (87–91)	93 (88–96)		
	20 years	88 (84–90)	92 (85–95)		
Down syndrome (*n* = 10)					
With congenital heart defect (CHD)				1.99 (1.67–2.37)*	< 0.001
	1 year	92 (91–93)	98 (97–99)		
	5 years	90 (88–92)	97 (95–99)		
	10 years	88 (84–92)	97 (93–98)		
	20 years	87 (76–93)	96 (90–99)		
Without CHD				1.17 (0.91–1.5)*	0.23
	1 year	97 (96–98)	98 (95–99)		
	5 years	96 (95–98)	97 (94–99)		
	10 years	96 (92–98)	97 (91–99)		
	20 years	95 (85–98)	96 (82–99)		
Trisomy 18 (*n* = 4)				Not tested	
	1 year	15 (14–17)			
	5 years	14 (12–16)			
	10 years	13 (11–16)			

*Per 10-year increase compared to any previous birth cohort.

Four studies [15,40,41,47] reported survival of 1,562 encephalocele live births, with pooled survival estimates of 72%, 72%, 71%, and 71% at ages 5, 10, 20, and 25 years predicted for infants born in 2020 (Table 3). A small decrease in survival was observed over time, which was not statistically significant (*p* = 0.19) but was included in the model predictions to be consistent with the models for other congenital anomalies and acknowledging that the power from analysing only 4 studies is very low (Table 3 and S1 Fig).

Survival in individuals with hydrocephalus was reported in four studies, with the three more recent studies reporting very similar survival rates at age 5 years [15,40,42] and at 15 years in two studies with longer follow-up. The earlier study (1967–1979) reported lower survival of 50.8% for male individuals by age 18 years [8] (Table 2). Comparison of survival between these studies is difficult owing to differences in the inclusion criteria.

### Orofacial clefts

Seven studies providing survival estimates for children born with orofacial clefts [6,15–17,40–42] included 32,492 live births. There was insufficient number of studies reporting data by specific cleft type that met criteria for a meta-analysis; therefore, the survival data are presented in Table 2. Generally, 1-year and long-term survival of children with isolated cleft lip is over 99% [15,16], about 96%–97% for isolated cleft palate [15,16] and much lower for non-isolated orofacial cleft types [40,41].

### Anomalies of the digestive system

**Fig 3:**
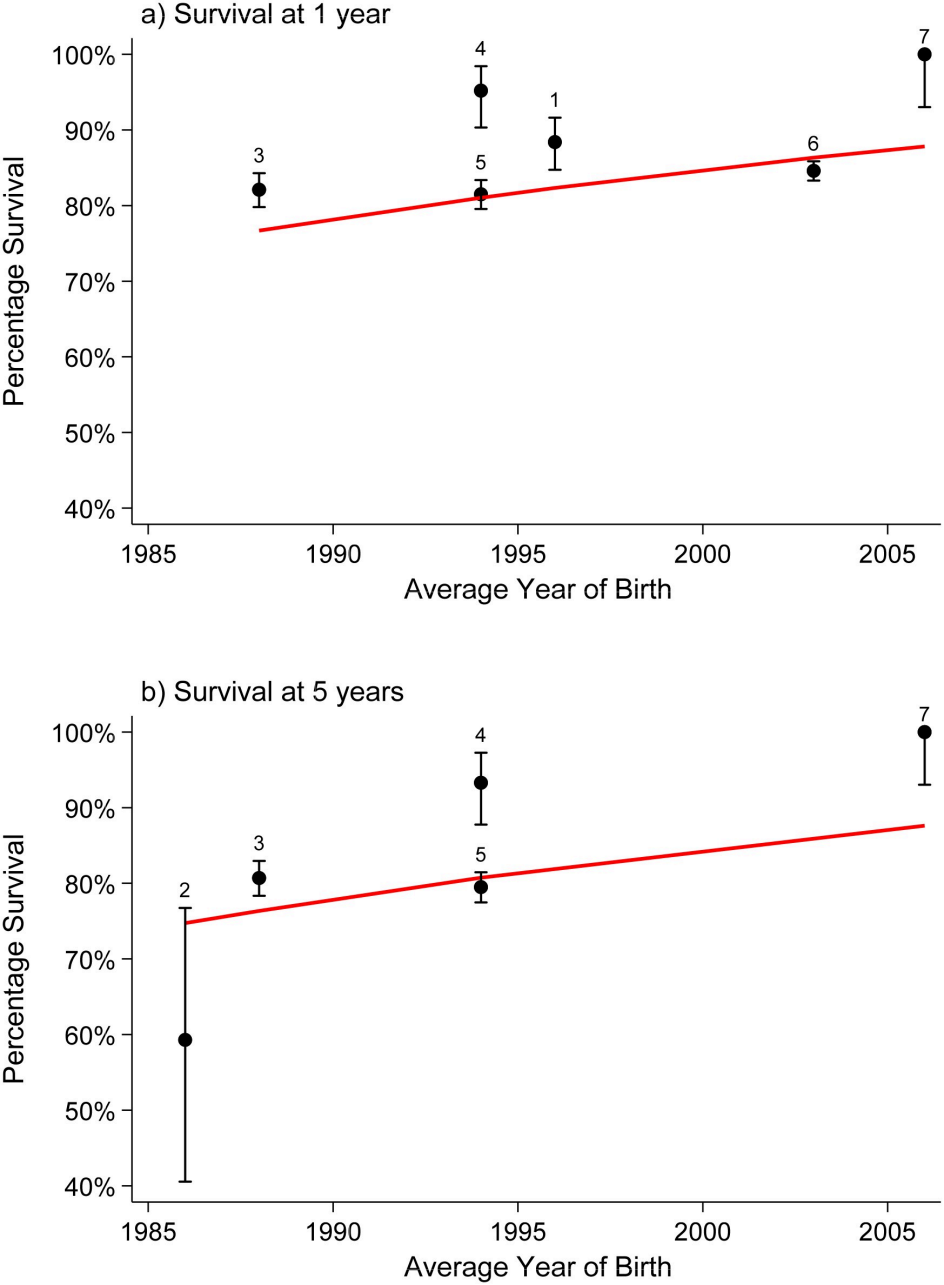
Survival estimates (with 95% confidence intervals) of children with oesophageal atresia at 1 (a) and 5 (b) years of age over time (7 studies). The numbers at survival points indicate the included study: 1 –Cassina, 2016, Northeast Italy; 2 –Garne, 2002, Funen, Denmark; 3 –Oddsberg, 2012, Sweden; 4 –Tennant, 2010, Northern England; 5 –Wang, 2011 USA; 6 –Wang, 2015, USA; 7 –Schneuer, 2019, New South Wales, Australia.

Seven studies reporting survival in children with oesophageal atresia (*n* = 6,303) were summarised in a meta-analysis [9,15,40–42,50,51]. There was a statistically significant improvement in survival over time, with an increased OR of 1.50 (95% CI 1.38–1.62, *p* < 0.001) per 10-year increase in birth year. The pooled survival estimates predicted for infants born in 2020 were 93%, 93%, 92%, and 92% at ages 5, 10, 20, and 25 years, respectively (Table 3 and Fig 3).

The survival estimates for children with anorectal malformations and for those with Hirschsprung disease were reported in four [15,40,41,52] and three studies [15,24,42] with survival ranging between 86% and 97% and between 93% and 98%, respectively (Table 2).

**Fig 4:**
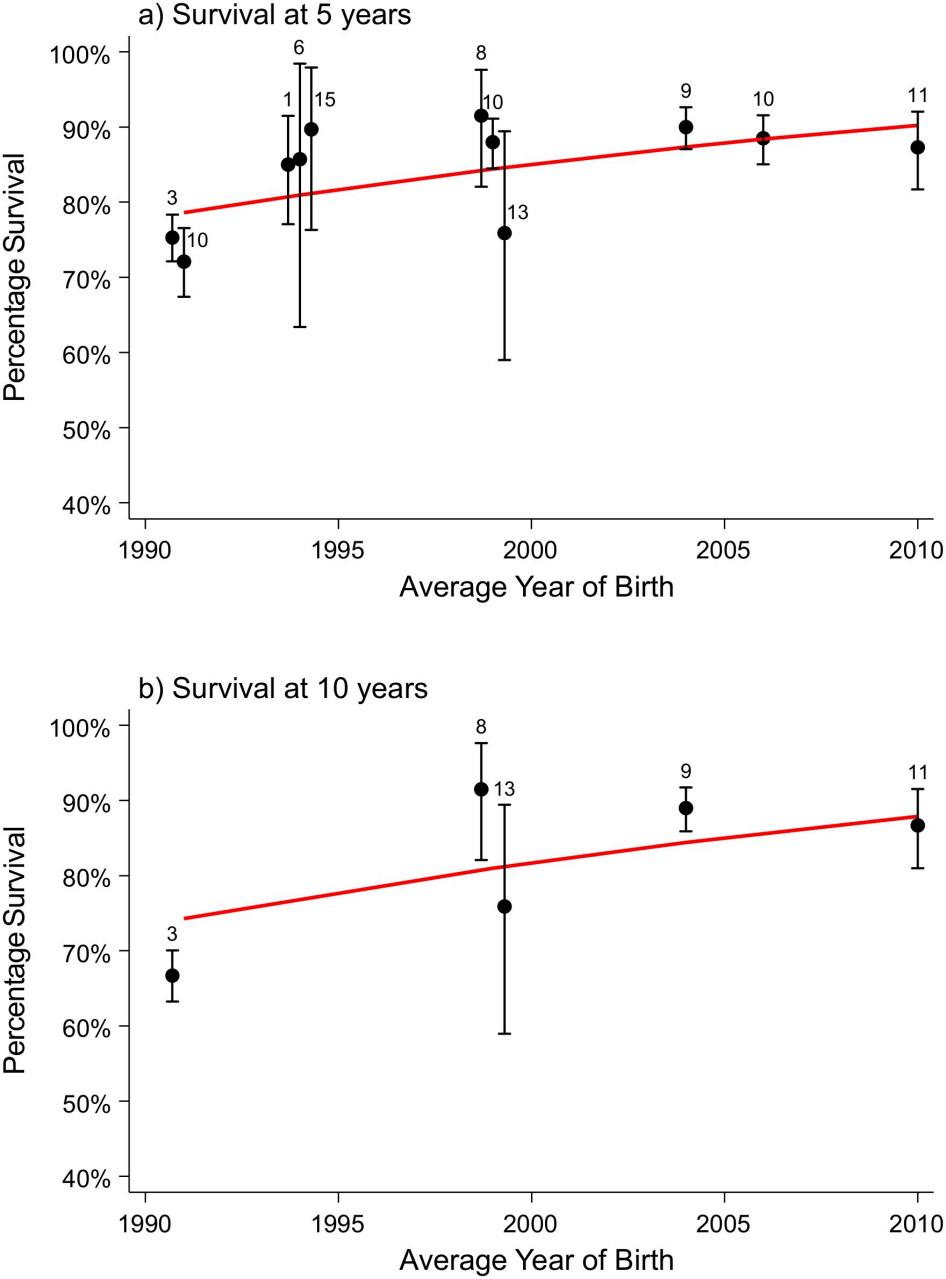
Survival estimates (with 95% confidence intervals) of children with biliary atresia at 5 (a) and 10 (b) years of age over time (11 birth cohorts from 9 studies). The numbers at survival points indicate the included study which may appear more than once if survival was reported for more than one birth cohort: 1 –McKiernan, 2000, UK and Ireland; 3 –Nio, 2003, Japan; 6 –Tennant, 2010, Northern England; 8 –Wildhaber, 2008, Switzerland; 9 –Davenport, 2011, England and Wales, 10 –Chardot, 2013, France; 11 –Pakarinen, 2018, Nordic countries; 13 –Grizelj, 2010, Croatia; 15 –Tu, 2015, South Australia.

Fourteen studies (*n* = 3,877 live births) reporting overall (after Kasai hepatoportoenterostomy [KP]) and/or liver transplantation) and/or survival with native liver (NLS, without liver transplantation) in children born with biliary atresia [15,36–38,55–64] were included in the meta-analysis. Pooled overall survival for biliary atresia at ages 5, 10, and 20 years were estimated to be 94%, 92%, and 88% for infants born in 2020 (Table 3). Fig 4 and Table 3 show a significant linear increasing trend in the overall survival and ORs for improvement in survival over time with OR = 1.62 (95% CI 1.28–2.05, *p* < 0.001). A small decrease in survival was observed over time in NLS, which was not statistically significant (*p* = 0.26) but was included in the model predictions to be consistent with the models for other congenital anomalies (Table 3). The predicted 5-year survival estimate was 41% (95% CI 33–49) for infants born in 2020 (the survival curve is shown in S2 Fig).

**Fig 5:**
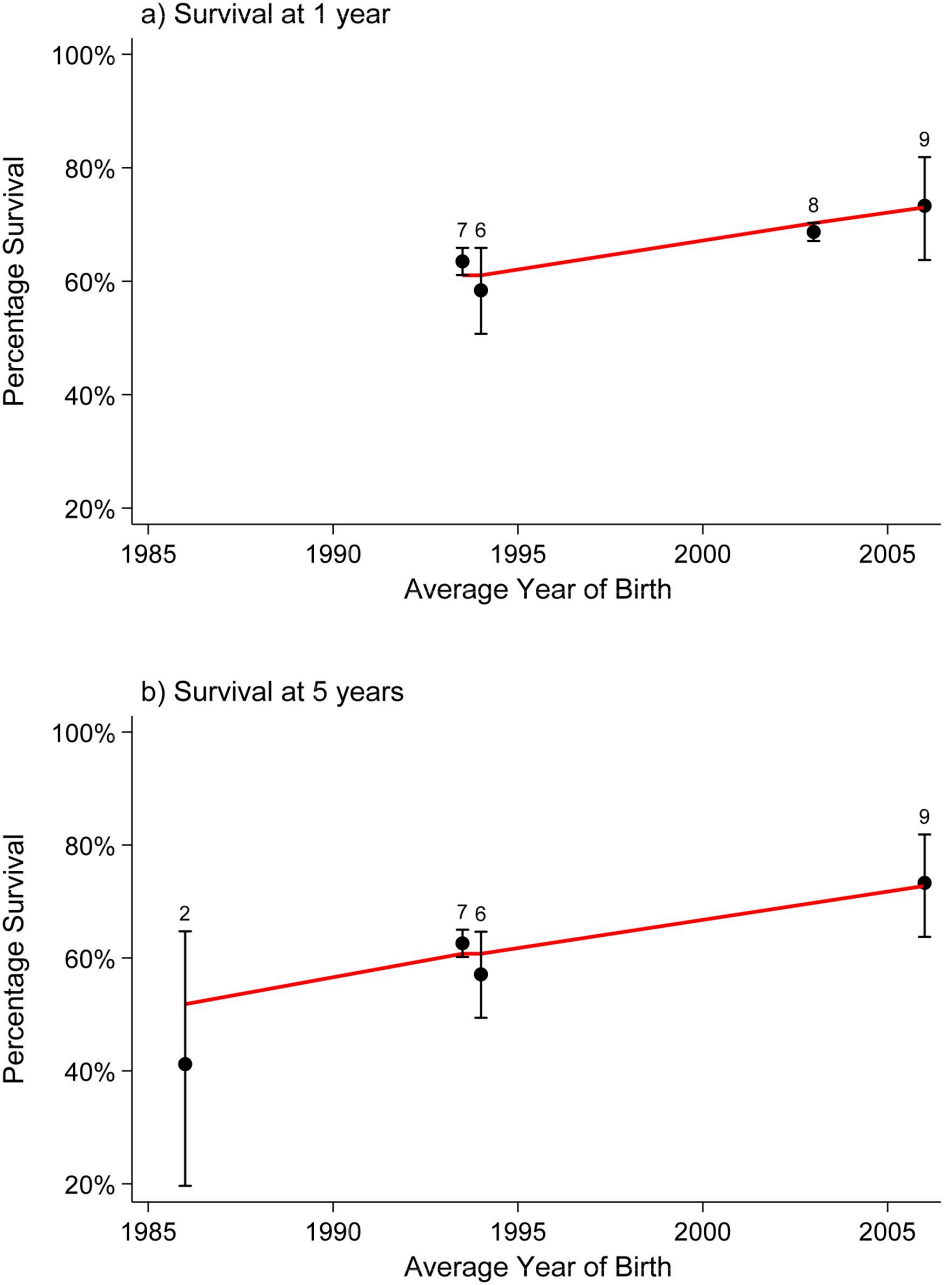
Survival estimates (with 95% confidence intervals) of children with congenital diaphragmatic hernia at 1 (a) and 5 (b) years of age over time (5 studies). The numbers at survival points indicate the included study: 2 –Garne, 2002, Denmark; 6 –Tennant, 2010, Northern England; 7 –Wang, 2011, USA; 8 –Wang, 2015, USA; 9 –Schneuer, 2019, New South Wales, Australia.

Nine studies of children born with CDH (*n* = 6,176) were summarised in a meta-analysis [15,18,23,40–42,51,53,54]; pooled survival estimates of 83% at ages 5, 10, 20, and 25 years respectively predicted for infants born in 2020 were reported in Table 3. The studies demonstrated that the majority of deaths occurred within the first year of life, with survival plateauing after that. Survival has improved significantly over time, with an increased OR per 10-year increase in birth year of 1.57 (95% CI 1.37–1.81, *p* < 0.001) (Table 3 and Fig 5).

### Abdominal wall defects

**Fig 6:**
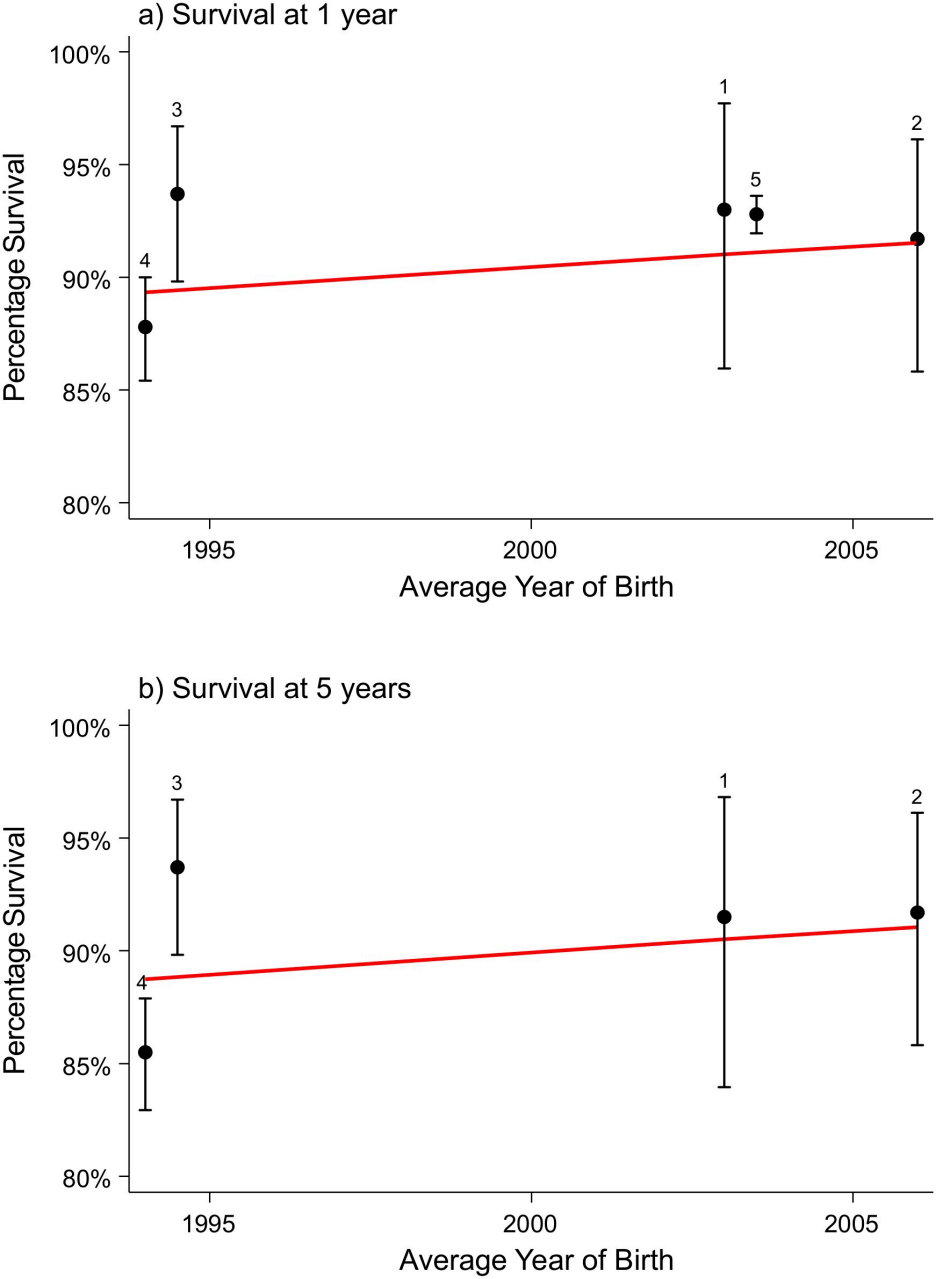
Survival estimates (with 95% confidence intervals) of children with gastroschisis at 1 (a) and 5 (b) years of age over time (5 studies). The numbers at survival points indicate the included study: 1—Risby, 2017, southern Denmark; 2—Schneuer, 2019, New South Wales, Australia; 3—Tennant, 2010, Northern England; 4—Wang, 2011, USA; 5—Wang, 2015, USA.

Five studies (*n* = 4,845) reporting survival of children born with gastroschisis were summarised in a meta-analysis [15,21,40–42]. There was a statistically significant improvement in survival over time, with an increased OR of 1.24 (95% CI 1.02–1.50, *p* = 0.029) per 10-year increase in birth year. Similar to studies on CDH, the majority of deaths occurred within the first year of life, with survival plateauing after that. The pooled survival estimates predicted for children born in 2020 were 94%, 93%, and 92% at ages 5, 10, and 20 years, respectively (Table 3 and Fig 6). Survival was consistently higher for gastroschisis than omphalocele in the three register-based studies reporting survival for both conditions [15,40,41] (Table 2).

### Chromosomal anomalies: Trisomies 21, 13, and 18

**Fig 7:**
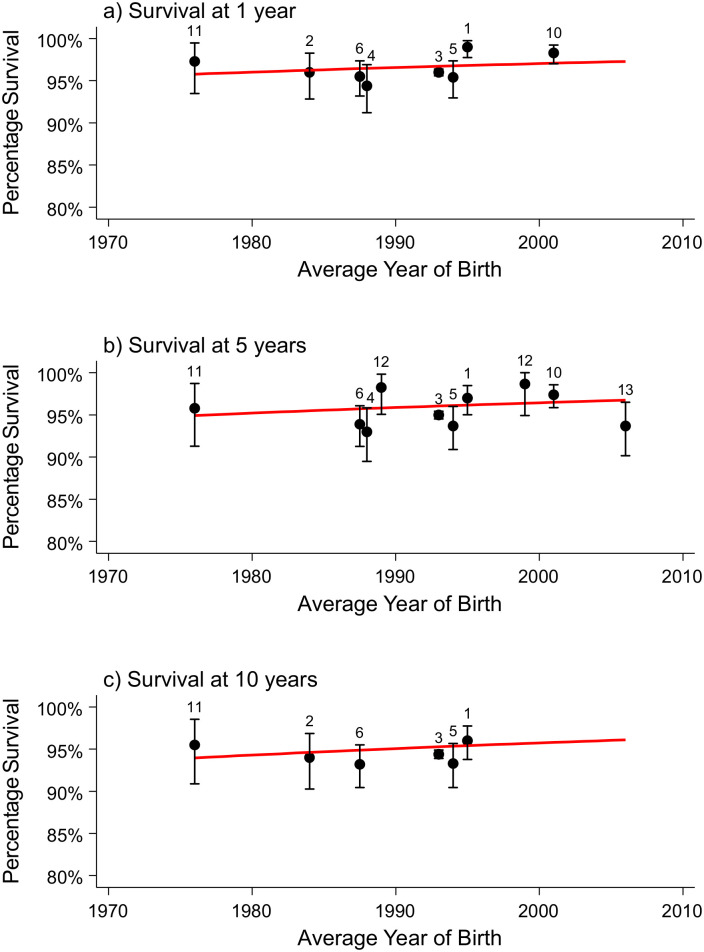
Survival estimates (with 95% confidence intervals) of children with Down syndrome associated with congenital heart defect at 1 (a), 5 (b), and 10 (c) years of age over time (11 birth cohorts from 10 studies). The numbers at survival points indicate the included study, which may appear more than once if survival was reported for more than one birth cohort: 1 –Glasson, 2016, Western Australia; 2 –Hayes, 1997, Ireland; 3 –Kucik, 2013, USA; 4 –Leonard, 2000, Western Australia; 5 –Rankin, 2012, Northern England; 6 –Rasmussen, 2006, Atlanta, USA; 10 –Brodwall, 2018, Norway; 11 –Frid, 1999, northern Sweden; 12 –Halliday, 2009, Victoria, Australia, 13 –Schneuer, 2019, New South Wales, Australia.

**Fig 8:**
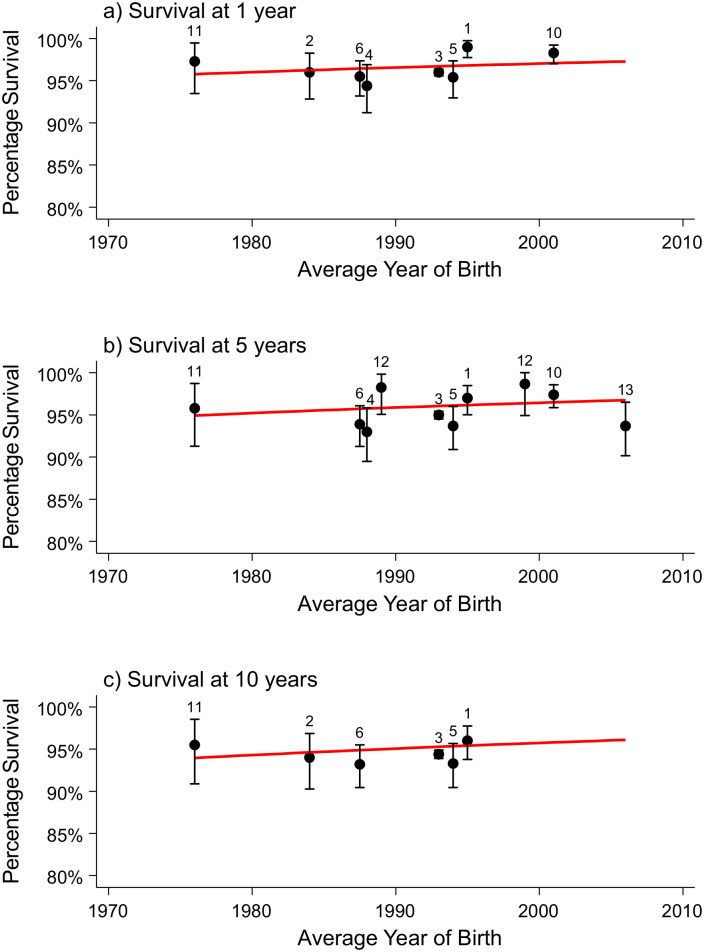
Survival estimates (with 95% confidence intervals) of children with Down syndrome without congenital heart defect at 1 (a), 5 (b), and 10 (c) years of age over time (11 birth cohorts from 10 studies). The numbers at survival points indicate the included study, which may appear more than once if survival was reported for more than one birth cohort: 1 –Glasson, 2016, Western Australia; 2 –Hayes, 1997, Ireland; 3 –Kucik, 2013, USA; 4 –Leonard, 2000, Western Australia; 5 –Rankin, 2012, Northern England; 6 –Rasmussen, 2006, Atlanta, USA; 10 –Brodwall, 2018, Norway; 11 –Frid, 1999, northern Sweden; 12 –Halliday, 2009, Victoria, Australia, 13 –Schneuer, 2019, New South Wales, Australia.

Survival of children born with Down syndrome (trisomy 21) reported by the presence of CHD in 10 studies (22,317 live births) [14,19,22,42,65–70] was summarised in the meta-analysis. We found significantly increasing survival trends over time for children with Down syndrome associated with CHD (OR = 1.99, 95% CI 1.67–2.37, *p* < 0.001) per 10-year increase in birth year; Table 3 and Fig 7). Children with Down syndrome without CHD had relatively high survival for live births in 2000 with no statistically significant improvement over time predicted for those born in 2020 (OR = 1.17, 95% CI 0.91–1.5, *p* = 0.23) (Table 3 and Fig 8). As there was a significant improvement in children with Down syndrome with CHD, the estimated improvement in children without CHD (although not statistically significant) was also modelled. For children born in 2020, pooled survival for Down syndrome at ages 5, 10, and 20 years were estimated to be 97%, 97%, and 96% for those both with and without CHD.

Studies analysing long-term survival in children with trisomies 13 (*n* = 4) and 18 (*n* = 5) reported consistently low 1-year survival ranging from 12% [72] to 21% [40] for trisomy 13 and from 2% [15] to 20.6% [42] for trisomy 18 (Table 2). However, large studies from the USA and Canada have shown that the majority of those individuals who survived to 1 year were alive at 5 [72], 10 [25], and 15 [40] years. A Canadian study reported that 76% and 65% of 1-year survivors with trisomy 13 were alive at 5 and 10 years, respectively; the corresponding figures for trisomy 18 were 90% and 77% [25]. In a USA study, conditional 5-year survival (for those who survived the first year of life) was over 80% for both trisomies 13 and 18 [72]. Four studies (*n* = 2,174) reporting survival of children born with trisomy 18 were summarised in a meta-analysis [25,40,42,72]. The pooled survival estimates predicted for children born in 2020 were 14% and 13% at ages 5 and 10 years, respectively (Table 3 and S3 Fig). The time trends were not tested, owing to a very small size of the most recent study reporting higher survival.

### Other congenital anomalies

Fewer studies analysing survival in children born with limb anomalies, renal anomalies, and skeletal dysplasias and syndromes met our inclusion criteria, with four being register-based studies that analysed a range of main anomaly groups/subtypes [15,40–42] (Table 2).

Survival of children born with upper or lower-limb defects was similar at about 87%–89% at 5 and 8 years of age in both USA register-based studies that included isolated anomalies and those with additional anomalies [40,41], whereas survival for upper-limb defects was higher at 99% than that for lower-limb defects at 93%% after 1 year of age in an English register-based study that included only isolated anomalies [15]. However, the latter study was much smaller, with ≤3 deaths for these anomalies.

Survival of children with urinary-system anomalies is not comparable between the studies, because of the differences in inclusion criteria (isolated versus non-isolated) and different birth year periods (Table 2).

Four studies reporting survival/mortality for children with skeletal dysplasia beyond 1 year of age were quite heterogeneous in terms of subtypes included, which may have caused differences in survival between a recent Australian study [42] and three other studies [13,15,20].

Two studies reported survival in patients with PWS, but the sample size was very low (*n* = 10, with one death) in one [15]. According to an Australian study using data from the PWS register, 10-year survival (97%) was similar to 1-year survival (98.6%); however, by age 25 it reduced to 89% [73].

### Factors associated with survival of children with congenital anomalies

Table 4 shows that overall, long-term survival in children born with congenital anomalies was much lower than in the reference populations, with the risks of death varying from 6.7 to 12.9 times greater than in the general population in the three studies reporting this [6–8]. In the USA study, the hazard ratio (HR) of death at age 7 years was only slightly reduced (from 7.2 to 6.9) when adjusted for child’s sex and mother’s race, age, and education [7] (Table 4). Table 4 also shows risks of death associated with some specific congenital anomalies compared to the reference population.

**Table 4:** Risk of death in children born with a congenital anomaly (CA) compared to the reference population.

Study	CA group/subtype	Length of survival for prediction analysis	Presence of CA	Unadjusted odds ratio (OR)/hazard ratio (HR)/relative risk (RR)/standardised mortality ratio (SMR) survival (95% CI)	Adjusted HR (aHR) (95% confidence interval [95% CI])	Factors adjusted for
Agha, 2006 [6]	All CAs	10 years for all, up to 17 years for birth year 1979	Yes	RR 12.9 (12.1–13.7)	**—**	**—**
Berger, 2003 [7]	All CAs	7 years	Yes	HR 7.2 (6.9–7.6)	aHR 6.9 (6.6–7.3)	Race, sex, mother’s age, mother’s education
Eide, 2006 [8]*	All CAs	18 years	Yes	RR 6.7 (6.3–7.1)	**—**	**—**
	Spina bifida	18 years	Yes	26.4 (21.9–31.8)	**—**	**—**
	Cleft lip	18 years	Yes	1.3 (0.6–2.8)	**—**	**—**
	Clef palate	18 years	Yes	3.2 (1.7–6.0)	**—**	**—**
	Cleft lip and palate	18 years	Yes	2.8 (1.8–4.4)	**—**	**—**
	Abdominal wall defect	18 years	Yes	18.6 (15.4–22.4)	**—**	**—**
	Multiple	18 years	Yes	24.0 (21.7–26.5)	**—**	**—**
Bell, 2016 [16]	Cleft lip only (isolated)	1 year	Yes	OR 0.56 (0.08–4.12)	**—**	**—**
	Cleft palate only (isolated)	1 year	Yes	OR 1.50 (0.45–4.96)	**—**	**—**
	Cleft lip and palate (isolated)	1 year	Yes	OR 1.37 (0.41–4.52)	**—**	**—**
Folkestad, 2016 [13]	Osteogenesis imperfecta	18 years	Yes	HR 66.1 (15.7–278.7)	aHR 68.1 (16.2–287.3)	Comorbidity
Löf Granström, 2017 [24]	Hirschsprung disease	50 years	Yes	HR 4.77 (2.87–7.91)	aHR 3.6 (2.04–6.37)	Down syndrome
Oddsberg, 2012 [9]	Oesophageal atresia	40 years	Yes	SMR 11.8 (10.3–13.5)	**—**	Matched with the background population by calendar year, sex, and age

*Selected anomalies only are presented.

Studies analysing survival predictors reported the presence of additional major anomalies as a universal risk factor of reduced survival [9,14,19,22,36,37,40,44,46,47,50,52,65,66,68,69,71,72] (Table 5), even after adjustment for such factors as birth cohort, birth weight, and/or gestational age at delivery [9,14,19,40,44,50,69,72] (Table 5). Other common risk factors associated with survival in children with congenital anomalies had a low birth weight (LBW) [9,14,19,40,47,48,50,52,66,69,71] or preterm birth [14,40,42,72] and earlier birth year period, after adjustment for covariates [9,14,19,40,50,66,69,71] (Table 5). Ethnicity was inconsistently associated with survival of children with some anomalies in USA studies. Hispanic ethnicity was associated with reduced survival by age 8 years in children with spina bifida weighing at birth between 1,500 and 2,499 g, but not in those with lower (<1,500 g) or higher (≥2,500 g) birth weight [46]. In another multistate USA study [41], there was no significant association of spina bifida survival at ≤8 years with any ethnic group when adjusted for covariates (Table 5, S6 Table). However, the latter study reported a significantly increased adjusted HR for reduced survival in Black and Hispanic children for both orofacial clefts and those with oesophageal atresia after adjustment for essential covariates and significantly increased adjusted HR for Down syndrome and CDH in Black children only [41] (S6 Table). Black ethnicity, however, was associated with a lower risk of death at 5 years for trisomy 18 [72]. In New York state, maternal nativity (‘Others’ versus ‘US born’) was significantly associated with a higher risk of death up to 25 years for all congenital anomalies and for anomalies of the central nervous system when adjusted for other factors including ethnicity [40]. Being aboriginal had a significant independent effect on reduced 10-year survival of children with Down syndrome in an earlier Australian study after adjustment for presence of CHD, birth weight, and birth cohort [69], but not in a more recent study [66] (Table 5).

**Table 5:** Predictors of survival/mortality in the included studies that explored factors associated with long-term survival at different age points beyond 1 year of life.

Study	Congenital anomaly (CA) group/subtype	Risk factor category	Unadjusted odds ratio (OR)/hazard ratio (HR)/ relative risk (RR)/survival rate (%) (95% confidence interval [95% CI])	Adjusted HR (aHR)/OR (aOR)/RR (95% CI)	Factors adjusted for
**Presence of additional anomalies (isolated versus non-isolated)**					
Agha, 2006 [6]	All CAs	Number of anomalies	—	10-year aRR	Gestational age (GA), birth weight (BW), maternal age, number of previous stillbirths
		1		1.0 (ref)	
		2		3.3 (3.1–3.7)	
		3		6.8 (6.2–7.6)	
		≥4		13.8 (12.7–15.0)	
Wang, 2011 [40]	All CAs*		—		Infant sex, BW, GA, plurality, number of CAs, parity, maternal ethnicity, nativity and education, birth year period
	
				25–year aHR	
		Isolated		1.0 (ref)	
		Non-isolated		2.8 (2.7–3.0)^c^	
Shin, 2012 [46]	Spina bifida		1-year survival	8-year aHR^a^	Ethnicity, birth cohort
				1500-2499g group:	
		Presence of major congenital heart defect (CHD)	81.9 (75.4–86.8)	2.6 (1.3–5.0)^c^	
				≥2500g: 3.6 (2.1–6.1)^c^	
		No	93.8 (92.6–94.7)^f^	1.0 (ref)	
Wong, 2001 [48]	Spina bifida		18-year survival		Maternal ethnicity, BW, location of the lesion
		Multiple defects	59.0 (42–84)	aHR not reported	
		No	81.9 (76–88)^d^	Not significant (NS) (results not reported) (yes versus no)	
Siffel, 2003 [47]	Encephalocele		20-year HR	20-year aHR	BW, race, birth cohort, GA
		Isolated	1.0 (ref)	1.0 (ref)	
		Non-isolated	3.8 (1.7–8.6)^e^	2.8 (1.2–6.7)^c^	
Cassina, 2016 [50]	Oesophageal atresia		25-year survival	25-year aHR	Birth period, BW
		Isolated	91.8 (86.9–96.7^c^	1.0 (ref)	
		Non–isolated	79.2 (72.9–85.5)	2.8 (1.3–6.0)^d^	
Oddsberg, 2012 [9]	Oesophageal atresia		40-year HR	40-year aHR	Sex, BW, birth year period
		Any CA	4.7 (3.5–6.3)	4.9 (3.7–6.6)	
		Circulatory CA	5.4 (3.9–7.5)	5.6 (4.0–7.8)	
		Noncirculatory CA	4.2 (3.0–5.8)	4.5 (3.2–6.2)	
		None	1.0 (ref)	1.0 (ref)	
Cassina, 2019 [52]	Anorectal malformations		HR	—	—
		≥2 associated CAs	7.9 (2.2–27.8)^d^		
		No	1.0 (ref)		
Chardot, 2013 [36]	Biliary atresia (BA)		20-year native liver survival (NLS) (%)	RR for 20-year NLS	Anatomical type, age at Kasai operation
		BA splenic malformation syndrome (BASM)	15.1 (SE=4.6)	1.0 (ref)	
		No	31.2 (SE=2.3)^f^	0.59 (0.45–0.78)^e^	
Hinton, 2017 [18]	Congenital diaphragmatic hernia (CDH)		20-year HR	20-year aHR	Treatment era, neighbourhood poverty
		Non-isolated	2.08 (1.24–3.48)	2.06 (1.22–3.49)	
		Isolated	1.0 (ref)	1.0 (ref)	
Brodwall, 2018 [22]	Down syndrome		—	5-year aHR	Year of birth
		Down syndrome (no additional CAs)		1.0 (ref)	
		Extracardiac malformation (ECM), CHD or a combination		Ranging from 2.6 (0.6–12) for ECM to 28 (8.9–88) for conotruncal CHD and ECM	
Chua, 2020 [71]	Down syndrome		—	5-year aHR	Age and sex
		CHD present		1.9 (1.2–3.0)^c^	
		No		1.0 (ref)	
Glasson, 2016 [66]	Down syndrome		25-year HR	25-year aHR	Sex, aboriginality, birth cohort
		CHD present	2.9 (1.7–4.9)^e^	3.1 (1.8–5.3)^e^	
		No	1.0 (ref)	1.0 (ref)	
Hayes, 1997 [68]	Down syndrome		10-yr survival	RR	Leukaemia (only significant variables in the bivariate model, i.e CAVD and leukaemia were included in the in the Cox proportional hazards model)
		
		
		No	90%	1.0 (ref)	
		Complete atrio-ventricular defect (CAVD) present	58%	5.6 (3.2–9.7)^e^	
Kucik, 2013 [19]	Down syndrome		—	20-year aHR	Race/ethnicity, BW, maternal age and education, birth period, and region of birth
		CHD present		2.7 (2.4–3.0)^c^	
		No		1.0 (ref)	
Leonard, 2000 [69]	Down syndrome		10-year HR	10-year aHR	Aboriginality, BW, maternal age, sex, birth cohort
		CHD present	3.4 (2.0–5.9)	3.7 (2.1–6.7)^d^	
		No	1.0 (ref)	1.0 (ref)	
Rankin, 2012 [14]	Down syndrome			20-year aHR	Birth year, maternal age, GA, Index of Multiple Deprivation (IMD), karyotype, plurality, infant sex, BW
		None	1.0 (ref)	1.0 (ref)	
		CHD only	3.8 (2.4–6.0)^e^	5.0 (3.1–8.1)^e^	
		Digestive only	5.1 (2.1–12.4)	6.5 (2.6–16.1)^e^	
		CHD and digestive only	8.8 (3.3–18.0)^e^	7.8 (3.8–16.4)^e^	
		Other(s)	3.5 (1.2–10.0)^c^	5.1 (1.7–15.1)^d^	
Schneuer, 2019 [42]	Down syndrome		5-year survival	—	—
		None	93.7 (90.5–96.9)		
		CHD	92.0 (88.3–95.8)^g^		
Meyer, 2016 [72]	Trisomy 18		1-year survival	5-year aHR	GA, maternal ethnicity, plurality, sex, presence of omphalocele, State, geographical area
		CHD	5.7 (3.0–9.6)^g^	1.3 (1.1–1.6)^c^	
		No	15.0 (12.8–17.4)	1.0 (ref)	
			
		Omphalocele	3.2 (1.4–13.0)^c^	1.6 (1.1–2.3)^c^	Same confounders, except for presence of CHD instead of omphalocele
		No	13.8 (11.8–16.0)	1.0 (ref)	
**Birth year**					
Wang, 2011 [40]	All CAs*		—	25-year aHR	Infant sex, BW, GA, plurality, number of CAs, parity, maternal age, ethnicity, nativity and education.
		1982-1988		1.8 (1.6–1.9)^c^	
		1989-1994		1.5 (1.4–1.6)^c^	
		1995-2000		1.3 (1.2–1.4)^c^	
		2001-2006^a^		1.0 (ref)	
Borgstedt-Bakke, 2017 [45]	Myelomeningocele		HR (overall risk of death up to 25 years)	—	—
		Time trend 1990-2015 versus 1970-1979 and 1980-1989	0.7 (0.5–1.0), *p*=0.05		
Shin, 2012 [46]	Spina bifida	Birth year (1979-2003)	—	8-year aHR NS for any BW groups	Ethnicity, presence of CHD
Siffel, 2003 [47]	Encephalocele		20-year HR	20-year aHR	BW, race, GA, presence of associated CAs
		1989-98	0.5 (0.2–1.2)^g^	0.4 (0.2–1.0)	
		1979-88	1.0 (ref)	1.0 (ref)	
			
		1989-98		0.3 (0.01–0.9)^c^ (for <2500g);	
				NS for ≥2500)	
Cassina, 2016 [50]	Oesophageal atresia		10-year survival (non-isolated only)	25-year aHR	BW, presence of additional anomalies
		1997+	87.3 (81.2–93.4)^d^	1.0 (ref)	
		Before 1997	58.7 (44.4–73.0)	2.4 (1.3–4.8)^d^	
Oddsberg, 2012 [9]	Oesophageal atresia		—	aHR (risk of death up to 40 years)	Sex, additional anomalies, BW
		1964-69		4.6 (2.3–9.2)	
		1970-79		3.1 (2.0–4.7)	
		1980-89		2.1 (1.4–3.2)	
		1990-99		1.2 (0.8–1.8)	
		2000-2007		1.0 (ref)	
Cassina, 2019 [52]	Anorectal malformations	1990-1999	4.7 (1.8–11.8)^d^	—	—
		2000-2012	1.0 (ref)		
Löf Granström, 2017 [24]	Hirschsprung disease		50-year OR	—	—
		1964-80	1.0 (ref)		
		1981-2000	0.6 (0.1–4.2)^g^		
		2001-2013	0.4 (0.1–3.3)^g^		
Hinton, 2017 [18]	CDH		20-year HR	20-year aHR	Neighbourhood poverty, presence of additional CAs
		<1988^h^	1.9 (1.3–3.3)	2.1 (1.3–3.6)	
		≥1988	1.0 (ref)	1.0 (ref)	
Chua, 2020 [71]	Down syndrome		—	5-year aHR	Age and sex
		1995-1999		1.0 (ref)	
		2000-2004		0.4 (0.2–0.8)^c^	
		2005-2009		0.5 (0.3–1.0)^c^	
		2010-2014		0.5 (0.3–1.0)^g^	
Glasson, 2016 [66]	Down syndrome		25-year HR	25-year aHR	Sex, aboriginality, presence of a CHD
		1980-1990	2.9 (1.7–5.2)^e^	2.9 (1.6–5.2)^e^	
		1991-2000	0.9 (0.5–1.9)^g^	0.7 (0.4–1.5)^g^	
		2001-2010	1.0 (ref)	1.0 (ref)	
Kucik, 2013 [19]	Down syndrome		—	20-year aHR	Race/ethnicity, BW, maternal age and education, presence of a CHD and region of birth
		1983-1989		1.0 (ref)	
		1990-1996		0.6 (0.5–0.8)^c^	
		1997-2003		0.5 (0.4–0.7)^c^	
Leonard, 2000 [69]	Down syndrome		10-year HR	10-year aHR	Aboriginality, BW, presence of CHD, maternal age group, sex
		1991-96	0.4 (0.2–0.8)^d^	0.3 (0.2–0.7)^d^	
		1983-89	1.0 (ref)	1.0 (ref)	
Rankin, 2012 [14]	Down syndrome		20-year HR	20-year aHR	Presence of additional structural anomalies, GA, maternal age, BW, karyotype, IMD, plurality, infant sex
		Continuous (between 1985-2003)	0.93 (0.89–0.96)^e^	0.89 (0.85–0.92)^e^	
**Low BW (LBW) or small for GA (SGA)**					
Agha, 2006 [6]	All CAs		—	10-year aRR	GA, number of birth defects, maternal age, number of previous stillbirths
		≤2500g		2.2 (2.0–2.4)^c^	
		2501-3000g		1.0 (ref)	
		3001-4000g		0.6 (0.5–0.7)	
		>4000g		0.5 (0.4–0.6)	
Wang, 2011 [40]	All CAs*		—	25-year aHR	Infant sex, plurality, number of CAs, parity, maternal age, ethnicity, nativity and education, birth year period
		≥37, <1500		4.4 (3.7–5.2)^c^	
		≥37, 1500-2499		2.9 (2.7–3.1)^c^	
		≥37, 2500-3999		1.0 (ref)	
		≥37, ≥4000		0.7 (0.6–0.8)^g^	
Nembhard, 2010 [43]	All CAs		5-year HR	5-year aHR	Maternal age, maternal education, infant sex, border county, and number of birth defects
		Appropriate for GA	1.0 (ref)	1.0 (ref)	
		SGA	2.6 (2.4–2.8)^f^	2.1 (1.9–2.2)^f^	
		Large for GA	0.6 (0.5–0.7)^f^	0.6 (0.5–0.7)^f^	
Wong, 2001 [48]	Spina bifida		Survival at <18 years		Maternal ethnicity, location of the lesion, presence of multiple defects
		<1500	33.3 (15–74)		
		1500-2499	68.2 (53–88)		
		≥2500	82.8 (77–90)		
				18-year aHR	
		<2500		2.3 (1.1–4.9)^c^	
		≥2500		1.0 (ref)	
Siffel, 2003 [47]	Encephalocele		20-year HR	20-year aHR	Race, birth cohort, GA, presence of associated CAs
		<2500g	6.3 (2.7–14.4)^f^	5.2 (2.7–12.6)^f^	
		≥2500g	1.0 (ref)	1.0 (ref)	
Cassina, 2016 [50]	Oesophageal atresia		—	25-year aHR	Birth period, presence of additional anomalies
		<2500		3.7 (1.7–8.3)^d^	
		≥2500		1.0 (ref)	
Oddsberg, 2012 [9]	Oesophageal atresia		—	40-year aHR	Sex, additional anomalies, birth year period
		<1500		7.0 (4.9–10.1)^c^	
		≥1500		1.0 (ref)	
Cassina, 2019 [52]	Anorectal malformations	<2500g	6.4 (2.3–17.9)^e^	—	—
		≥2500g	1.0 (ref)		
Chua, 2020 [71]	Down syndrome		—	5-year aHR	Age and sex
		<2500g		2.4 (1.2–4.8)^c^	
		≥2500g		1.0 (ref)	
Glasson, 2016 [66]	Down syndrome		25-year HR	25-year aHR	Sex, birth cohort, aboriginality, presence of a CHD
		<2500	2.3 (1.4–3.7)^e^	1.8 (1.0–3.1)^c^	
		≥2500	1.0 (ref)	1.0 (ref)	
Kucik, 2013 [19]	Down syndrome		—	20-year aHR	Race/ethnicity, maternal age and education, presence of a CHD, birth period, and region of birth
		<1500		8.5 (7.3–9.8)^c^	
		1500-2499		1.8 (1.6–2.0)^c^	
		≥2500		1.0 (ref)	
Leonard, 2000 [69]	Down syndrome		10-year HR	10-year aHR	Aboriginality, presence of CHD, maternal age group, sex, birth cohort
		<2500	2.3 (1.4–4.0)^d^	2.2 (1.2–3.7)^d^	
		≥2500	1.0 (ref)	1.0 (ref)	
Rankin, 2012 [14]	Down syndrome		20-year HR	20-year aHR	Presence of additional structural anomalies, birth year, maternal age, GA, birth year, karyotype, IMD, plurality, infant sex
		Continuous BW z-score	0.88 (0.77–1.0)	0.81 (0.71–0.91)	
**GA**					
Agha, 2006 [6]	All CAs		—	10-year aRR	Number of birth defects, birthweight, maternal age, number of previous stillbirths
		≤37 weeks		1.1 (0.99–1.2)	
		38-40 weeks		1.0 (ref)	
		>40 weeks		1.2 (1.1–1.3)	
Nembhard, 2010 [43]	All CAs		5-year HR	5-year aHR	Maternal age, maternal education, infant sex, border county, and number of birth defects
		≥37 weeks	1.0 (ref)	1.0 (ref)	
		<37 weeks	3.0 (2.8–3.2)^f^	2.7 (2.5–2.9)^f^	
Schneuer, 2019 [42]	All CAs		5-year survival	—	—
		≥37 weeks	95.6 (95.3–96.3)		
		<37 weeks	79.4 (77.5–81.4)^e^		
Wang, 2011 [40]	All CAs*		—	25-year aHR	Infant sex, plurality, number of CAs, parity, maternal age, ethnicity, nativity and education, birth year period
		<37 w, <1500		4.9 (4.6–5.2)^c^	
		<37 w, 1500-2499		2.7 (2.6–2.9)^c^	
		<37w, 2500-3999		1.5 (1.4–1.6)^c^	
		≥37 w, 2500-3999		1.0 (ref)	
Siffel, 2003 [47]	Encephalocele		20-year HR	—	—
		<37 weeks	4.7 (2.1–10.5)^f^		
		≥37 weeks	1.0 (ref)		
Glasson, 2016 [66]	Down syndrome		25-year HR	25-year aHR	Sex, birth cohort, aboriginality, presence of a CHD
		<37 weeks	2.4 (1.5–3.7^e^	1.9 (1.1–3.3)^c^	
		≥37 weeks	1.0 (ref)	1.0 (ref)	
Rankin, 2012 [14]	Down syndrome		20-year HR	20-year aHR	Presence of additional structural anomalies, birth year, maternal age, BW, karyotype, IMD, plurality, infant sex
		Continuous (weeks)	0.80 (0.76–0.84)^e^	0.76 (0.72–0.80)^e^	
Meyer, 2016 [72]	Trisomy 18		1-year survival	5-year aHR	Sex, maternal ethnicity, plurality, presence of CHD, presence of omphalocele, State, geographical area
		<32 weeks	4.9 (2.5–8.4)^e^	2.7 (2.2–3.4)^c^	
		32-36 weeks	9.4 (6.3–13.2)	1.5 (1.2–1.8)^c^	
		≥37 weeks	17.2 (14.3–20.3)	1.0 (ref)	
Meyer, 2016 [72]	Trisomy 13		1-year survival	5-year aHR	Sex, maternal ethnicity, State, geographical area
		<32 weeks	6.6 (3.1–11.9)^e^	1.9 (1.5–2.5)^c^	
		32-36 weeks	8.1 (5.0–12.1)	1.3 (1.0–1.6)	
		≥37 weeks	15.2 (11.6–19.2)	1.0 (ref)	
**Ethnicity**					
Berger, 2003 [7]	All CAs		7-year HR	7-year aHR	BW, sex, mother’s age, mother’s education, number of organ systems affected
		White	1.0 (ref)	1.0 (ref)	
		Black	1.5 (1.4–1.6)^c^	1.0 (0.9–1.1)^g^	
Wang, 2011 [40]	All CAs	Maternal nativity	—	25- year aHR	Infant sex, BW, gestational age, plurality, number of CAs, parity, maternal age, ethnicity and education, birth year period
	
		US born		1.0 (ref)	
		Other		1.1 (1.03–1.15)^c^	
Nembhard, 2010 [43]	All CAs		5-year HR	5-year aHR	Maternal age, maternal education, infant sex, border county, and number of birth defects
		Non-Hispanic White (NHW)	1.0 (ref)	1.0 (ref)	
		Non-Hispanic Black (NHB)	1.3 (1.6–1.9)^f^	1.5 (1.4–1.7)^f^	
		Hispanic	1.4 (1.3–1.5)^c^	1.1 (1.01–1.2)^c^	
Wong, 2001 [48]	Spina bifida		Survival at <18 years	aHR not reported	BW, presence of multiple defects, location of the lesion o
		White	82.8 (76–90)		
		Black	67.1 (56–81)^c^	NS (Black versus White)	
		Other	87.5 (63–100)		
Wang, 2015 [41]	Spina bifida, encephalocele, limb deficiencies, gastroschisis, omphalocele		—	8-year aHR	BW and gestational age, maternal age, birth period, and state surveillance program
		NHB		NS	
		Hispanic		NS	
		Asian/Pacific Islander (A/PI)		NS	
		American Indian/Alaska Native (AI/AN)		NS	
		NHW		1.0 (ref)	
	Cleft palate, cleft lip with/w/o cleft palate, esophageal atresia, rectal atresia/stenosis	NHB	—	*p* < 0.05	BW and gestational age, maternal age, birth period, and state surveillance program
		Hispanic		*p* < 0.05	
		A/PI		NS	
		AI/AN		NS	
		NHW		1.0 (ref)	
	CDH; Down syndrome	NHB	—	1.4^c^	BW and gestational age, maternal age, birth period, and state surveillance program
		Hispanic		NS	
		A/PI		NS	
		AI/AN		NS	
		NHW			
Shin, 2012 [46]	Spina bifida		1-year survival	8-year aHR	Birth year, presence of CHD
		White	94.1 (92.6–95.4)	1.0 (ref)	
		Black	87.8 (82.5–91.6)^c^	NS for any BW groups^g^	
		Hispanic	92.2 (90.3–93.8)	3.7 (1.8–7.8)^c^ for 1500-2499g group, NS for other BW groups	
Siffel, 2003 [47]	Encephalocele		20-year HR	20-year aHR	BW, birth cohort, gestational age, presence of associated CAs
		Black	2.7 (1.1–6.5)^c^	2.4 (0.95–5.9)^g^	
		Other	1.0 (ref)	1.0 (ref)	
Glasson, 2016 [66]	Down syndrome		25-year HR	25-year aHR	Sex, birth cohort, presence of a CHD
		Aboriginal	1.6 (0.7–3.8)^g^	1.1 (0.5–2.7)^g^	
		Non-aboriginal	1.0 (ref)	1.0 (ref)	
Leonard, 2000 [69]	Down syndrome		10-year HR	10-year aHR	Presence of CHD, BW, maternal age, sex, birth cohort
		Aboriginal	3.2 (1.4–7.4)^d^	3.2 (1.3–7.9)^d^	
		Non-aboriginal	1.0 (ref)	1.0 (ref)	
Kucik, 2013 [19]	Down syndrome		—	aHR (overall survival – up to 20 years)	BW, maternal age and education, presence of a CHD, birth period, and region of birth.
		White		1.0 (ref)	
		Black		1.4 (1.0–1.6)	
		Hispanic		0.8 (0.7–0.9)^c^	
		Other		1.3 (1.1–1.6)^c^	
Meyer, 2016 [72]	Trisomy 18		1-year survival	5-year aHR	Gestational age, plurality, sex, presence of CHD, presence of omphalocele, State, geographical area
		NHW	13.6 (10.7–16.9)	1.0 (ref)	
		NHB	17.3 (12.5–22.7)^c^	0.7 (0.6–0.9)^c^	
		Hispanic	10.1 (7.3–13.5)	0.9 (0.8–1.1)	
		NH A/PI	13.2 (4.8–25.8)	0.8 (0.5–1.2)	
		Other/unknown	23.3 (10.3–39.4)	1.0 (0.6–1.7)	
**Maternal age (years)**					
Agha, 2006 [6]	All CAs		—	10-year aRR	Number of birth defects, gestational age, birthweight, number of previous stillbirths
		≤20		1.2 (1.03–1.3)^c^	
		21-34		1.0 (ref)	
		≥35		0.9 (0.8–1.1)	
Wang, 2011 [40]	All CAs		—	25-year aHR	Infant sex, BW, gestational age, plurality, number of CAs, parity, maternal ethnicity, nativity and education, birth year period
		≤19		1.2 (1.1–1.3)^c^	
		20-24		1.1 (1.03–1.2)^c^	
		25-29		1.05 (1.0–1.1)^g^	
		30-34		1.0 (ref)	
		≥35		1.0 (0.9–1.0)^g^	
Leonard, 2000 [69]	Down syndrome		10-year HR	10-year aHR	Aboriginality, presence of CHD, sex, birth cohort, BW
		<20	2.8 (1.1–7.1)^c^	2.4 (0.9–6.1)^g^	
		≥20	1.0 (ref)	1.0 (ref)	
Rankin, 2012 [14]	Down syndrome		20-year HR	20-year aHR	Presence of additional structural anomalies, birth year, BW, gestational age, karyotype, IMD, plurality, infant sex
		<20	1.25 (0.63–2.49)	0.67 (0.32–1.40)	
		20-30	1.0 (ref)	1.0 (ref)	
		>30	0.91 (0.61–1.36)^g^	1.08 (0.71–1.64)^g^	
**Centre annual caseload (BA studies)**					
Chardot, 2013 [36]	BA	1986-1996^c^	5-year overall survival	—	—
		≥20	77.6 (72.1–83.1)		
		3 to5	61.9 (51.1–72.7)		
		≤2	69.6 (62.5–76.7)		
		1997-2002	NS		
		2003-2009	NS		
Leonhardt, 2011 [61]	BA		2-yr NLS	—	—
		<5	7.7%		
		≥5	26.4%^d^		
McKiernan, 2000 [39]	BA		5-year RR	Caseload - the only significant factor, RR not reported	Age at surgery, sex, gestational age, presence of BASM
		<5	1.0 (ref)		
		>5	0.32 (0.11–0.94) (overall survival)		
		>5	0.48 (0.28–0.86) (NLS)		
McKiernan, 2009 [38]	BA		Overall 13-year survival (%)	—	—
		<5	75 (61.6–89.4)		
		>5	89.5 (81.3–97.7)^g^		
			13-year NLS (%)		
		<5	27.3 (12.3–42.3)		
		>5	54.0 (40.8–67.2)^d^		
Pakarinen, 2018 [58]	BA		5-year NLS	aHR for 5-year NLS	Presence of associated CAs, age at surgery, sex, anatomical type of BA, presence of BASM, clearance of jaundice, European ethnicity
		
		>3	66 (54–77)^d^	3.5 (1.8–6.8)^e^	
		<3	44 (32–56)	1.0 (ref)	
**Age at Kasai hepatoportoenterostomy for NLS (BA studies)**					
Chardot, 2013 [36]	BA		20-year survival (%)	RR for 20-year NLS	Anatomical type, presence of BASM
		≤30 days	38.9 ((SE=7.5)^d^	0.54 (0.37–0.79)^f^	
		31-60 days	31.7 (SE=3.4)	0.58 (0.45–0.75)	
		61-90 days	28.1 (SE=3.1)	0.74 (0.37–0.79)	
		>90 days	18.7 (SE=4.8)	1.0 (ref)	
Davenport, 2011 [37]	BA	<44 days	NS for 10-yr NLS	—	—
		44-55	Overall: *p*=0.34;		
		56-69	or between two most different (<44 and 44-55) groups: *p*=0.15		
		70+			
De Carvalho, 2010 [55]	BA		HR for 4-year NLS	—	—
		≤60 days	1.0 (ref)		
		61-90	1.6 (1.2–2.3)^d^		
		>90	1.9 (1.3–2.7)^d^		
De Vries, 2011 [56]	BA		20-year NLS survival (%)	—	—
		<45 days	14±9^g^ (versus 45-60 or 60-75 days)		
		45-60	33±8^g^ (versus 60-75)		
		60-75	42±10^c^ (versus >75)		
		>75	11±6		
Pakarinen, 2018 [58]	BA		5-year NLS	5-year aHR	Presence of associated CAs; sex; anatomical type of BA, presence of BASM, clearance of jaundice, European ethnicity, centre caseload
		< 65	66 (55–78)^d^	1.5 (0.8–2.9)^g^	
		>65	44 (32–56)	1.0 (ref)	
Schreiber, 2007 [63]	BA		4-year NLS	—	—
		≤30	49 (26–69)^f^		
		31-90	36 (28–43)		
		>90	23 (12–37)		
Wildhaber, 2008 [64]	BA		4-year NLS (% ± SE)	—	—
		≤45	75 ±15.3		
		46-75	33.3 ± 10.3		
		>75	11.3 ± 10.6		

Only factors examined in ≥3 studies are included, *n* = 33 studies.

*The association with the reported factors was also significant for the following CA groups: central nervous system, orofacial clefts, gastrointestinal, genitourinary, musculo-skeletal, and c^h^romosomal anomalies, but was not reported for specific CA subtypes.

^a^Only predictors with significant results in either unadjusted or adjusted analysis are shown.

^b^Conotruncal defects include Tetralogy of Fallot, double outlet right ventricle, conotruncal ventricular septal defects, aortic hypoplasia, truncus arteriosus, and interrupted aortic arch.

^c^*p*<0.05 (also for those significant associations for which the exact *p*-value not reported).

^d^*p*<0.01.

^e^*p*<0.001.

^f^*p*<0.0001.

^g^NS (*p*≥0.05).

^h^Treatment eras are before 1988 (routine immediate surgical repair) and post-1988 (preoperative stabilisation, delayed surgical repair, and addition of lung-sparing strategies).

Because of the rarity of biliary atresia and dependence of outcome on successful and timely KP, the survival factors most commonly explored in these children were annual centre caseload [36,38,39,58,61] and age at KP [36,37,55,56,58,63,64]. The higher centre caseload—i.e., care centralisation associated with centralisation of surgical and medical resources and better surgical staff experience—and earlier age at KP were considered as positive factors for survival. Earlier KP was associated with better NLS at age 4 years [55,63,64] and 5 years [58]. The 20-year NLS was also higher for children operated at a younger age compared to >90 days in a French study [36] and to >75 days in a Dutch study [56]. However, 10-year NLS was not associated with age at KP in a UK study [37]. Centre caseload (<5 versus >5) was the only significant factor for both 5-year overall survival and NLS in an earlier UK study after adjustment for confounders [39], but at 13 years it remained a significant factor for NLS only [38]. Centre caseload (<3 versus >3) was also a significant predictor of 5-year NLS in a collaborative Scandinavian study [58], and in Finland centralisation of care for patients with biliary atresia significantly increased both overall and NLS to age 4 years [60] (Table 5). In a French study, lower centre caseload was significantly associated with both reduced overall survival and NLS in the earlier period (1986–1996) but not in the later (1992–2002 and 2003–2009) periods [36] (Table 5).

## Discussion

This systematic review and meta-analysis summarise long-term survival for individuals born with a range of congenital anomalies from population-based studies, covering a total population of 367,801 live births with congenital anomalies. This work is part of the ‘EUROlinkCAT: Establishing a linked European Cohort of Children with Congenital Anomalies’, a collaborative project investigating survival, morbidity, and educational outcomes in children born with congenital anomalies using population-based data from multiple EUROCAT registries linked to a number of health and education datasets (https://www.eurolinkcat.eu/). A total of 55 studies were included in the narrative synthesis, with 41 studies included in meta-analyses. Our meta-analyses showed predicted 20-year survival for children born in 2020 as 89% for spina bifida (*n* = 7 studies), 71% for encephalocele (*n* = 4), 92% for oesophageal atresia (*n* = 7), 88% for biliary atresia (*n* = 14), 83% for CDH (*n* = 9), 92% for gastroschisis (*n* = 5), and 96% for Down syndrome both with and without CHD (*n* = 10). As expected, the first year of life was critical for survival of children with a congenital anomaly, but there remained a gradual decline in survival beyond infancy that exceeded that of the general population. Our meta-analyses showed statistically significant improvement in survival over time in those with spina bifida, oesophageal atresia, biliary atresia, CDH, gastroschisis, and Down syndrome in those with CHD, but not in those with encephalocele, biliary atresia with a native liver, or Down syndrome without CHD. The evidence from individual studies showed that improvement in survival was not equal for all patient groups, being more pronounced, for example, for a group with non-isolated anomalies [50] or differing by ethnic group [18]. The commonest significant independent predictors of reduced survival for any congenital anomaly type were presence of additional structural anomalies, LBW, and earlier birth year period.

Advances in prenatal diagnosis, neonatal care (including intensive care, standard use of antenatal steroids, and surfactant therapy for prevention of neonatal mortality and morbidity in preterm births), early surgical interventions, ECMO, care centralisation, and liver transplantation (for biliary atresia patients) were likely to improve survival in these children. One of the factors that may have contributed to the improvement in survival of live births with spina bifida over the last 30 years, reported by individual studies [45,46] and revealed by our meta-analysis, is the increasing use and accuracy of prenatal diagnosis and the consequent increase in terminations of pregnancy for fetal anomaly (TOPFAs) for most severe anomaly types. One of the included studies found an independent association of annual TOPFA rate with increase in survival [15]. Indeed, there is evidence of an association between the increased TOPFA rates and reduced live-birth prevalence of congenital anomalies and consequent reduction in infant mortality [74,75]. Periconceptional folic acid intake or fortification is likely to be another factor of improving survival by reducing the number of severe types of spina bifida [76]. Advances in neonatal and surgical care, including early neonatal or elective fetal surgery for spina bifida repair [77,78], may have also contributed to increased long-term survival of these patients.

In addition to the above listed general advances in prenatal diagnosis and neonatal care contributing to improvement in survival of children with various types of congenital anomalies, there are specific principles in care of CDH patients that affect survival of these patients. These are early intubation with avoidance of bag mask ventilation; prevention and treatment of pulmonary hypertension and lung hypoplasia, the primary causes of neonatal mortality in CDH patients, by minimising lung damage using gentle lung ventilation (e.g., high-frequency oscillatory ventilation); gastric decompression, ensuring adequate blood pressure; ECMO, if indicated; and delayed surgical repair after stabilisation of pulmonary and haemodynamic status [79].

Studies of survival of children with biliary atresia, a rare life-limiting progressive disorder of bile ducts, which is fatal without early surgery (KP) and eventually requires liver transplantation, were mostly limited to 4–5 years of follow-up, with two European studies reporting survival at age 20 years [36,56]. Despite a number of existing reviews on biliary atresia, including a systematic review published in 2013 [80], this condition was included in our review, as we aimed to update the existing evidence on a population base and pool data in a meta-analysis. The 4-year NLS was as low as 23.5% before centralisation of care (1987–2005) in Finland, increasing to 76% after centralisation [60]. In addition to centralised care, earlier age at KP was a predictor of better NLS in these patients in some studies [36,55,56,58,63,64], which was in agreement with an earlier systematic review [80]. However, in the UK centre, caseload was the only significant factor associated with better NLS at age 5, 10, or 14 years [37–39]. Care centralisation and liver transplantation are crucial factors in the care of these patients, increasing the overall 10-year patient survival to 79.7% in France [36] and 87%, 89%, and 91.5% in the Scandinavian countries [58], UK [37], and Switzerland [64], respectively.

A significant association between birth year and increase in survival of individuals with Down syndrome was reported in some reviewed studies [14,19,66,69]. Recent advances in intensive care of preterm and very LBW babies are likely to account for prevention of infant death in many children with Down syndrome who are at a 2-fold higher risk of infant death compared to very LBW babies without a congenital anomaly, owing to higher risk of infection and lung disease such as bronchopulmonary dysplasia [81]. Improved access to early cardiac surgery in infants with septal defects may have also contributed to their increased long-term survival by prevention of development of pulmonary arterial hypertension and Eisenmenger syndrome, the conditions of high-risk mortality [66,82,83]. Our meta-analysis has shown that survival estimates significantly increased over time for children with CHD, but the improvement for those without CHD was not statistically significant.

Until recently, trisomies 13 and 18 were regarded as lethal conditions, with the majority of prenatally diagnosed cases being electively terminated and those resulting in live births (about 19% and 14% for trisomies 13 and 18, respectively [84]) commonly receiving palliative care only. Two recent studies that analysed survival of children with trisomy 13 or 18 beyond 1 year [25,72] demonstrated that although cumulative survival was low, children who were alive at their first birthday had around an 80% chance of survival to their fifth birthday, and 86% of those who survived to age 5 were likely to live to age 10 years [25]. Despite the emerging evidence that intensive care and surgical interventions improve the survival in these children [85], the debate in the medical community in relation to the interventions to be offered to infants with these trisomies is ongoing [85–87] because of severe cognitive impairment in the survivors and considerations in relation to family and societal burden [87]. Current medical experts’ view is that medical care of children with trisomies 13 and 18 should be evidence-based [85], and more consideration should be given to personalised care of these children, providing more information to parents and taking into account their hopes and wishes [86].

The commonest significant independent predictors of reduced survival at and beyond 1 year of life for any congenital anomaly type were presence of additional structural anomalies, LBW, and earlier birth year period. The association with ethnicity was inconsistent in the USA studies across different anomaly types and aboriginality was significantly associated with reduced survival in children with Down syndrome in an earlier study [69] but not in a more recent one [66]. Ethnicity may be a proxy indicator of deprivation, which is associated with increased neonatal and infant mortality across all major causes of death including congenital anomalies [88–91]; however, the associations with other deprivation measures were not analysed in the included studies.

This systematic review and meta-analysis is strengthened by a rigorous search strategy and comprehensive literature searches using a combination of multiple sources of information to identify relevant papers. Our systematic search strategy was informed by the research protocol registered in the PROSPERO database and developed according to clear inclusion criteria based on elements of the PICOS framework. To ensure that the search strategy was appropriately inclusive, it was piloted using Medline, refined, and retested. Additionally, we manually searched the reference lists of all included papers, citations of the included papers repeating that process for newly identified papers, and also key journals in the field. This approach is recognised to increase the identification of relevant papers [92]. A 10% sample of titles and abstracts of records was screened by coauthors to enable consistency in study inclusion following predefined eligibility criteria. All data were extracted in duplicate by two independent reviewers to ensure accuracy in the reported results and to minimise subjectivity. Authors were contacted where more information was required during data extraction. We also used an established quality-assessment tool as part of the critical appraisal process.

We restricted the start year for our literature searches to 1995 to make the birth cohorts used in the studies more comparable in relation to antenatal and neonatal care and treatment availability/policies and to avoid subsequent differences. In addition, restricting our review to population-based studies with follow-up from birth reduces bias in death ascertainment.

We used multilevel meta-analytic models to allow for studies reporting the survival of different cohorts of births over several time periods. Importantly, we estimated survival for infants born in 2020, which will be useful for counselling parents when a congenital anomaly is diagnosed and for health and social care planning. The gllamm model allows the correlation of survival over time within a study to be modelled whilst allowing for the random effects from different studies. As the included studies used differing birth cohorts with their effect on survival that increased over time, we felt that it would be inappropriate to present I^2^ heterogeneity results that is a standard measure of variation between studies, usually clinical trials. We also did not test for publication bias, as survival studies profoundly differ by their nature from clinical trials where publication bias can be expected due to a higher likelihood of publication of positive results, which is not the case for survival studies. Moreover, as a number of register-based studies included in the meta-analysis estimated survival of many different anomaly groups and types, publication bias for a specific anomaly is unlikely. Owing to the lack of data in terms of the small number of studies, formal tests for publication bias lack power, and funnel plots were not informative. The paucity of data limits the predictive capabilities of the models, as shown by the wide confidence intervals on some estimates. A further limitation is the assumption that improvements in survival in the past will continue to be maintained in the future. This is a particular issue with Down syndrome children with CHD. There have been recent dramatic improvements in their survival, but such improvements are unlikely to continue, and it is likely that their survival will always be slightly lower than that of children with Down syndrome without CHD. Yet the two models predicted very similar survival for such children born in 2020.

Meta-analysis was not possible for all studies included in this systematic review, as either there was an insufficient number of studies reporting survival for the same anomaly subtype or the studies did not report 95% CI or the number of cases. Moreover, not all studies included in the meta-analysis of some structural anomalies (e.g., spina bifida, CDH) were consistent in their exclusion of non-isolated anomalies, which may have accounted for the variability in the survival estimates. All but one of the included studies were conducted in high-income countries, which limited generalisability of the results to low-income countries. Lack of relevant studies from 66 papers identified from our Medline search not restricted to English language, most of which were from Europe, suggests that population-based studies with long-term follow-up of children with congenital anomalies or linkage studies to identify deaths beyond infancy are rare in low-income countries.

The papers analysing survival predictors were not systematically searched for; only studies eligible for this review that also explored predictors were included. We acknowledge that summarised data on survival predictors reported in the reviewed studies are supplementary and enrich the interpretation of the results but are not a comprehensive review of predictors of congenital anomaly–related survival. Therefore, the association of survival with some important risk factors such as maternal deprivation, shown to be linked to lower infant and child survival [89,93], including children born with congenital anomalies [94], could have been underinvestigated in this review.

This systematic review and meta-analysis summarised the existing international evidence from population-based studies to provide information on long-term survival of children with selected congenital anomalies and temporal changes in survival. Our findings reveal a wide variation in survival by congenital anomaly subtype and suggest reduced survival associated with many anomaly subtypes compared with the reference population. The meta-analysis has demonstrated that survival has significantly improved over time for a number of specific congenital anomalies. We have also provided predicted survival estimates for children born in 2020. This information has important implications for the planning and delivery of public health services, specialised medical care, and educational services and is valuable for clinicians, public health professionals, healthcare providers, and parents. We identified a lack of good-quality, reasonably sized studies for many congenital anomaly subtypes that prevented estimation of their pooled survival and analysis of trends over time. Future survival studies should endeavour to use multicentre case data from different parts of the world linked to reliable mortality data with follow-up from birth to avoid selection bias and underascertainment of deaths.

## Supporting information

S1 Fig Survival estimates (with 95% CI) of children with encephalocele at 1 year of age over time (4 studies). The numbers at survival points indicate the included study: 1—Siffel, 2003, Atlanta, USA; 2—Tennant, 2010, Northern England; 3—Wang, 2011, USA; 4—Wang, 2015, USA. Survival at 5 years was not plotted, as survival data were available for three studies only. 95% CI, 95% confidence interval.
(TIF)

S2 Fig Survival with native liver (with 95% CI) of children with biliary atresia at 5 (a) and 10 (b) years of age over time (11 birth cohorts from 9 studies). The numbers at survival points indicate the included study, which may appear more than once if survival was reported for more than one birth cohort: 1—McKiernan, 2000, UK and Ireland; 3—Nio, 2003, Japan; 7—Schreiber, 2007, Canada; 8—Wildhaber, 2008, Switzerland; 9—Davenport, 2011, England and Wales, 10—Chardot, 2013, France; 11—Pakarinen, 2018, Nordic countries; Brazil; 13—Grizelj, 2010, Croatia; 15—Tu, 2015, South Australia. 95% CI, 95% confidence interval.
(TIF)

S3 Fig Survival estimates (with 95% CI) of children with trisomy 18 (4 studies). The numbers at survival points indicate the included study: 1—Meyer, 2016, USA; 2—Nelson, 2016, Ontario, Canada; 3—Schneuer, 2019, New South Wales, Australia; 4—Wang, 2011, USA. 95% CI, 95% confidence interval.
(TIF)

S1 Table Search terms and search results in electronic databases Medline, Embase, and PsycInfo. (DOCX)

S2 Table Data extraction form and Newcastle-Ottawa Quality Assessment Scale for cohort studies. (DOCX)

S3 Table Quality-assessment scores of the included studies using the Newcastle-Ottawa Quality Assessment Scale for cohort studies. (DOCX)

S4 Table Details of sources of case ascertainment and death identification of included studies and description of a comparison group. (DOCX)

S5 Table Survival estimates by congenital anomaly type at age 1–25 years, overall and by risk factor. (DOCX)

S6 Table Predictors of survival/mortality in the included studies that explored risk factors associated with survival at different age points, including infancy (*n* = 35), by congenital anomaly group/subtype. (DOCX)

S1 PRISMA Checklist PRISMA, Preferred Reporting Items for Systematic Reviews and Meta-Analyses. (DOCX)

S1 Text Protocol for PROSPERO registration. PROSPERO, International Prospective Register of Systematic Reviews.
(DOCX)

## Abbreviations

95% CI: 95% confidence interval
AGA: appropriate for gestational age
aRR: adjusted RR
BASM: biliary atresia splenic malformation syndrome
BPA: British Paediatric Association
CAVD: complete atrio-ventricular defect
CDH: congenital diaphragmatic hernia
CHD: congenital heart defect
ECMO: extracorporeal membrane oxygenation
EUROCAT: European Surveillance of Congenital Anomalies
HR: hazard ratio
ICD: International Classification of Disease
IMD: Index of Multiple Deprivation
KP: Kasai hepatoportoenterostomy
LBW: low birth weight
LGA: large for gestational age
MBR: Medical Birth Registry
NH: Non-Hispanic
NHB: non-Hispanic Black
NHW: non-Hispanic White
NLS: native liver survival
NOS: Newcastle-Ottawa Quality Assessment Scale
OR: odds ratio
PICOS: Population/Patient, Intervention/Exposure, Comparator group, Outcome, Study design
PRISMA: Preferred Reporting Items for Systematic Reviews and Meta-Analyses
PROSPERO: International Prospective Register of Systematic Reviews
PWS: Prader-Willi syndrome
RR: relative risk
SGA: small for gestational age
SMR: standardised mortality ratio
TOPFA: termination of pregnancy for fetal anomaly
UN: United Nations
