# Supplementary figures and images for: Long-term survival of children born with congenital anomalies: A systematic review and meta-analysis of population-based studies
Source: PLoS Med. 2020 Sep 28;17(9):e1003356. doi: 10.1371/journal.pmed.1003356 (PMC7521740; doi:10.1371/journal.pmed.1003356)

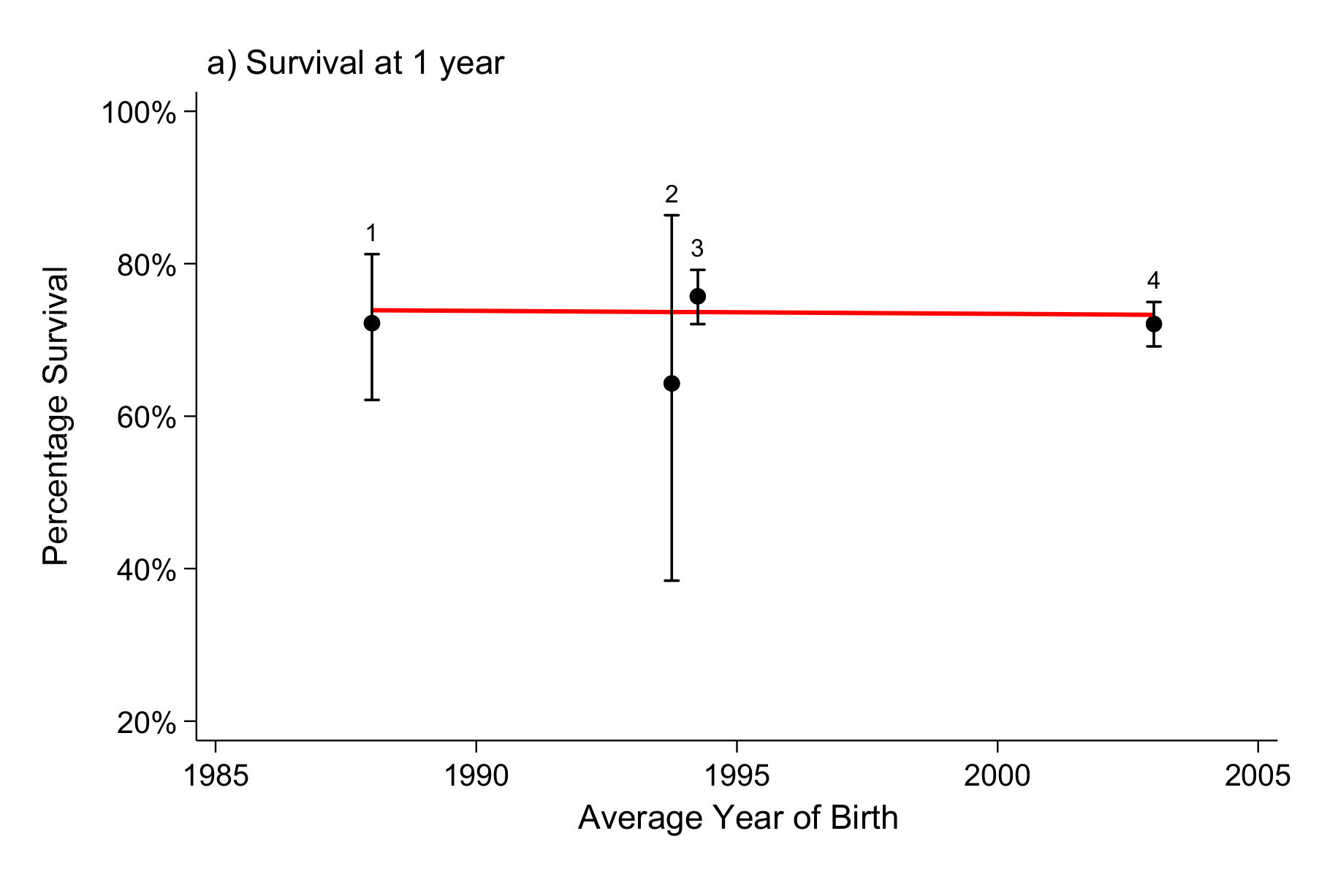

Supplement: S1 Fig — The numbers at survival points indicate the included study: 1—Siffel, 2003, Atlanta, USA; 2—Tennant, 2010, Northern England; 3—Wang, 2011, USA; 4—Wang, 2015, USA. Survival at 5 years was not plotted, as survival data were available for three studies only. 95% CI, 95% confidence interval. (TIF) [file pmed.1003356.s001.tif]

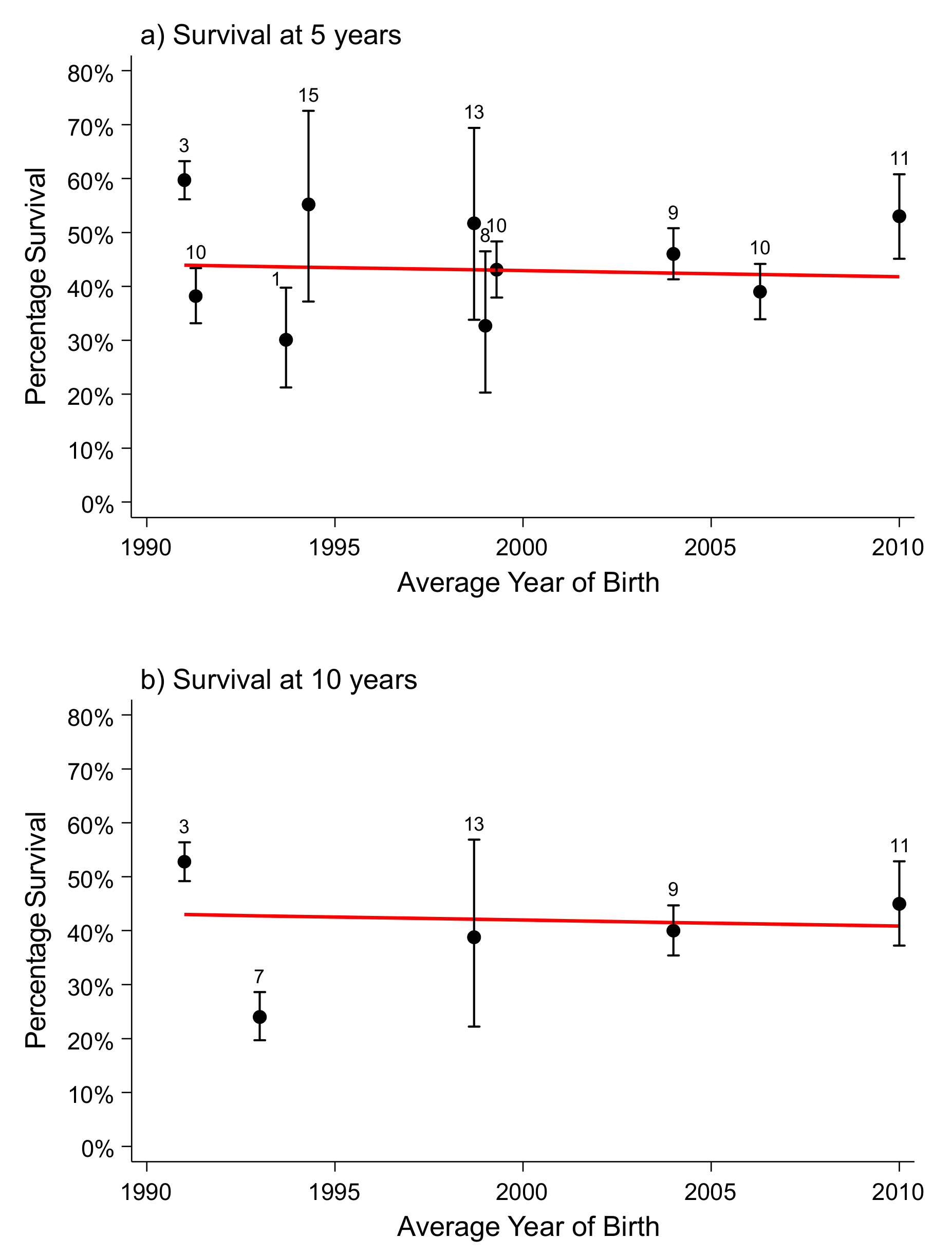

Supplement: S2 Fig — The numbers at survival points indicate the included study, which may appear more than once if survival was reported for more than one birth cohort: 1—McKiernan, 2000, UK and Ireland; 3—Nio, 2003, Japan; 7—Schreiber, 2007, Canada; 8—Wildhaber, 2008, Switzerland; 9—Davenport, 2011, England and Wales, 10—Chardot, 2013, France; 11—Pakarinen, 2018, Nordic countries; Brazil; 13—Grizelj, 2010, Croatia; 15—Tu, 2015, South Australia. 95% CI, 95% confidence interval. (TIF) [file pmed.1003356.s002.tif]

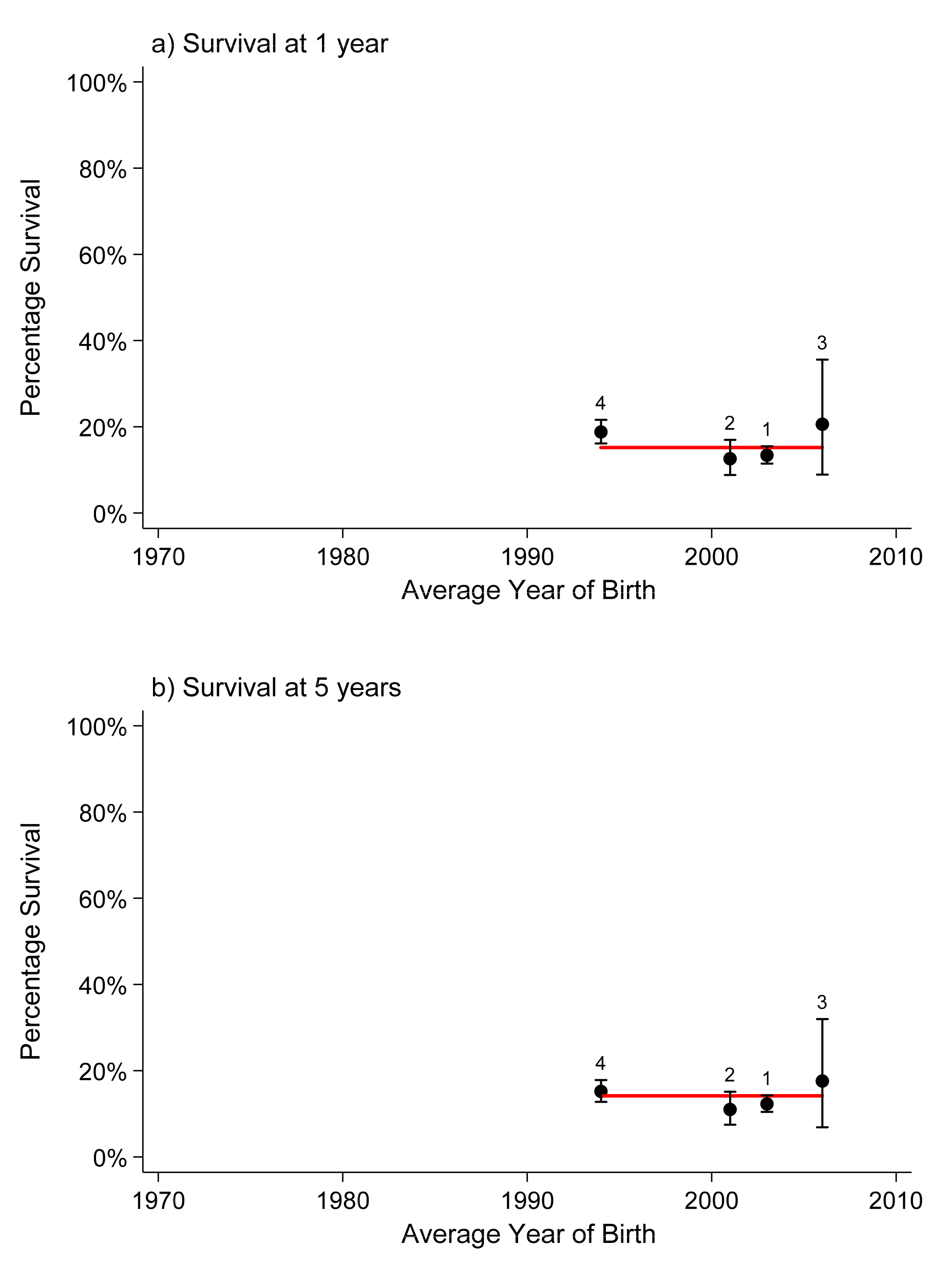

Supplement: S3 Fig — The numbers at survival points indicate the included study: 1—Meyer, 2016, USA; 2—Nelson, 2016, Ontario, Canada; 3—Schneuer, 2019, New South Wales, Australia; 4—Wang, 2011, USA. 95% CI, 95% confidence interval. (TIF) [file pmed.1003356.s003.tif]
