# Supplementary material for: Long-term survival of children born with congenital anomalies: A systematic review and meta-analysis of population-based studies
Source: PLoS Med. 2020 Sep 28;17(9):e1003356. doi: 10.1371/journal.pmed.1003356 (PMC7521740; doi:10.1371/journal.pmed.1003356)

# S1 Table. Search terms and search results in electronic databases Medline, Embase and PsycInfo.

Medline (search on 02.05.18)


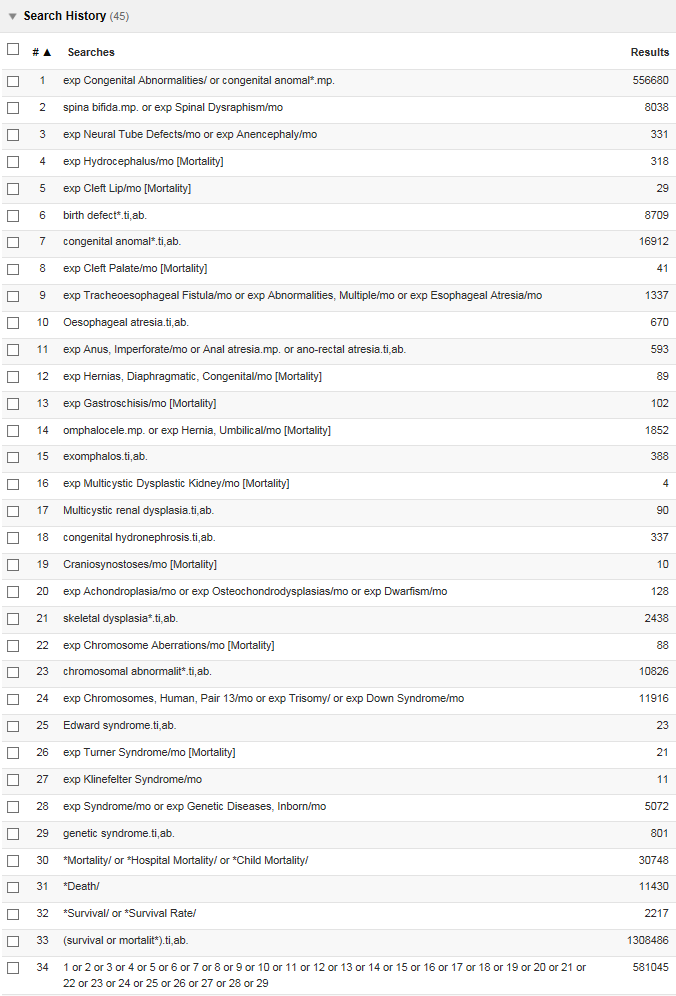


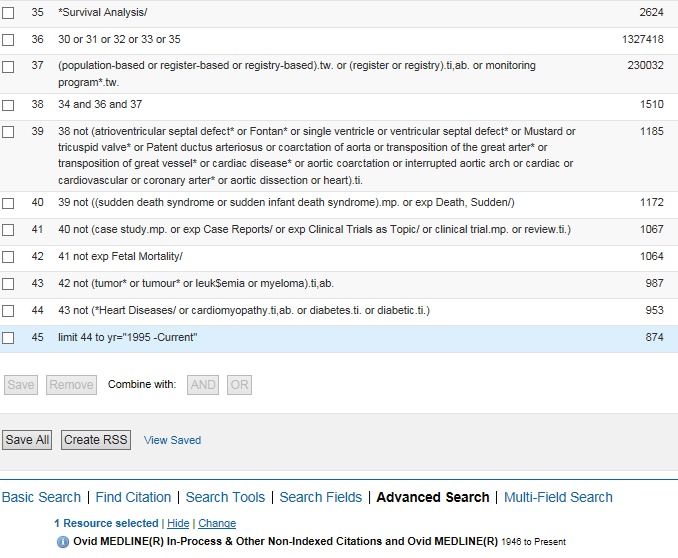


Embase (search on 02.05.18)


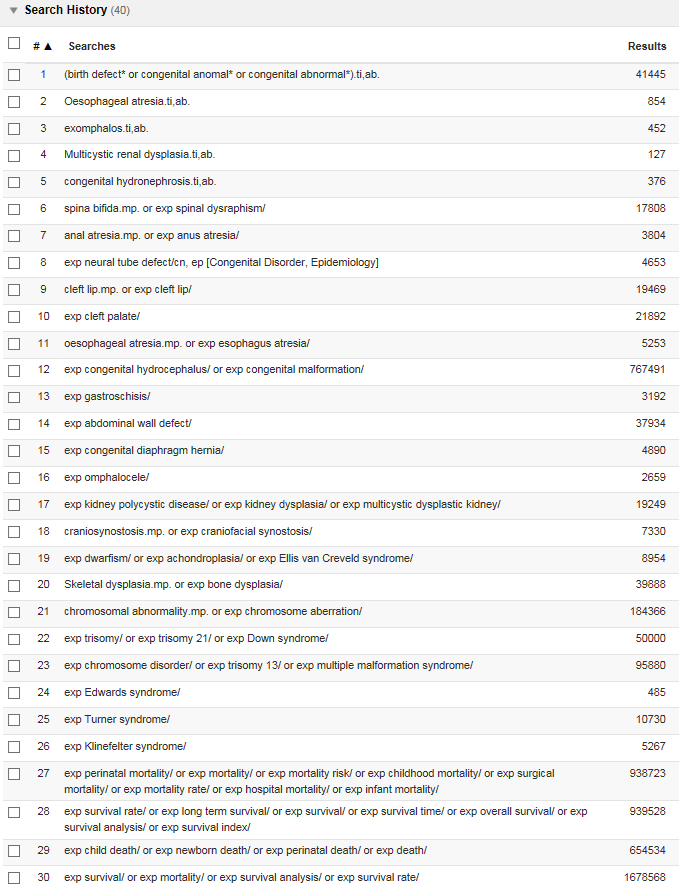


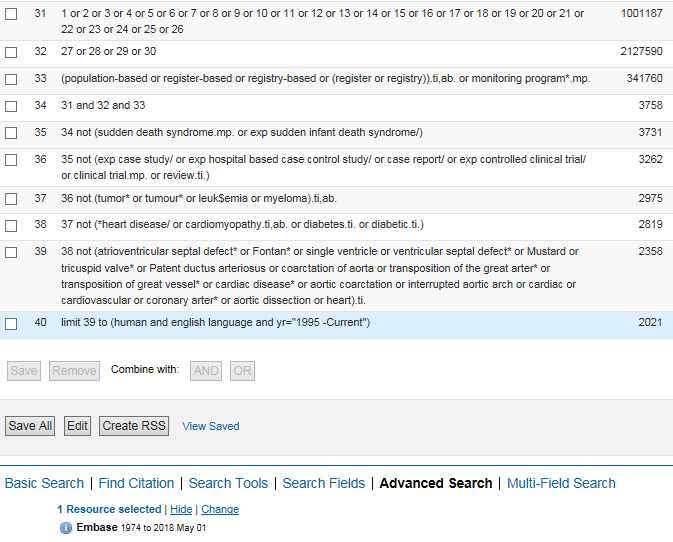


PsycInfo - Search on 02/05/18

-
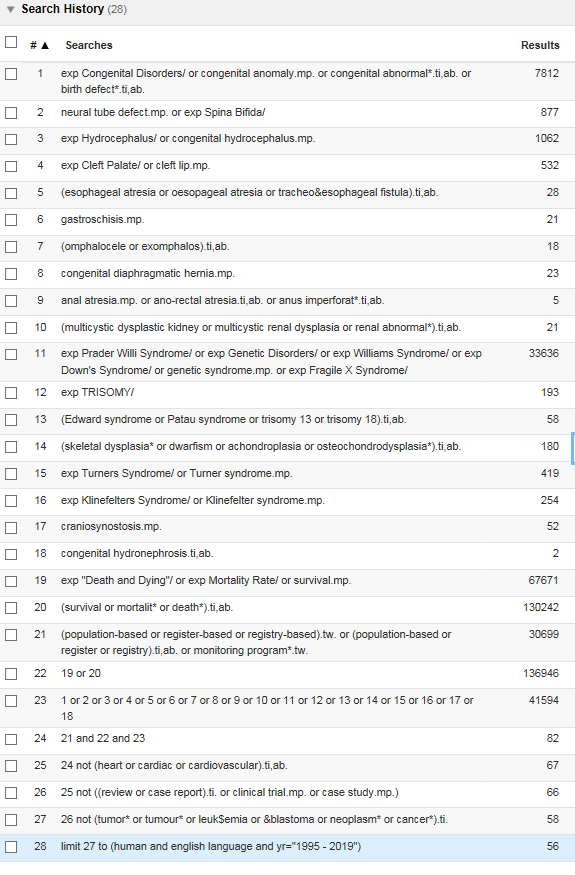


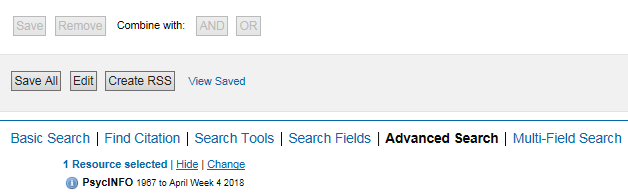


Medline (Search update on 26.03.2019)


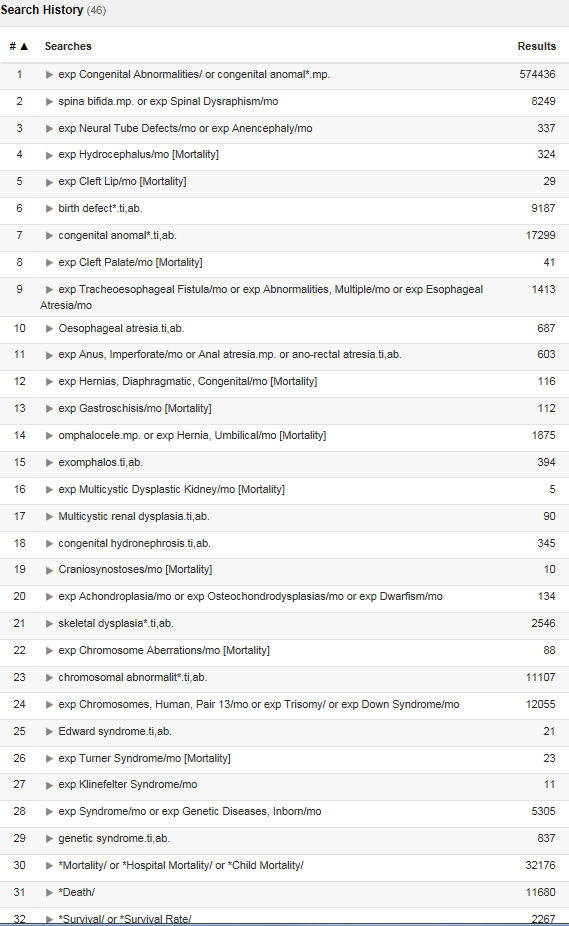


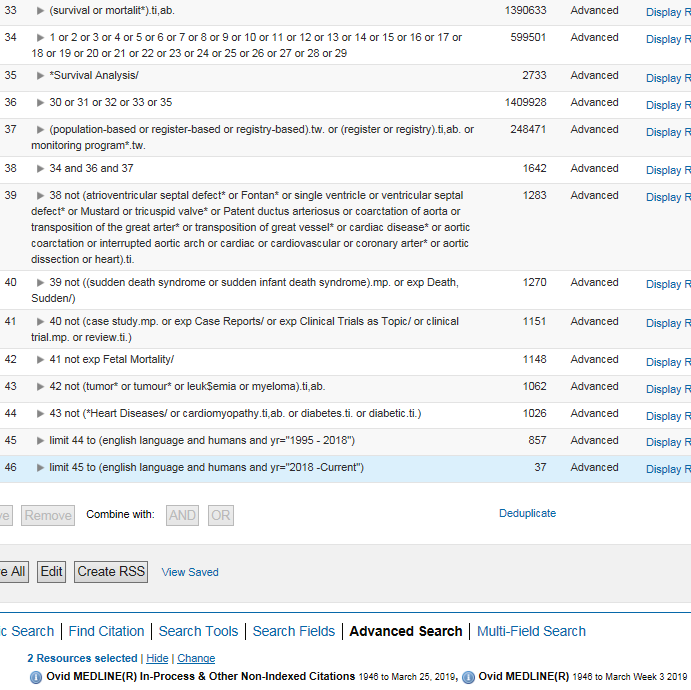


Embase (search on 26.03.19)


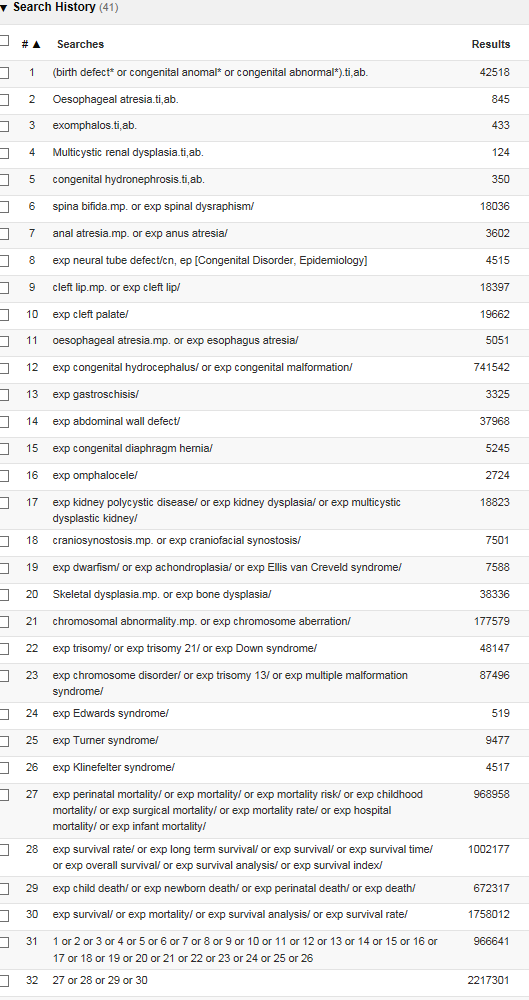


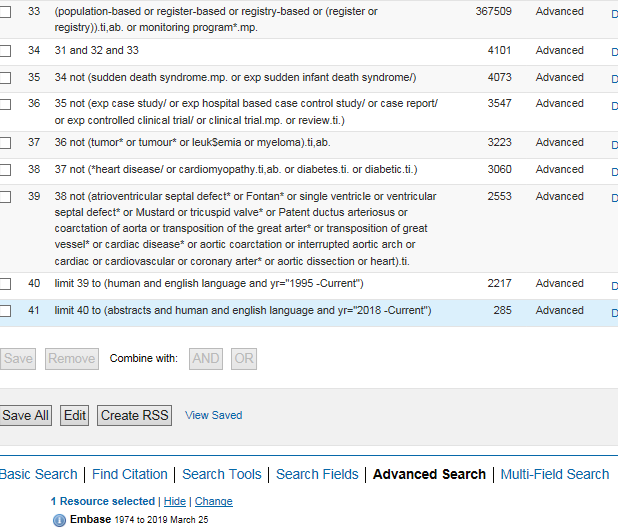


PsycInfo - Search on 26.03.19


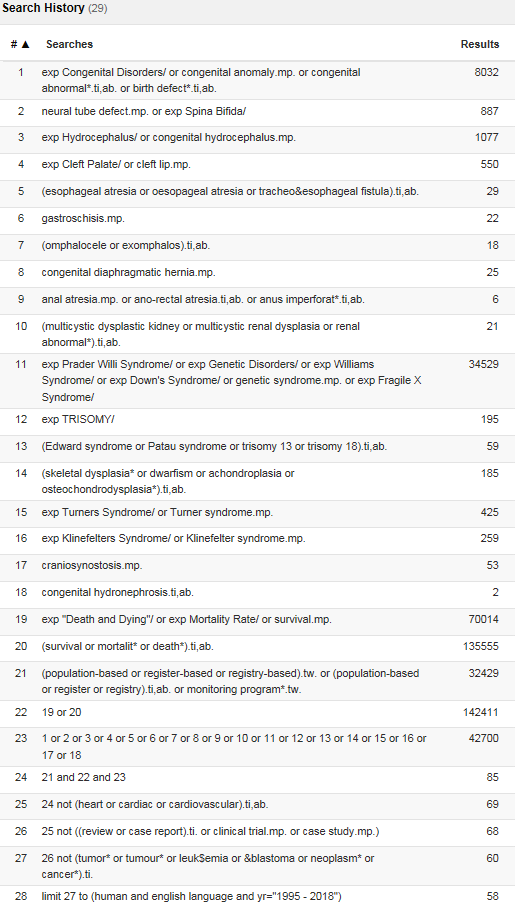


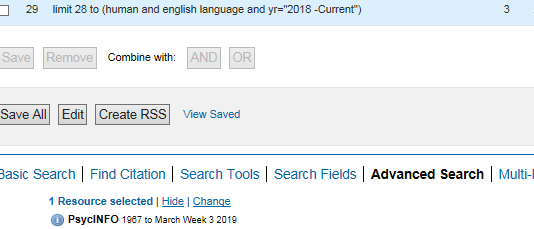

Supplement: S1 Table — (DOCX) [file pmed.1003356.s004.docx]
