# Supplementary material for: Long-term survival of children born with congenital anomalies: A systematic review and meta-analysis of population-based studies
Source: PLoS Med. 2020 Sep 28;17(9):e1003356. doi: 10.1371/journal.pmed.1003356 (PMC7521740; doi:10.1371/journal.pmed.1003356)
Supplement: S2 Table — (DOCX) [file pmed.1003356.s005.docx]

**S2 Table. Data extraction form and Newcastle-Ottawa Quality Assessment Scale for cohort studies.**

| Reviewer name |  | | | | |
| --- | --- | --- | --- | --- | --- |
| Manuscript title |  | | | | |
| First Author |  | | | | |
| Publication year |  | | | | |
| Study location |  | | | | |
| Study period | Cases born  Between……………………………..and …………………………….  Specify years of birth for different survival estimates (e.g. for 1-year survival, for 5-yr survival etc)  Clinical procedures  Other, please state: ………………………………………….  Deaths included between………………………….and………………………………… | | | | |
| Number of cases with a specific anomaly (specify group/subtype: | Complete here if the article is focused on a specific CA(s) – complete Table 1 below (or attach a copy of the relevant table in the paper) if a number of CA groups/subtypes is reported | | | | |
| Number of deaths (specify group/subtype: | Complete here if the article is focused on a specific CA(s) – complete Table 1 below (or attach a copy of the relevant table in the paper) if all/many CA groups/subtypes are reported | | | | |
| Type of survival/ mortality *(tick all that are reported in the manuscript)* | Survival/Mortality by age :  1 y,  2 y,  5 y,  10 y,  15 y,  20 y  Other age: Click here to enter text.  Mortality before date Click here to enter text. (e.g before Jan 2010)  Other (e.g. post-operative mortality), please state: Click here to enter text. | | | | |
| Included cases | All cases  Other, please state: Click here to enter text. | | | | |
| Type of congenital anomaly included *(tick all that apply) –* most studies analyse survival in a specific CA group/subtype – specify if CA reported is isolated or with associated anomalies | All CAs (regardless of separation by group or subtype)  Anomalies of the nervous system  Neural tube defects  Anencephaly  Encephalocele  Spina bifida  Hydrocephalus  Other anomalies of the nervous system (microcephaly, holoprosencephaly etc)  Eye anomalies  Ear, face and neck anomalies  Respiratory system  Oral clefts  Cleft lip  Cleft lip and palate  Cleft palate  Cleft lip with or without cleft palate  Digestive system  Oesophageal atresia  Duodenal atresia or stenosis  Diaphragmatic hernia  Other anomalies of the digestive system (e.g. anorectal atresia, biliary atresia – specify Click here to enter text.)  Abdominal wall defects  Omphalocele  Gastroschisis  Congenital malformations of genital organs  Urinary system  Musculoskeletal  Limb reduction  Upper limb  Lower limb  Chromosomal  Down syndrome(trisomy 21)  Patau syndrome (trisomy 13)  Edwards syndrome (trisomy 18)  Turner syndrome  Other chromosomal  Syndrome (monogenic or with unknown cause)  Other, please state Click here to enter text. | | | | |
| Specify how cases with associated or multiple anomalies were coded |  | | | | |
| Data sources | population-based study -  Yes  No  For cases:  For deaths: | | | | |
| **Study results** | Complete the rows below if the number of analysed anomalies is limited to one or two groups or subtypes | | | | |
| Survival rate/estimate/probability by CA group/subtype (with 95% CI if provided) | Congenital anomaly subtype:  By 1 month (28 days):  By 1 year:  By 2 years:  By 5 years:  By 10 years:  By 15 years:  By 20 years:  By 25 years:  More advanced age: | | | | |
| Are any other measurements of mortality risk used? | No  Hazard ratios (HR)compared to the background population or controls  Standardized mortality ratio (SMR)  Mortality rate (e.g. CA-specific under-5-years child mortality  Relative risk (RR)  other (specify) | | | | |
| Were any risk factors/predictors of survival analysed? | Yes  No  If Yes, please complete the rows below: | | | | |
| *Add as many rows as necessary for the sections below* |  | | | | |
| Unadjusted Results  *(if applicable)*  *Specify if this analysis was done for infant survival/mortality only or for the overall survival* | **Factors. analysed**  ***(select one)***  Infant sex  Birthweight (BW) group  Gestational age (GA) group  BW and GA category  Plurality (single vs multiple)  Age at diagnosis  Birth year/ period  Surgery/procedure (e.g. operative cerebrospinal fluid (CSF) drainage  Maternal age  Parity  Maternal race/ethnicity  Maternal education  Maternal socioeconomic status (SES) measured by other measures (e.g. area-based index of deprivation) specify  Number of birth defects (isolated vs non-isolated)  Other, please state: | | | **Type of result:**  ***(select one)***  Hazard ratios  Odds ratio  Relative risk  Survival rate  Mortality rate  Other, please state: | |
|  | Category 1: | | | Result 1 (including 95% CIs & p-values where appropriate): | |
|  | Category 2: | | | Result 2: | |
|  | Category 3 | | | Result 3: | |
|  | Category 4 | | | Result 4: | |
|  | Category 5 | | | Result 5: | |
|  | Overall P-value: | | | | |
| Unadjusted Results  *(if applicable)* | **Factors. analysed**  ***(select one)***  Infant sex  Birthweight (BW) group  Gestational age (GA) group  BW and GA category  Plurality (single vs multiple)  Age at diagnosis  Birth year/ period  Surgery/procedure (e.g. operative cerebrospinal fluid (CSF) drainage  Maternal age  Parity  Maternal race/ethnicity  Maternal education  Maternal socioeconomic status (SES) measured by other measures (e.g. area-based index of deprivation) specify  Number of CAs (isolated vs non-isolated)  Other, please state: | | **Type of result:**  ***(select one)***  Hazard ratios  Odds ratio  Relative risk  Survival rate  Mortality rate  Other, please state: | | |
|  | Category 1: | | Result 1 (including 95% CIs & p-values where appropriate): | | |
|  | Category 2: | | Result 2: | | |
|  | Category 3 | | Result 3: | | |
|  | Category 4 | | Result 4: | | |
|  | Category 5 | | Result 5: | | |
|  | Overall P-value: | | | | |
| Adjusted Results  *(if applicable)* | | **Factors. analysed**  ***(select one)***  Infant sex  Birthweight (BW) group  Gestational age (GA) group  BW and GA category  Plurality (single vs multiple)  Age at diagnosis  Birth year/ period  Surgery/procedure (e.g. operative cerebrospinal fluid (CSF) drainage  Maternal age  Parity  Maternal race/ethnicity  Maternal education  Maternal socioeconomic status (SES) measured by other measures (e.g. area-based index of deprivation) specify  Number of CAs (isolated vs non-isolated)  Other, please state: | | | **Type of result:**  ***(select one)***  Hazard ratios  Odds ratio  Relative risk  Survival rate  Mortality rate  Other, please state  **Adjusted for:** |
|  |  | Category 1: | | | Result 1 (including 95% CIs & p-values where appropriate): |
|  |  | Category 2: | | | Result 2: |
|  |  | Category 3 | | | Result 3: |
|  |  | Category 4 | | | Result 4: |
|  |  | Category 5 | | | Result 5: |
|  |  | Overall P-value: | | | Result 5: |
|  |  | Overall P-value: | | | |
| Other Results 1  *(if applicable)* | | **Factors. analysed**  ***(select one)***  Infant sex  Birthweight (BW) group  Gestational age (GA) group  BW and GA category  Plurality (single vs multiple)  Age at diagnosis  Year/year period of birth  Surgery/procedure (e.g. operative cerebrospinal fluid (CSF) drainage  Maternal age  Parity  Maternal race/ethnicity  Maternal education  Maternal socioeconomic status (SES) measured by other measures (e.g. area-based index of deprivation) specify  Number CAs (isolated vs non-isolated)  Other, please state: | | | **Type of result:**  ***(select one)***  Hazard ratios  Odds ratio  Relative risk  Survival rate  Mortality rate  Other, please state:  **Adjusted?**  Adjusted  Unadjusted  **Adjusted for:** |
|  |  | Category 1: | | | Result 1: |
|  |  | Category 2: | | | Result 2: |
|  |  | Category 3 | | | Result 3: |
|  |  | Category 4 | | | Result 4: |
|  |  | Category 5 | | | Result 5: |
|  |  | Overall P-value: | | | |
| Do we need to contact the authors? | | Yes  No  If Yes please provide more info: | | | |
| Do you feel after the detailed reviewing the full paper that it should be included for the analysis? | | Yes  No  If No, please explain why it should be excluded: | | | |
| Did you check the reference list for relevant refs, i.e. those that may be included? | | Yes  No  If Yes, please highlight the relevant references in the paper copy and attach to your review or copy across to add at the end of this data extraction form. | | | |

***NEWCASTLE - OTTAWA QUALITY ASSESSMENT SCALE***

***COHORT STUDIES***

Note: A study can be awarded a maximum of one star for each numbered item within the **Selection** and **Outcome** categories. A maximum of two stars can be given for **Comparability**

*Before completing the form, please read the Coding Manual below which we have adopted for our review.*

1) **Selection** (*max of 4 stars can be given*)

Representativeness of the exposed cohort

a) truly representative of the average _______________ (describe) in the community ****

b) somewhat representative of the average ______________ in the community ****

c) selected group of users eg nurses, volunteers

d) no description of the derivation of the cohort

2) Selection of the non exposed cohort

a) drawn from the same community as the exposed cohort ****

b) drawn from a different source

c) no description of the derivation of the non exposed cohort

3) Ascertainment of exposure

a) secure record (eg surgical records) ****

b) structured interview ****

c) written self report

d) no description

4) Demonstration that outcome of interest was not present at start of study

a) yes ****

b) no

**Comparability** (*max of 2 stars can be given*)

1) Comparability of cohorts on the basis of the design or analysis

a) study controls for _____________ (select the most important factor) ****

b) study controls for any additional factor**** (This criteria could be modified to indicate specific control for a second important factor.)

**Outcome** (*max of 3 stars can be given*)

1) Assessment of outcome

a) independent blind assessment ****

b) record linkage ****

c) self report

d) no description

2) Was follow-up long enough for outcomes to occur

a) yes (select an adequate follow up period for outcome of interest) ****

b) no

3) Adequacy of follow up of cohorts

a) complete follow up - all subjects accounted for ****

b) subjects lost to follow up unlikely to introduce bias - small number lost - > *95*% (select an adequate %) follow up, or description provided of those lost) ****

c) follow up rate <_*95*% (select an adequate %) and no description of those lost

d) no statement

**CODING MANUAL FOR COHORT STUDIES (***text in italic blue has been added to adopt the manual to our review)*

# SELECTION

1. **Representativeness of the Exposed Cohort -**(*i.e in our review, the cohort with congenital anomalies or a specific anomaly*)

Item is assessing the representativeness of exposed individuals in the community, not the representativeness of the sample of women from some general population. For example, subjects derived from groups likely to contain middle class, better educated, health oriented women are likely to be representative of postmenopausal estrogen users while they are not representative of all women (e.g. members of a health maintenance organisation (HMO) will be a representative sample of estrogen users. While the HMO may have an under-representation of ethnic groups, the poor, and poorly educated, these excluded groups are not the predominant users of estrogen).

Allocation of stars as per rating sheet

1. truly representative of the average _______________ (describe) in the community **** - W*e expect the study cohort to include all live births with a congenital anomaly of interest that are followed up from birth to a specified age or year, one of our inclusion criteria (otherwise, we will not obtain the true survival rates) so we expect a star* **** *to be given for this point.*
2. **Selection of the Non-Exposed Cohort**

Allocation of stars as per rating sheet

*If there is a comparison group (controls or the reference population) it should be drawn from the same population.*

1. **Ascertainment of Exposure**

Allocation of stars as per rating sheet

*We expect our population-based cases to be either register-based or based on the hospital (tertiary centre) records using robust inclusion and diagnostic criteria and deaths should be identified from the death registration sources. So we expect* ** **for this point as well.

1. **Demonstration That Outcome of Interest Was Not Present at Start of Study**

In the case of mortality studies, outcome of interest is still the presence of a disease/ incident, rather than death. That is to say that a statement of no history of disease or incident earns a star.

*We include studies that followed up live born children with a congenital anomaly from birth (prenatally diagnosed in many cases) so the outcome of interest (death) was not present at the start of a study even if death occurred within the first 24 hrs. We excluded studies that used death certificates as a primary source of the diagnosis of a congenital anomaly (underlying cause) where the number of children born with a specific anomaly is unknown. So we expect the answer ‘Yes’ here.*

***COMPARABILITY***

1. **Comparability of Cohorts on the Basis of the Design or Analysis**

A maximum of 2 stars can be allotted in this category

Either exposed and non-exposed individuals must be matched in the design and/or confounders must be adjusted for in the analysis. Statements of no differences between groups or that differences were not statistically significant are not sufficient for establishing comparability. Note: If the relative risk for the exposure of interest is adjusted for the confounders listed, then the groups will be considered to be comparable on each variable used in the adjustment.

There may be multiple ratings for this item for different categories of exposure (e.g. ever vs. never, current vs. previous or never)

Age = , Other controlled factors =

*This is irrelevant for studies that have just calculated survival in children with a specific congenital anomaly and did not compare survival/mortality rates between children with a congenital anomaly and those without. For those that report mortality risks, the cohort with a congenital anomaly and the comparison group must be either matched by year and place of birth and/or the analysis has to be adjusted for confounders.*

***OUTCOME***

1. **Assessment of Outcome**

For some outcomes (e.g. fractured hip), reference to the medical record is sufficient to satisfy the requirement for confirmation of the fracture. This would not be adequate for vertebral fracture outcomes where reference to x-rays would be required.

1. Independent or blind assessment stated in the paper, or confirmation of the outcome by reference to secure records (x-rays, medical records, etc.)
2. Record linkage (e.g. identified through ICD codes on database records)
3. Self-report (i.e. no reference to original medical records or x-rays to confirm the outcome)
4. No description.

*We expect our outcome of interest (death) to be identified from the population-based death registration sources and linked to congenital anomaly cases through the record linkage. So we expect a star* **** *for the included studies based on item b.*

1. **Was Follow-Up Long Enough for Outcomes to Occur**

An acceptable length of time should be decided before quality assessment begins (e.g. 5 yrs. for exposure to breast implants)

*We expect a positive (‘yes’) answer here as we will only report the age of survival for cases with a complete follow up. (for example, we will not report 10-year survival for cases that were born 6 years before the end of follow up).*

1. **Adequacy of Follow Up of Cohorts**

This item assesses the follow-up of the exposed and non-exposed cohorts to ensure that losses are not related to either the exposure or the outcome.

Allocation of stars as per rating sheet

*See the explanation for item 2.*
