# Supplementary material for: Long-term survival of children born with congenital anomalies: A systematic review and meta-analysis of population-based studies
Source: PLoS Med. 2020 Sep 28;17(9):e1003356. doi: 10.1371/journal.pmed.1003356 (PMC7521740; doi:10.1371/journal.pmed.1003356)
Supplement: S3 Table — (DOCX) [file pmed.1003356.s006.docx]

**S3 Table.** **Quality assessment scores of the included studies using the Newcastle-Ottawa scale for cohort studies.**

| **Author, publication year, reference** | **Selection** | **Comparability** | **Outcome** | **Category** |
| --- | --- | --- | --- | --- |
| Agha, 2006 [1] | **** | ** | *** | High |
| Bakker, 2019 [2] | *** |  | *** | High |
| Bell, 2016 [3] | **** | * | *** | High |
| Berger, 2003 [4] | **** | * | *** | High |
| Borgstedt-Bakke, 2017 [5] | **** |  | *** | High |
| Brodwall, 2018 [6] | **** | * | *** | High |
| Burgos, 2017 [7] | *** |  | *** | High |
| Cassina, 2016 [8], | *** |  | *** | High |
| Cassina, 2019 [9] | *** |  | ** | Medium |
| Chardot, 2013 [10] | *** |  | *** | High |
| Chua, 2020 [11] | *** |  | *** | High |
| Dastgiri, 2003 [12] | *** |  | *** | High |
| Davenport, 2011 [13] | *** |  | *** | High |
| De Carvalho, 2010 [14] | *** |  | ** | Medium |
| De Vries, 2011 [15] | *** |  | *** | High |
| Eide, 2006 [16] | **** | * | *** | High |
| Folkestad, 2016 [17] | **** | ** | *** | High |
| Frid, 1999 [18] | *** |  | *** | High |
| Garne, 2002 [19] | *** |  | *** | High |
| Glasson, 2016 [20] | **** |  | *** | High |
| Grizelj, 2010 [21] | *** |  | *** | High |
| Gudbjartsson, 2008 [22] | *** |  | *** | High |
| Halliday, 2009 [23] | *** |  | *** | High |
| Hayes, 1997 [24] | *** |  | *** | High |
| Hinton, 2017 [25] | *** |  | *** | High |
| Jaillard, 2003 [26] | *** |  | *** | High |
| Kucik, 2013 [27] | *** |  | *** | High |
| Lampela, 2012 [28] | *** |  | *** | High |
| Leonard, 2000 [29] | *** |  | *** | High |
| Leonhardt, 2011 [30] | *** |  | ** | Medium |
| Lionti, 2012 [31] | *** |  | ** | Medium |
| Löf Granström, 2017 [32] | **** | * | *** | High |
| McKiernan, 2000 [33] | *** |  | *** | High |
| McKiernan, 2009 [34] | *** |  | *** | High |
| Meyer, 2016 [35] | *** |  | *** | High |
| Nelson, 2016 [36] | *** |  | *** | High |
| Nembhard, 2010 [37] | *** |  | *** | High |
| Nio, 2003 [38] | *** |  | *** | High |
| Oddsberg, 2012 [39] | **** | ** | *** | High |
| Pakarinen, 2018 [40] | *** |  | *** | High |
| Rankin, 2012 [41] | *** |  | *** | High |
| Rasmussen, 2006 [42] | *** |  | *** | High |
| Risby, 2017 [43] | *** |  | *** | High |
| Schneuer, 2019 [44] | *** |  | *** | High |
| Schreiber, 2007 [45] | *** |  | *** | High |
| Shin, 2012 [46] | *** |  | *** | High |
| Siffel, 2003 [47] | *** |  | *** | High |
| Simmons, 2014 [48] | *** |  | *** | High |
| Sutton, 2008 [49] | *** |  | *** | High |
| Tennant, 2010 [50] | *** |  | *** | High |
| Tu, 2015 [51] | *** |  | *** | High |
| Wang, 2011 [52] | *** |  | ** | Medium |
| Wang, 2015 [53] | *** |  | ** | Medium |
| Wildhaber, 2008 [54] | *** |  | *** | High |
| Wong, 2001 [55] | *** |  | *** | High |

**Note:**

A study can be awarded a maximum of one star for each numbered item within the Selection (maximum 4 stars) and Outcome (maximum 3 stars) categories. A maximum of two stars can be given for Comparability. In total, maximum 9 scores can be given, however, maximum 6 stars only for survival studies with no comparison group. Quality categories were allocated as: Low = 0-2 stars, Medium = 3-5 stars, High = 6-9 stars.

**References**

1. Agha MM, Williams JI, Marrett L, To T, Dodds L. Determinants of survival in children with congenital abnormalities: a long-term population-based cohort study. Birth Defects Res A Clin Mol Teratol. 2006;76(1):46-54. PMID: 16397887.

2. Bakker MK, Kancherla V, Canfield MA, Bermejo-Sanchez E, Cragan JD, Dastgiri S, et al. Analysis of Mortality among Neonates and Children with Spina Bifida: An International Registry-Based Study, 2001-2012. Paediatr Perinat Epidemiol. 2019;33(6):436-48. doi: 10.1111/ppe.12589. PMID: 31637749.

3. Bell JC, Nassar N, Bower C, Turner RM, Raynes-Greenow C. Long-term survival for infants born with orofacial clefts in Western Australia. Birth Defects Res A Clin Mol Teratol. 2016;106(3):172-7. doi: <https://dx.doi.org/10.1002/bdra.23473>. PMID: 26663708.

4. Berger KH, Zhu BP, Copeland G. Mortality throughout early childhood for Michigan children born with congenital anomalies, 1992-1998. Birth Defects Res A Clin Mol Teratol. 2003;67(9):656-61. PMID: 14703790.

5. Borgstedt-Bakke JH, Fenger-Gron M, Rasmussen MM. Correlation of mortality with lesion level in patients with myelomeningocele: a population-based study. J Neurosurg Pediatrics. 2017;19(2):227-31. doi: <https://dx.doi.org/10.3171/2016.8.PEDS1654>. PMID: 27911247.

6. Brodwall K, Greve G, Leirgul E, Klungsøyr K, Holmstrøm H, Vollset SE, et al. The five-year survival of children with Down syndrome in Norway 1994–2009 differed by associated congenital heart defects and extracardiac malformations. Acta Paediatr Int J Paediatr. 2018;107(5):845-53. doi: 10.1111/apa.14223.

7. Burgos CM, Frenckner B. Addressing the hidden mortality in CDH: A population-based study. J Pediatr Surg. 2017;52(4):522-5. doi: <https://dx.doi.org/10.1016/j.jpedsurg.2016.09.061>. PMID: 27745705.

8. Cassina M, Ruol M, Pertile R, Midrio P, Piffer S, Vicenzi V, et al. Prevalence, characteristics, and survival of children with esophageal atresia: A 32-year population-based study including 1,417,724 consecutive newborns. Birth Defects Res A Clin Mol Teratol. 2016;106(7):542-8. doi: <https://dx.doi.org/10.1002/bdra.23493>. PMID: 26931365.

9. Cassina M, Fascetti Leon F, Ruol M, Chiarenza SF, Scire G, Midrio P, et al. Prevalence and survival of patients with anorectal malformations: A population-based study. J Pediatr Surg. 2019;54(10):1998-2003. doi: <https://dx.doi.org/10.1016/j.jpedsurg.2019.03.004>. PMID: 30935729.

10. Chardot C, Buet C, Serinet MO, Golmard JL, Lachaux A, Roquelaure B, et al. Improving outcomes of biliary atresia: French national series 1986-2009. J Hepatol. 2013;58(6):1209-17. doi: 10.1016/j.jhep.2013.01.040. PMID: 23402746.

11. Chua GT, Tung KTS, Wong ICK, Lum TYS, Wong WHS, Chow CB, et al. Mortality Among Children with Down syndrome in Hong Kong: A Population-Based Cohort Study from Birth. J Pediatr. 2020;218:138-45. doi: <http://dx.doi.org/10.1016/j.jpeds.2019.11.006>. PMID: 2004554180.

12. Dastgiri S, Gilmour WH, Stone DH. Survival of children born with congenital anomalies. Arch Dis Child. 2003;88(5):391-4. PMID: 12716706.

13. Davenport M, Ong E, Sharif K, Alizai N, McClean P, Hadzic N, et al. Biliary atresia in England and Wales: results of centralization and new benchmark. J Pediatr Surg. 2011;46(9):1689-94. doi: 10.1016/j.jpedsurg.2011.04.013. PMID: 21929975.

14. De Carvalho E, Santos JL, Silveira TR, Kieling CO, Silva LR, Porta G, et al. Biliary atresia: the Brazilian experience. J Pediatr (Rio J). 2010;86(6):473-9. doi: 10.2223/JPED.2054. PMID: 21140036.

15. de Vries W, Homan-Van der Veen J, Hulscher JB, Hoekstra-Weebers JE, Houwen RH, Verkade HJ, et al. Twenty-year transplant-free survival rate among patients with biliary atresia. Clin Gastroenterol Hepatol. 2011;9(12):1086-91. doi: 10.1016/j.cgh.2011.07.024. PMID: 21820397.

16. Eide MG, Skjaerven R, Irgens LM, Bjerkedal T, Oyen N. Associations of birth defects with adult intellectual performance, disability and mortality: population-based cohort study. Pediatr Res. 2006;59(6):848-53. PMID: 16641211.

17. Folkestad L, Hald JD, Canudas-Romo V, Gram J, Hermann AP, Langdahl B, et al. Mortality and Causes of Death in Patients With Osteogenesis Imperfecta: A Register-Based Nationwide Cohort Study. J Bone Miner Res. 2016;31(12):2159-66. doi: <https://dx.doi.org/10.1002/jbmr.2895>. PMID: 27345018.

18. Frid C, Drott P, Lundell B, Rasmussen F, Anneren G. Mortality in Down's syndrome in relation to congenital malformations. J Intellect Disabil Res. 1999;43 ( Pt 3):234-41. PMID: 10392609.

19. Garne E, Rasmussen L, Husby S. Gastrointestinal malformations in Funen county, Denmark - epidemiology, associated malformations, surgery and mortality. Eur J Pediatr Surg. 2002;12(2):101-6. PMID: 12015653.

20. Glasson EJ, Jacques A, Wong K, Bourke J, Leonard H. Improved Survival in Down Syndrome over the Last 60 Years and the Impact of Perinatal Factors in Recent Decades. J Pediatr. 2016;169:214-20.e1. doi: <https://dx.doi.org/10.1016/j.jpeds.2015.10.083>. PMID: 26651430.

21. Grizelj R, Vukovic J, Novak M, Batinica S. Biliary atresia: the Croatian experience 1992-2006. Eur J Pediatr. 2010;169(12):1529-34. doi: 10.1007/s00431-010-1266-8. PMID: 20669030.

22. Gudbjartsson T, Gunnarsdottir A, Topan CZ, Larsson LT, Rosmundsson T, Dagbjartsson A. Congenital diaphragmatic hernia: Improved surgical results should influence abortion decision making. Scand J Surg. 2008;97(1):71-6. PMID: 351449967.

23. Halliday J, Collins V, Riley M, Youssef D, Muggli E. Has prenatal screening influenced the prevalence of comorbidities associated with Down syndrome and subsequent survival rates? Pediatrics. 2009;123(1):256-61. doi: <https://dx.doi.org/10.1542/peds.2007-2840>. PMID: 19117890.

24. Hayes C, Johnson Z, Thornton L, Fogarty J, Lyons R, O'Connor M, et al. Ten-year survival of Down syndrome births. Int J Epidemiol. 1997;26(4):822-9. PMID: 9279615.

25. Hinton CF, Siffel C, Correa A, Shapira SK. Survival Disparities Associated with Congenital Diaphragmatic Hernia. Birth Defects Res A Clin Mol Teratol. 2017;109(11):816-23. doi: 10.1002/bdr2.1015. PMID: 28398654.

26. Jaillard SM, Pierrat V, Dubois A, Truffert P, Lequien P, Wurtz AJ, et al. Outcome at 2 years of infants with congenital diaphragmatic hernia: a population-based study. Ann Thorac Surg. 2003;75(1):250-6. PMID: 12537224.

27. Kucik JE, Shin M, Siffel C, Marengo L, Correa A. Trends in survival among children with down syndrome in 10 regions of the united states. Pediatrics. 2013;131(1):e27-e36. doi: <http://dx.doi.org/10.1542/peds.2012-1616>. PMID: 368184663.

28. Lampela H, Ritvanen A, Kosola S, Koivusalo A, Rintala R, Jalanko H, et al. National centralization of biliary atresia care to an assigned multidisciplinary team provides high-quality outcomes. Scand J Gastroenterol. 2012;47(1):99-107. doi: <https://dx.doi.org/10.3109/00365521.2011.627446>. PMID: 22171974.

29. Leonard S, Bower C, Petterson B, Leonard H. Survival of infants born with Down's syndrome: 1980-96. Paediatr Perinat Epidemiol. 2000;14(2):163-71. PMID: 10791661.

30. Leonhardt J, Kuebler JF, Leute PJ, Turowski C, Becker T, Pfister ED, et al. Biliary atresia: lessons learned from the voluntary German registry. Eur J Pediatr Surg. 2011;21(2):82-7. doi: <https://dx.doi.org/10.1055/s-0030-1268476>. PMID: 21157692.

31. Lionti T, Reid SM, Rowell MM. Prader-Willi syndrome in Victoria: mortality and causes of death. J Paediatr Child Health. 2012;48(6):506-11. doi: <https://dx.doi.org/10.1111/j.1440-1754.2011.02225.x>. PMID: 22697408.

32. Löf Granström A, Wester T. Mortality in Swedish patients with Hirschsprung disease. Pediatr Surg Int. 2017;33(11):1177-81. doi: <http://dx.doi.org/10.1007/s00383-017-4150-z>. PMID: 618224664.

33. McKiernan PJ, Baker AJ, Kelly DA. The frequency and outcome of biliary atresia in the UK and Ireland. Lancet. 2000;355(9197):25-9. doi: 10.1016/S0140-6736(99)03492-3. PMID: 10615887.

34. McKiernan PJ, Baker AJ, Lloyd C, Mieli-Vergani G, Kelly DA. British paediatric surveillance unit study of biliary atresia: outcome at 13 years. J Pediatr Gastroenterol Nutr. 2009;48(1):78-81. doi: 10.1097/MPG.0b013e31817d80de. PMID: 19172128.

35. Meyer RE, Liu G, Gilboa SM, Ethen MK, Aylsworth AS, Powell CM, et al. Survival of children with trisomy 13 and trisomy 18: A multi-state population-based study. Am J Med Genet A. 2016;170A(4):825-37. doi: <https://dx.doi.org/10.1002/ajmg.a.37495>. PMID: 26663415.

36. Nelson KE, Rosella LC, Mahant S, Guttmann A. Survival and Surgical Interventions for Children With Trisomy 13 and 18. JAMA. 2016;316(4):420-8. doi: 10.1001/jama.2016.9819. PMID: 27458947.

37. Nembhard WN, Salemi JL, Ethen MK, Fixler DE, Canfield MA. Mortality among infants with birth defects: Joint effects of size at birth, gestational age, and maternal race/ethnicity. Birth Defects Res A Clin Mol Teratol. 2010;88(9):728-36. doi: <https://dx.doi.org/10.1002/bdra.20696>. PMID: 20672351.

38. Nio M, Ohi R, Miyano T, Saeki M, Shiraki K, Tanaka K. Five- and 10-year survival rates after surgery for biliary atresia: A report from the Japanese Biliary Atresia Registry. J Pediatr Surg. 2003;38(7):997-1000. doi: <http://dx.doi.org/10.1016/S0022-3468%2803%2900178-7>. PMID: 36828861.

39. Oddsberg J, Lu Y, Lagergren J. Aspects of esophageal atresia in a population-based setting: incidence, mortality, and cancer risk. Pediatr Surg Int. 2012;28(3):249-57. doi: <https://dx.doi.org/10.1007/s00383-011-3014-1>. PMID: 22020495.

40. Pakarinen MP, Johansen LS, Svensson JF, Bjornland K, Gatzinsky V, Stenstrom P, et al. Outcomes of biliary atresia in the Nordic countries - a multicenter study of 158 patients during 2005-2016. J Pediatr Surg. 2018;53(8):1509-15. doi: 10.1016/j.jpedsurg.2017.08.048. PMID: 28947328.

41. Rankin J, Tennant PW, Bythell M, Pearce MS. Predictors of survival in children born with Down syndrome: a registry-based study. Pediatrics. 2012;129(6):e1373-81. doi: <https://dx.doi.org/10.1542/peds.2011-3051>. PMID: 22614780.

42. Rasmussen SA, Wong LY, Correa A, Gambrell D, Friedman JM. Survival in infants with Down syndrome, Metropolitan Atlanta, 1979-1998. J Pediatr. 2006;148(6):806-12. PMID: 16769392.

43. Risby K, Husby S, Qvist N, Jakobsen MS. High mortality among children with gastroschisis after the neonatal period: A long-term follow-up study. J Pediatr Surg. 2017;52(3):431-6. doi: <https://dx.doi.org/10.1016/j.jpedsurg.2016.08.022>. PMID: 27665495.

44. Schneuer FJ, Bell JC, Shand AW, Walker K, Badawi N, Nassar N. Five-year survival of infants with major congenital anomalies: a registry based study. Acta Paediatr Int J Paediatr. 2019;108(11):2008-18. doi: <http://dx.doi.org/10.1111/apa.14833>. PMID: 628179265.

45. Schreiber RA, Barker CC, Roberts EA, Martin SR, Alvarez F, Smith L, et al. Biliary atresia: the Canadian experience. J Pediatr. 2007;151(6):659-65, 65 e1. doi: 10.1016/j.jpeds.2007.05.051. PMID: 18035148.

46. Shin M, Kucik JE, Siffel C, Lu C, Shaw GM, Canfield MA, et al. Improved survival among children with spina bifida in the United States. J Pediatr. 2012;161(6):1132-7. doi: 10.1016/j.jpeds.2012.05.040. PMID: 22727874.

47. Siffel C, Wong LY, Olney RS, Correa A. Survival of infants diagnosed with encephalocele in Atlanta, 1979-98. Paediatr Perinat Epidemiol. 2003;17(1):40-8. PMID: 12562471.

48. Simmons K, Hashmi SS, Scheuerle A, Canfield M, Hecht JT. Mortality in babies with achondroplasia: revisited. Birth Defects Res A Clin Mol Teratol. 2014;100(4):247-9. doi: <https://dx.doi.org/10.1002/bdra.23210>. PMID: 24677650.

49. Sutton M, Daly LE, Kirke PN. Survival and disability in a cohort of neural tube defect births in Dublin, Ireland. Birth Defects Res A Clin Mol Teratol. 2008;82(10):701-9. doi: 10.1002/bdra.20498. PMID: 18803309.

50. Tennant PW, Pearce MS, Bythell M, Rankin J. 20-year survival of children born with congenital anomalies: a population-based study. Lancet. 2010;375(9715):649-56. doi: <https://dx.doi.org/10.1016/S0140-6736(09)61922-X>. PMID: 20092884.

51. Tu CG, Khurana S, Couper R, Ford AW. Kasai hepatoportoenterostomy in South Australia: a case for 'centralized decentralization'. ANZ J Surg. 2015;85(11):865-8. doi: 10.1111/ans.12522. PMID: 24529070.

52. Wang Y, Hu J, Druschel CM, Kirby RS. Twenty-five-year survival of children with birth defects in New York State: a population-based study. Birth Defects Res A Clin Mol Teratol. 2011;91(12):995-1003. doi: <https://dx.doi.org/10.1002/bdra.22858>. PMID: 21960515.

53. Wang Y, Liu G, Canfield MA, Mai CT, Gilboa SM, Meyer RE, et al. Racial/ethnic differences in survival of United States children with birth defects: A population-based study. J Pediatr. 2015;166(4):819-26.e2. doi: <http://dx.doi.org/10.1016/j.jpeds.2014.12.025>. PMID: 601970400.

54. Wildhaber BE, Majno P, Mayr J, Zachariou Z, Hohlfeld J, Schwoebel M, et al. Biliary atresia: Swiss national study, 1994-2004. J Pediatr Gastroenterol Nutr. 2008;46(3):299-307. doi: 10.1097/MPG.0b013e3181633562. PMID: 18376248.

55. Wong LY, Paulozzi LJ. Survival of infants with spina bifida: a population study, 1979-94. Paediatr Perinat Epidemiol. 2001;15(4):374-8. PMID: 11703686.
