# Supplementary material for: Long-term survival of children born with congenital anomalies: A systematic review and meta-analysis of population-based studies
Source: PLoS Med. 2020 Sep 28;17(9):e1003356. doi: 10.1371/journal.pmed.1003356 (PMC7521740; doi:10.1371/journal.pmed.1003356)
Supplement: S5 Table — (DOCX) [file pmed.1003356.s008.docx]

**S5 Table. Survival estimates by congenital anomaly type at age one to 25 years, overall and by risk factor.**

| **Congenital** |  |  | | **Survival estimates % (95% CI)** | | | | | | | | | |
| --- | --- | --- | --- | --- | --- | --- | --- | --- | --- | --- | --- | --- | --- |
| **anomaly group /subtype** | **Study and birth year** | **N deaths/live births** | | **1 year** | | **5 years** | | | **10 years** | **15 years** | **20 years** | | **25 years** |
| **All congenital anomalies** | |  | |  | |  | | |  |  |  | |  |
| ICD-9 codes 740.0-759.9 | Agha, 2006 [1]  1979-86, Canada | 3620/45,200 | | 93.4 | | 92.5 | | | ***92.3*** | **―** | **―** | | **―** |
| ICD-9 codes 740-759 | Berger, 2003 [2]  1992-98, USA | 2182/43,708 | | 95.7 | | *95.0* | | | **―** | **―** | **―** | | **―** |
| BPA-ICD-9 codes 740-759 | Dastgiri, 2003 [3], 1980-1997, Scotland | 740/6153 | | 89.11 | | 87.95 | | | **―** | **―** | **―** | | **―** |
| ICD-8 codes (740-759) | Eide, 2006 [4]  1967-79, Norway | 1169/9186 | | **―** | | **―** | | | **―** | **―** | *87.4*^a^ | | **―** |
| ICD-9 740.00-758.090 | Nembhard, 2010 [5], 1996-2003, USA | 3518/48,391 | *93.7* | | | | *92.7* | **―** | | **―** | **―** | | **―** |
|  | NHW | 1146/19620 | **―** | | | | *94.2* | **―** | | **―** | **―** | | **―** |
|  | NHB | 473/4769 | **―** | | | | *90.1* | **―** | | **―** | **―** | | **―** |
|  | Hispanic | 1899/24,002 | **―** | | | | *92.1* | **―** | | **―** | **―** | | **―** |
|  | NHW: term | 614/15,338 | **―** | | | | 96.0 (95.6-96.3) | **―** | | **―** | **―** | | **―** |
|  | NHB: term | 227/3230 | **―** | | | | 93.0 (92.0-93.8) | **―** | | **―** | **―** | | **―** |
|  | Hispanic: term | 1013/18,156 | **―** | | | | 94.4 (94.1-94.7) | **―** | | **―** | **―** | | **―** |
|  | NHW: preterm | 530/4269 | **―** | | | | 87.5 (86.5-88.5) | **―** | | **―** | **―** | | **―** |
|  | NHB: preterm | 243/1531 | **―** | | | | 84.1 (82.1-85.8) | **―** | | **―** | **―** | | **―** |
|  | Hispanic: preterm | 881/5825 | **―** | | | | 84.8 (83.9-85.7) | **―** | | **―** | **―** | | **―** |
| ICD-10 (Q00-Q99) | Tennant, 2010 [6], 1985-2003, Northern England | 1465/10,850 | | **―** | | **―** | | | **―** | **―** | 85.5 (84.8-86.3) | | **―** |
| ICD-9 codes 740-759 | Wang, 2011 [7]  1983-2006, USA | 9112/57,002 | 87.1 (86.8–87.4) | | | | 85.2 (84.9–85.5) | **―** | | 83.9 (83.6–84.2) | **―** | | 82.7 (82.4-83.1) |
| **Neural tube defects** | |  | |  | |  | | |  |  |  | |  |
| Including anencephaly | Dastgiri, 2003 [3] 1980-1997, Scotland | 40/144 | | 72.2 (64.9-79.5)^b^ | | 71.5 (63.8-79.3)^b^ | | | **―** | **―** | **―** | | **―** |
| Including anencephaly | Schneuer, 2019 [8]. 2004-2009  NSW, Australia | 34/110 | | 69.1 (60.5–77.7) | | 69.1 (60.5–77.7) | | | **―** | **―** | **―** | | **―** |
| Including anencephaly | Tennant, 2010 [6], 1985-2003, Northern England | 87/226 | | 65.0 (58.4-70.9) | | 62.8 (56.2-68.8) | | | 62.4 (55.7-68.3) | 62.4 (55.7-68.3) | 63.4 (53.4-66.7) | | **―** |
| Excluding anencephaly | Sutton, 2008 [9]  1976-87, Ireland | 313/543 | | 43.7 | | 40.8 | | | **―** | **―** | **―** | | **―** |
| **Anencephaly** | |  | |  | |  | | |  |  |  | |  |
| ICD-9 code  740.0–740.2 | Agha, 2006 [1]  1979-86, Canada | 183/ | | 4.8 | | 4.6 | | | **―** | **―** | **―** | | **―** |
|  | Tennant, 2010 [6], 1985-2003, Northern England | 17/17 | | 0.0 | | **―** | | | **―** | **―** | **―** | | **―** |
| ICD-9 740.0–740.1 | Wang, 2011 [7]  1983-2006, USA | 447/479 | | 7.3 (5.2-9.9) | | 6.8 (4.8–9.3) | | | **―** | 6.5 (4.5-9.0) | **―** | | 6.5 (4.5-9.0) |
| **Spina bifida** |  |  | |  | |  | | |  |  | |  |  |
| ICD-9 code  741.0–741.9 | Agha, 2006 [1]  1979-86 | 182/ | | 78.5 | | 75.3 | | | **―** | **―** | | **―** | **―** |
| ICD‐10 Q05 and ICD‐9 741 | Bakker, 2019 [10]  **2001-2012:**  Czech Republic | /139 | | *91.4* | | *90.0* | | | *88.6^c^* | **―** | | **―** | **―** |
|  | Malta MCAR | /28 | | *92.8* | | *92.8* | | | **―** | **―** | | **―** | **―** |
|  | Sweden | /263 | | *92.5* | | *92.1* | | | *91.7^c^* | **―** | | **―** | **―** |
|  | UK-Wales | /78 | | *91.0* | | *89.7* | | | *89.7^c^* | **―** | | **―** | **―** |
|  | USA‐Arkansas | /177 | | *87.0* | | *84.2* | | | *83.1^c^* | **―** | | **―** | **―** |
|  | USA‐Texas | /1578 | | *91.6* | | *90.5* | | | *90.1^c^* | **―** | | **―** | **―** |
|  | USA‐Utah | /213 | | *90.7* | | *90.7* | | | *90.2^c^* | **―** | | **―** | **―** |
|  | USA-Atlanta – 2001-2008 | /112 | | *95.5* | | *95.5* | | | *95.5^c^* | **―** | | **―** | **―** |
|  | Italy-Lombardy – 2003-2012 | /25 | | 100.0 | | 96.0 | | | **―** | **―** | | **―** | **―** |
| Myelomeningocele | Borgstedt-Bakke, 2017 [11], 1970-2015, Denmark | 27/187 | | ***92.4*** | | ***90.8*** | | | ***90.4*** | ***88.5*** | | ***87.7*** | **―** |
|  | 1970-79 | 16/58 | | ***84.5*** | | ***84.5*** | | | ***82.8*** | ***79.4*** | | ***79.4*** | **―** |
|  | 1980-89 | 5/39 | | ***97.5*** | | ***92.4*** | | | ***92.4*** | ***92.4*** | | ***89.8*** | **―** |
|  | 1990-2015 | 6/90 | | ***95.6*** | | ***95.6*** | | | ***94.5*** | ***92.8*** | | ***92.8*** | **―** |
| Spina bifida  (ICD-8 code 741) | Eide, 2006 [4]  1967-79, Norway | 56/113 | **―** | | | | **―** | **―** | | **―** | | *50.4*^a^ | **―** |
| Spina bifida | Schneuer, 2019 [8], 2004-2009  NSW, Australia | 11/56 | 80.4 (70.0–90.8) | | | | 80.4 (70.0–90.8) | **―** | | **―** | | **―** | **―** |
| ICD-9 741.0 and 741.9 | Shin, 2012 [12]  1997-2003, USA | 162/2259 | 92.8 (91.7-93.8) | | | | **―** | **―** | | **―** | | **―** | **―** |
|  | 1983-2003 |  | 90.8 | | | | 88.7 | 85.2 | | **―** | | **―** | **―** |
|  | 1983-1987 |  | ***87.1*** | | | | ***84.5*** | ***82.7*** | | ***80.7*** | | ***80.4*** | **―** |
|  | 1988-1992 |  | ***90.4*** | | | | ***87.6*** | ***86.7*** | | ***85.7*** | | **―** | **―** |
|  | 1993-1997 |  | ***89.9*** | | | | ***88.2*** | ***87.2*** | | **―** | | **―** | **―** |
|  | 1998-2003 |  | ***92.8*** | | | | ***90.8*** | **―** | | **―** | | **―** | **―** |
|  | 1983-2003 - White |  | ***91.6*** | | | | ***89.6*** | ***88.3*** | | ***86.9*** | | ***86.3*** | **―** |
|  | 1983-2003 – Black |  | ***87.5*** | | | | ***84.1*** | ***82.9*** | | ***80.2*** | | ***80.2*** | **―** |
|  | 1983-2003 - Hispanic |  | ***90.2*** | | | | ***88.3*** | ***87.2*** | | ***86.6*** | | ***86.6*** | **―** |
| Myelomeningocele and spinal meningocele | Sutton, 2008 [9]  1976-87, Ireland | /373 | 50.4 | | | | 47.3 | **―** | | **―** | | **―** | **―** |
| Spina bifida  ICD-10 Q05 | Tennant, 2010 [6], 1985-2003, Northern England All | 63/195 | 70.8 (63.8-76.6) | | | | 69.2 (62.2-75.2) | 68.7 (61.6-74.7) | | 68.7 (61.6-74.7) | | 66.4 (58.9-72.9) | **―** |
|  | With hydrocephalus | 51/105 | 56.2 (46.2-65.1) | | | | 54.3 (77.7-92.2) | 53.3 (43.3–62.3) | | 53.3 (43.3–62.3) | | 50.0 (39.7-59.5) | **―** |
|  | Without hydrocephalus | 12/90 | 87.8 (79.0-93.0) | | | | 86.7 (77.7-92.2) | 86.7 (77.7-92.2) | | 86.7 (77.7-92.2) | | 86.7 (77.7-92.2) | **―** |
| ICD-9 741.0, 741.9 | Wang, 2011 [7]  1983-2006, USA | 324/1999 | 88.5 (87.0–89.8) | | | | 86.4 (84.8-87.8) | **―** | | 83.8 (82.0-85.4) | | **―** | 82.2 (80.1-84.0) |
| Spina bifida without anencephalus | Wang, 2015 [13]  1999-2007, USA | 318/3903 | 91.9 (90.9-92.7) | | | | **―** | 90.2 (89.0-91.2)^d^ | | **―** | | **―** | **―** |
|  | Wong, 2001 [14]  1979-94, USA | 45/235 | 87.2 (83.1-91.6) | | | | 83.8 (79.2-88.6) | 80.9 (75.8-86.3) | | 78.4 (72.4-84.7) | | 78.4 (72.4-84.7)^a^ | **―** |
|  | 1979-83 |  | 83 (75-91) | | | | 82 (73-90) | 79 (71-88) | | **―** | | 76 (68-86**)**^a^ | **―** |
|  | 1984-88 |  | 89 (92-96) | | | | 85 (78-93) | 81 (73-90) | | **―** | | **―** | **―** |
|  | 1989-94 |  | 91 (85-98) | | | | 84 (75-94) | **―** | | **―** | | **―** | **―** |
| **Encephalocele** | |  |  | | | |  |  | |  | |  |  |
|  | Siffel, 2003 [15]  1979-98, USA | 25/83 | 72.2 (62.6-81.9) | | | | 70.8 (60.9-80.7) | **―** | | **―** | | 67.3 (55.7-78.8) | **―** |
|  | Sutton, 2008 [9]  1976-87, Ireland | /64 | 32.9 | | | | 27.3 | **―** | | **―** | | **―** | **―** |
|  | Tennant, 2010 [6], 1985-2003, Northern England | 7/14 | 64.3 (34.3-83.3) | | | | 50.0 (22.9-72.2) | 50 (22.9-72.2) | | 50 (22.9-72.2) | | **―** | **―** |
| ICD-9 742.0 | Wang, 2011 [7]  1983-2006, USA | 171/556 | 75.7 (71.9-79.1) | | | | 72.1 (68.1–75.6) | **―** | | 69.7 (65.6–73.4) | | **―** | 67.2 (62.7–71.3) |
|  | Wang, 2015 [13]  1999-2007, USA | 254/909 | 72.1 (69.0-74.9) | | | | **―** | 69.9 (66.1-73.3)^d^ | | **―** | | **―** | **―** |
| **Hydrocephalus** | |  |  | | | |  |  | |  | |  |  |
|  | Eide, 2006 [4]  1967-79, Norway | 29/59 | **―** | | | | **―** | **―** | | **―** | | *50.8*^a^ | **―** |
|  | Schneuer, 2019 [8], 2004-2009  NSW, Australia | 15/60 | 75.0 (64.0–86.0) | | | | 75.0 (64.0–86.0) | **―** | | **―** | | **―** | **―** |
|  | Tennant, 2010 [6], 1985-2003, Northern England | 32/108 | 76.9 (67.8-83.7) | | | | 75.0 (65.7-82.1) | 71.2 (61.3-79.0) | | 69.8 (59.6-77.8) | | 66.4 (54.5-75.9) | **―** |
| 742.3 | Wang, 2011 [7]  1983-2006, USA | 1314/5378 | 82.7 (81.6-83.7) | | | | 78.5 (77.4–79.6) | **―** | | 75.3 (74.1–76.5) | | **―** | 73.4 (72.1–74.7) |
| **Orofacial clefts** | |  |  | | | |  |  | |  | |  |  |
| Cleft palate and cleft lip (749.0–749.9) | Agha, 2006 [1]  1979-86, Canada | 188/ | 90.2 | | | | 88.2 | **―** | | **―** | | **―** | **―** |
| Orofacial clefts (749.0-749.9) | Bell, 2016 [16]  1980-2010, W Australia | 113/1509 | 92.5 *(91.0-93.8)* | | | | **―** | **―** | | **―** | | **―** | **―** |
| Orofacial clefts | 1980-1992 | 73/585 | **―** | | | | *87.5* *(84.5-90.0)* | **―** | | **―** | | **―** | **―** |
| Cleft lip only (BPA-ICD9 -749.10–749.19) | 1980-2010 for 1 year, 1980-2007 for 5 yrs; 1980-1992 for 20 yrs |  | 95.8 (all)  99.7 (isolated) | | | | *95.8* (all)  99.7 (isolated) | **―** | | **―** | | 97.7 (all)  100.0 (isolated) | **―** |
| Cleft lip & palate (749.20–749.27, 749.29) | 1980-2010 for 1 year,  1980-2007 for 5 yrs 1980-1992 for 20 yrs |  | 91.2 (all)  99.1 (isolated) | | | | *99.1* (isolated) | **―** | | **―** | | 84.5 (all);  98.0 (isolated) | **―** |
| Cleft palate (749.00–749.09) | 1980-2010 for 1 year, 1980-1992 for 20 yrs |  | 91.7 (all)  99.2 (isolated | | | | **―** | **―** | | **―** | | 83.5 (all);  97.2 (isolated) | **―** |
| Cleft lip with/without palate | Dastgiri, 2003 [3], 1980-97, Scotland | 5/278 | 98.2 (96.8-99.6)^b^ | | | | 98.2 (96.6-99.8)^b^ | **―** | | **―** | | **―** | **―** |
| Cleft lip | Eide, 2006 [4]  1967-79, Norway | 6/250 | **―** | | | | **―** | **―** | | **―** | | *97.6*^a^ | **―** |
| Cleft palate |  | 9/151 | **―** | | | | **―** | **―** | | **―** | | *94.0*^a^ | **―** |
| Cleft lip & palate |  | 19/357 | **―** | | | | **―** | **―** | | **―** | | *94.7*^a^ | **―** |
| Orofacial clefts | Schneuer, 2019 [8], 2004-2009  NSW, Australia | 7/575 | 99.0 (98.1–99.8) | | | | 98.8 (97.9–99.7) | **―** | | **―** | | **―** | **―** |
| Cleft lip & palate |  | 0/188 | 100.0 | | | | 100.0 | **―** | | **―** | | **―** | **―** |
| Orofacial clefts | Tennant, 2010 [6], 1985-2003, Northern England | 14/584 | 97.8 (96.2-98.7) | | | | 97.8 (96.2-98.7) | 97.6 (95.9-98.6) | | 97.6 (95.9-98.6) | | 97.6 (95.9-98.6) | **―** |
| Cleft lip |  | 1/140 | 99.3 (95.0-99.9) | | | | 99.3 (95.0-99.9) | 99.3 (95.0-99.9) | | 99.3 (95.0-99.9) | | 99.3 (95.0-99.9) | **―** |
| Cleft lip & palate |  | 5/227 | 98.2 (95.4-99.3) | | | | 98.2 (95.4-99.3) | 97.7 (94.6-99.1) | | 97.7 (94.6-99.1) | | 97.7 (94.6-99.1) | **―** |
| Cleft palate |  | 8/217 | 96.3 (92.8-98.1) | | | | 96.3 (92.8-98.1) | 96.3 (92.8-98.1) | | 96.3 (92.8-98.1) | | 96.3 (92.8-98.1) | **―** |
| Cleft lip with or without cleft palate |  | 6/367 | 98.6 (96.8-99.4) | | | | 98.6 (96.8-99.4) | 98.3 (96.3-99.2) | | 98.3 (96.3-99.2) | | 98.3 (96.3-99.2 | **―** |
| Cleft palate without cleft lip (ICD-9 749.0) | Wang, 2011 [7]  1983-2006, USA | 410/3719 | 91.0 (90.0–91.8) | | | | 89.6 (88.6–90.6) | **―** | | 88.9 (87.8–89.9) | | **―** | 88.3 (87.1-89.4) |
| Cleft lip with/without cleft palate (ICD-9 749.1-749.2) |  | 454/4691 | 91.7 (90.9–92.5) | | | | 90.8 (89.9–91.6) | **―** | | 90.2 (89.3-91.0) | | **―** | 90.0 (89.1-90.8) |
| Cleft palate without cleft lip | Wang, 2015 [13]  1999-2007, USA | 660/7356 | 91.0 (90.4-91.7) | | | | **―** | 90.3 (89.5-91.1)^d^ | | **―** | | **―** | **―** |
| Cleft lip with or without cleft palate |  | 999/11862 | 91.6 (91.1-92.1) | | | | **―** | 90.8 (90.1-91.4)^d^ | | **―** | | **―** | **―** |
| **Digestive system anomalies** | |  |  | | | |  |  | |  | |  |  |
| Oesophageal and intestinal atresias, CDH, gastroschisis, omphalocele | Garne, 2002 [17]  1980-93, Denmark | 32/90 | **―** | | | | 64.4 (*53.6-74.1*) | **―** | | **―** | | **―** | **―** |
|  | Isolated | 10/62 | ― | | | | 83.9 (*71.9-91.6*)^b^ | **―** | | **―** | | **―** | **―** |
|  | Non-isolated | 22/28 | ― | | | | 21.4 (*9.0-41.5*)^b^ | **―** | | **―** | | **―** | **―** |
| **Oesophageal atresia** | |  |  | | | |  |  | |  | |  |  |
| ICD-9 code 750.3 | Cassina, 2016 [18], 1981-2012 , NE Italy, (all) | /330 | 88.4 (84.9-91.9) | | | | **―** | **―** | | **―** | | **―** | 85.1 (80.8–89.4) |
|  | Isolated |  | 95.7 (92.6–98.8)^e^ | | | | ***94.7*** | ***94.7*** | | **―** | | **―** | 91.8 (86.9–96.7) |
|  | Non-isolated |  | 81.4 (75.5–87.3)^e^ | | | | ***85.7*** | ***85.7*** | | **―** | | **―** | 79.2 (72.9–85.5) |
|  | 1981-96 (isolated) |  | ***96.1*** | | | | ***94.6*** | ***94.6*** | | ***90.6*** | | ***90.6*** | ***90.6*** |
|  | 1997-2012 (isolated) |  | ***95.3*** | | | | ***95.3*** | ***95.3*** | | ***95.3*** | | **―** | **―** |
|  | 1981-96 (non-isolated) |  | 63.0 (49.1–76.9)^e^ | | | | ***58.7 (44.4–73.0)*** | 58.7 (44.4–73.0)^e^ | | ***58.7 (44.4–73.0)*** | | ***58.7 (44.4–73.0)*** | 58.7 (44.4–73.0) |
|  | 1997-2012 (non-isolated) |  | 88.4 (82.7–94.1)^e^ | | | | ***87.3 (81.2–93.4)*** | 87.3 (81.2–93.4)^e^ | | ***87.3 (81.2–93.4)*** | | **―** | **―** |
|  | Garne, 2002 [17], Denmark  (all 11 deaths in 12 non-isolated cases) | 11/27 | ― | | | | 59.3 (*39.0-77.0*) | ― | | ― | | ― | ― |
| ICD-7 756.21, ICD-8 750.20, 750.28, ICD-9 750D, ICD-10 Q39.0- Q39.2. | Oddsberg, 2012 [19], 1964-2007, Sweden | 227/1126 | *82.1* | | | | *80.7* | *80.6* | | *80.5* | | *80.1* |  |
|  | 1964-69 |  | *62.1* | | | | ***62.1*** | ***62.1*** | | ***62.1*** | | ***58.5*** | ***58.5*** |
|  | 1970-79 |  | ***77.2*** | | | | ***75.6*** | ***75.6*** | | ***75.2*** | | ***75.2*** | ***75.2*** |
|  | 1980-89 |  | *82.5* | | | | ***82.1*** | ***81.9*** | | ***81.9*** | | ***80.5*** | ― |
|  | 1990-99 |  | *86.1* | | | | ***85.1*** | ***85.1*** | | ***84.9*** | | ― | ― |
|  | 2000-2007 |  | ***87.8*** | | | | ***87.6*** | ― | | ― | | ― | ― |
|  | Schneuer, 2019 [8], 2004-2009  NSW, Australia | 0/51 | 100.0 | | | | 100.0 | ― | | ― | | ― | ― |
|  | Tennant, 2010 [6], 1985-2003, Northern England | 7/105 | 95.2 (88.9-98.0) | | | | 93.3 (86.5-96.8) | 93.3 (86.5-96.8) | | 93.3 (86.5-96.8) | | 93.3 (86.5-96.8) | ― |
| ICD-9 750.3 | Wang, 2011 [7]  1983-2006, USA | 336/1580 | 81.5 (79.5–83.4) | | | | 79.5 (77.4–81.4) | ― | | 78.6 (76.4–80.5) | | ― | 78.3 (76.1–80.3) |
|  | Wang, 2015 [13]  1999-2007, USA | 476/3084 | 84.6 (83.2-85.8) | | | | ― | 83.8 (82.1-85.2)^d^ | | ― | | ― | ― |
| **Anorectal malformations** | |  |  | | | |  |  | |  | |  |  |
| ICD-9/BPA 752.1-752.4, cloaca - 751.55 | Cassina, 2019 [20], 1990-2012, NE Italy, | /253 | 89.7 (85.2-92.9) | | | | ― | ― | | ― | | 86.7 (81.6–90.4) | ― |
|  | **Isolated** |  | 100.0 | | | | 100.0 | 100.0 | | ― | | ― | ― |
|  | **Non-isolated:**  Born before 2000 with BW≥ 2500g  Born before 2000 with BW<2500g  Born from 2000 with BW≥ 2500g  Born from 2000 with BW<2500g | ―  ―  ―  ― | ―  ―  ―  ― | | | | ―  ―  ―  ― | 73.3 (43.6–89.1)  23.8 (8.7–43.1)  97.9 (85.8-99.7)  68.8 (36.4-87.1) | | ―  ―  ―  ― | | ―  ―  ―  ― | ―  ―  ―  ― |
| **Anorectal atresia or stenosis** | |  |  | | | |  |  | |  | |  |  |
|  | Tennant, 2010 [6], 1985-2003, Northern England | 2/83 | 98.8 (91.8-99.8) | | | | 98.8 (91.8-99.8) | 98.8 (91.8-99.8) | | 96. 6 (86.1-99.2) | | 96. 6 (86.1-99.2) | ― |
| ICD-9 751.2 | Wang, 2011 [7]  1983-2006, USA | 374/2654 | 87.7 (86.4–88.9) | | | | 86.5 (85.2–87.8) | ― | | 85.9 (84.5–87.2) | | ― | 84.8 (83.1–86.4) |
|  | Wang, 2015 [13]  1999-2007, USA | 702/5400 | 87.0 (86.1-87.9) | | | | ― | 86.1 (85.0-87.2)^d^ | | ― | | ― | ― |
| **Hirschsprung disease** | |  |  | | | |  |  | |  | |  |  |
| ICD-7: 756.31, ICD-8: 751.39, ICD-9: 751D, ICD-10: Q431 | Löf Granström, 2017 [21], Sweden - including cases with Down syndrome | 22/739 | ***99.3 (98.7-99.8)*** | | | | ***98.3 (97.4-99.2)*** | ***98.3 (97.4-99.2)*** | | ***97.9 (96.9-99.0)*** | | ***97.7 (96.5-98.8)*** | ***97.7 (96.5-98.8)*** |
|  | Schneuer, 2019 [8], 2004-2009  NSW, Australia | 5/90 | 96.7 (93.0–100) | | | | 94.4 (89.7–99.2) | ― | | ― | | ― | ― |
|  | Tennant, 2010 [6], 1985-2003, Northern England | 4/61 | 93.4 (83.5-97.5) | | | | 93.4 (83.5-97.5) | 93.4 (83.5-97.5) | | 93.4 (83.5-97.5) | | 93.4 (83.5-97.5) | ― |
| **Biliary atresia** |  |  |  | | | |  |  | |  | |  |  |
|  | Chardot, 2013 [22], France |  |  | | | |  |  | |  | |  |  |
|  | overall survival – 1986-2009 | 228/1107 | **―** | | | | 80.8 (78.4-83.2) | 79.7 (77.2-82.2) | | 78.6 (75.9-81.3) | | 77.6 (74.5-80.7) | **―** |
|  | 1986-96 |  | **―** | | | | 72.1 (68.0-76.2) |  | |  | |  | **―** |
|  | 1997-2002 |  | **―** | | | | 88.0 (84.1-91.9) |  | |  | |  | **―** |
|  | 2003-2009 |  | **―** | | | | 88.5 (84.8-92.2) |  | |  | |  | **―** |
|  | Native liver survival (NLS) -1986-2009 | (99+542)^g^/1035 | **―** | | | | 40.0 (36.9-43.1) | 35.8 (32.7-38.9) | | 32.1 (28.8-35.4) | | 29.6 (25.7-33.5) | **―** |
|  | 1986-96 |  | **―** | | | | 38.2 (32.9-43.5) | **―** | | **―** | | **―** | **―** |
|  | 1997-2002 |  | **―** | | | | 43.1 (37.0-49.2) | **―** | | **―** | | **―** | **―** |
|  | 2003-2009 |  | **―** | | | | 39.0 (32.5-45.5) | **―** | | **―** | | **―** | **―** |
|  | Davenport, 2011 [23], 1999-2009, England &Wales | |  | | | |  |  | |  | |  |  |
|  | Overall survival | 41/443 | **―** | | | | 90 (88-93) | 89 (86-93) | | **―** | | **―** | **―** |
|  | NLS | (24+179)^g^/424 | **―** | | | | 46 (41-51) | 40 (34-46) | | **―** | | **―** | **―** |
|  | Isolated | /359 | **―** | | | | 94 (91-96)^b^ | **―** | | **―** | | **―** | **―** |
|  | Non-isolated | /84 | **―** | | | | 72 (64-83)^b^ | **―** | | **―** | | **―** | **―** |
|  | De Carvalho, 2010 [24], 1982-2008, Brazil | | | | | |  |  | |  | |  |  |
|  | Overall survival (all deaths) | 166/513 | **―** | | | | 67.6^f^ | **―** | | **―** | | **―** | **―** |
|  | Overall survival (after KP and LT) | 122/466 | **―** | | | | 73.4^f^ | **―** | | **―** | | **―** | **―** |
|  | NLS | (94+165)^g^/392 | **―** | | | | 36.8^h^ | **―** | | **―** | | **―** | **―** |
|  | De Vries, 2011 [25], 1977-1988, The Netherlands | |  | | | |  |  | |  | |  |  |
|  | Overall survival  1977-1988 | 59/104 | **―** | | | | **―** | **―** | | **―** | | 43.3 (*33.7-53.3*) | **―** |
|  | 1977-1982 | 32/49 | **―** | | | | **―** | **―** | | **―** | | 34.7 (*22.1-49.7)* | **―** |
|  | 1983-1988 | 27/55 | **―** | | | | **―** | **―** | | **―** | | 50.9 (*37.2-64.5)* | **―** |
|  | NLS  1977-1988 | (52+24)^g^/104 | **―** | | | | **―** | **―** | | **―** | | 26.9 (*18.9-36.7*) | **―** |
|  | 1977-1982 | (31+8)^g^/49 | **―** | | | | **―** | **―** | | **―** | | 20.4 (*10.7-34.8*)^b^ | **―** |
|  | 1983-1988 | (21+16)^g^/55 | **―** | | | | **―** | **―** | | **―** | | 32.7 *(21.0-46.8*)^b^ | **―** |
|  | Grizelj, 2010 [26], 1992-2006, Croatia | |  | | | |  |  | |  | |  |  |
|  | Overall survival | 7/29 | **―** | | | | *75.9 (56.1-89.0)* | *75.9 (56.1-89.0)* | | **―** | | **―** | **―** |
|  | NLS | (6+6)/28 | **―** | | | | 51.7 (40.6-62.8) | 38.8 (24.9–52.7) | | **―** | | **―** | **―** |
|  | Lampela, 2012 [27], 1987-2010, Finland | |  | | | |  |  | |  | |  |  |
|  | Overall survival | 27/72 | **―** | | | | 62.5 (*50.3-73.4*)^h^ | **―** | | **―** | | **―** | **―** |
|  | NLS | (19+25)/72 | **―** | | | | 38.9 (*27.8-51.1*)^h^ | **―** | | **―** | | **―** | **―** |
|  | Overall survival  before centralisation | 24/51 | **―** | | | | 52.9 (*38.6-66.8*)^h^ | **―** | | **―** | | **―** | **―** |
|  | Overall survival  after centralisation | 3/21 | **―** | | | | 85.7 (*62.6-96.2*)^e,h^ | **―** | | **―** | | **―** | **―** |
|  | NLS - before centralisation | (17+22)/51 | **―** | | | | 23.5 (*13.2-37.8*)^h^ | **―** | | **―** | | **―** | **―** |
|  | NLS – after centralisation | (2+3)/21 | **―** | | | | 76.2 (*52.5-90.9*)^e,h^ | **―** | | **―** | | **―** | **―** |
|  | Leonhardt, 2011 [28], 2001-2005, Germany | |  | | | |  |  | |  | |  |  |
|  | Overall survival | 31/183 | *81.9 (75.4-87.0)*^k^ | | | | **―** | **―** | | **―** | | **―** | **―** |
|  | NLS | (28+105)/167 | 20.4 *(14.7-27.4)*^k^ | | | | **―** | **―** | | **―** | | **―** | **―** |
|  | McKiernan, 2000 [29], 1993-95, UK & Ireland | |  | | | |  |  | |  | |  |  |
|  | Overall survival | 14/93 | **―** | | | | 85.0 (77.7-92.3) | ― | | **―** | | **―** | **―** |
|  | NLS | (14+33)/93 | **―** | | | | *49.5 (39.0-60.0)* | **―** | | **―** | | **―** | **―** |
|  | McKiernan, 2009 [30], UK & Ireland | |  | | | |  |  | |  | |  |  |
|  | Overall survival | 15/93 | ― | | | | ― | 83.8 (76.2-91.4)^l^ | | **―** | | **―** | **―** |
|  | NLS | (10+42)/93 | **―** | | | | **―** | 43.8 (33.3-54.1)^l^ | | **―** | | **―** | **―** |
|  | Nio, 2003 [31], Japan |  |  | | | |  |  | |  | |  |  |
|  | 1989 birth year - overall | 35/108 | **―** | | | | **―** | 66.7 | | **―** | | **―** | **―** |
|  | 1989 birth year - NLS | 51/108 | **―** | | | | **―** | 52.8 | | **―** | | **―** | **―** |
|  | 1989-94 - overall | 182/735 | **―** | | | | 75.3 | **―** | | **―** | | **―** | **―** |
|  | 1989-94- NLS | /735 | **―** | | | | 59.7 | **―** | | **―** | | **―** | **―** |
|  | Pakarinen, 2018 [32], 2005-2016, Nordic countries | |  | | | |  |  | |  | |  |  |
|  | Overall survival, including 4 untreated | 21/158 | **―** | | | | 87.3 (80.9-91.9) | 86.7 (80.2-91.4) | | **―** | | **―** | **―** |
|  | Overall survival, including treated patients only | 17/154 |  | | | | 88 (83-94) | 87 (81-93) | | **―** | | **―** | **―** |
|  | NLS | 72/154 | **―** | | | | 53 (45-62) | 45 (35-55) | | **―** | | **―** | **―** |
|  | Schreiber, 2007 [33], 1985-2002, Canada | |  | | | |  |  | |  | |  |  |
|  | 1985-2002 – overall | 81/349 |  | | | | 77 (72-92)^h^ | 75 (70-80) | | **―** | | **―** | **―** |
|  | 1985-95 – overall | 55/199 |  | | | | 74 (67-79)^h^ | **―** | | **―** | | **―** | **―** |
|  | 1996-2002 – overall | 26/150 |  | | | | 82 (75-88)^h^ | **―** | | **―** | | **―** | **―** |
|  | 1985-2002 – NLS^g^ | (81+169/349 |  | | | | 33 (28-38)^h^ | 24 (19-29) | | **―** | | **―** | **―** |
|  | 1985-95 – NLS^g^ | (55+98)/199 |  | | | | 31 (31-38)^h^ | **―** | | **―** | | **―** | **―** |
|  | 1996-2002 - NLS^g^ | (26+71)/150 |  | | | | 36 (28-45)^h^ | **―** | | **―** | | **―** | **―** |
|  | Tennant, 2010 [6], 1985-2003, Northern England | 3/14 | 85.7 (53.9-96.2) | | | | 85.7 (53.9-96.2) | **―** | | **―** | | **―** | **―** |
|  | Tu, 2015 [34], 1989-2000, Australia | |  | | | |  |  | |  | |  |  |
|  | Overall | 13/29 | ― | | | | 89.7 (71.5-97.3) | ― | | ― | | ― | ― |
|  | NLS |  | ― | | | | 55.2 (36.0-73.0) | ― | | ― | | ― | ― |
|  | Wildhaber, 2008 [35], 1994-2004, Switzerland | |  | | | |  |  | |  | |  |  |
|  | Overall | 4/48 | 91.5 (83.5-99.5)^i^ | | | | 91.5 (83.5-99.5) | 91.5 (83.5-99.5) | | ― | | ― | ― |
|  | NLS | (4+27)/48 | 40.5 (26.0-55.0)^i^ | | | | 32.7 (18.6-46.8) | ― | | ― | | ― | ― |
| **Congenital diaphragmatic hernia (CDH)** | |  |  | | | |  |  | |  | |  |  |
| ICD-9 756.6, ICD-10 Q79.0 and Q79.1 | Burgos, 2017 [36]  1987-2013, Sweden  (all fatalities) | 314/861 | *65.4 (62.1-68.5)* | | | | *63.5 (60.2-66.7)*^m^ | **―** | | **―** | | **―** | **―** |
|  | 1987-1999  (all fatalities) | 210/480 |  | | | | *56.3 (51.7-60.7)*^m^ | **―** | | **―** | | **―** | **―** |
|  | 2000-2013  (all fatalities) | 104/381 |  | | | | *72.7 (67.9-77.1)*^m^ | **―** | | **―** | | **―** | **―** |
|  | 1987-2013 |  | ***78.4*** | | | | ***77.0*** | ***76.7*** | | **―** | | **―** | **―** |
|  | 1987-1999 |  | ***72.3*** | | | | ***70.8*** | ***70.7*** | | **―** | | **―** | **―** |
|  | 2000-2013 |  | ***85.1*** | | | | ***84.1*** | ***83.4*** | | **―** | | **―** | **―** |
|  | Garne, 2002 [17]  1980-1993, Denmark | 10/17 | **―** | | | | 41.2 (*19.4-66.5*) | **―** | | **―** | | **―** | **―** |
|  | Gudbjartsson, 2008 [37], 1983-2002, Iceland | 8/23 | | | **―** | | *65.2 (42.8-82.8)*^j^ | **―** | | **―** | | **―** | **―** |
| BPA code 756.610 | Hinton, 2017 [38]  1979-2003, USA |  | | |  | |  |  | |  | |  |  |
|  | Overall survival (up to 20 y, min 3 y for all cases) | | | | | |  |  | |  | |  |  |
|  | <1988 | 22/37 | | | **―** | | **―** | 40.5 (23.4-57.6) | | **―** | | ***40.5 (23.4-57.6)*** | **―** |
|  | ≥1988 | 41/113 | | | **―** | | **―** | 58.3 (46.0-70.6) | | **―** | | **―** | **―** |
|  | White -<1988 |  | | | *43.5* | | *43.5* | *43.5* | | **―** | | **―** | **―** |
|  | White - ≥1988 |  | | | *76.8* | | *76.8* | *76.8* | | **―** | | **―** | **―** |
|  | Black -<1988 |  | | | *33.3* | | *33.3* | *33.3* | | **―** | | **―** | **―** |
|  | Black - ≥1988 |  | | | *47.5* | | *47.5* | *47.5* | | **―** | | **―** | **―** |
|  | White - CDH isolated |  | | | ***72.8*** | | ***72.8*** | ***72.8*** | | **―** | | **―** | **―** |
|  | White - CDH + another CA |  | | | ***38.8*** | | ***38.8*** | ***38.8*** | | **―** | | **―** | **―** |
|  | Black - CDH isolated |  | | | ***48.6*** | | ***48.6*** | ***48.6*** | | **―** | | **―** | **―** |
|  | Black - CDH + another CA |  | | | ***34.4*** | | ***34.4*** | ***34.4*** | | **―** | | **―** | **―** |
|  | Jaillard, 2003 [39]  1991-98, France | 34/85 | | | *60.0 (48.9-70.3*)^i^ | | **―** | **―** | | **―** | | **―** | **―** |
|  | Tennant, 2010 [6], 1985-2003, Northern England | 69/161 | | | 58.4 (50.4-65.6) | | 57.1 (49.1-64.4) | 57.1 (49.1-64.4) | | 57.1 (49.1-64.4) | | 57.1 (49.1-64.4) | **―** |
| ICD-9 756.6 | Wang, 2011 [7]  1983-2006, USA | 586/1541 | | | 63.5 (61.0–65.8) | | 62.6 (60.1–64.9) | **―** | | 62.1 (59.6-64.5) | | **―** | 61.4 (58.8-63.8) |
|  | Wang, 2015 [13]  1999-2007, USA | 1017/3248 | | | 68.7 (67.1-70.3) | | **―** | 68.0 (66.0-69.9)^d^ | | **―** | | **―** | **―** |
| **Limb anomalies** | |  |  | | | |  |  | |  | |  |  |
| **Upper limb reduction** | |  |  | | | |  |  | |  | |  |  |
|  | Tennant, 2010 [6], 1985-2003, Northern England | 1/111 | 100.0 | | | | 99.1 (93.8-99.9) | 99.1 (93.8-99.9) | | 99.1 (93.8-99.9) | | 99.1 (93.8-99.9) | **―** |
| ICD-9 755.2 | Wang, 2011 [7]  1983-2006, USA | 199/1752 | 90.7 (89.2–92.0) | | | | 89.4 (87.9–90.8) | **―** | | 89.0 (87.4–90.4) | | **―** | 87.7 (85.8–89.4) |
|  | Wang, 2015 [13]  1999-2007, USA | 387/3602 | 89.3 (88.2-90.2) | | | | **―** | 88.2 (86.9-89.4)^d^ | | **―** | | **―** | **―** |
| **Lower limb reduction** | |  |  | | | |  |  | |  | |  |  |
|  | Tennant, 2010 [6], 1985-2003, Northern England | 3/42 | 92.9 (79.5-97.6) | | | | 92.9 (79.5-97.6) | 92.9 (79.5-97.6) | | 92.9 (79.5-97.6) | | 92.9 (79.5-97.6) | **―** |
| ICD-9 755.3 | Wang, 2011 [7]  1983-2006,, USA | 136/1044 | 88.6 (86.5–90.4) | | | | 87.3 (85.2–89.2) | **―** | | 87.1 (84.9–89.0) | | **―** | 86.7 (84.4–88.6) |
|  | Wang, 2015 [13],  1999-2007, USA | 219/1913 | 88.6 (87.0-89.9) | | | | **―** | 88.2 (86.4-89.8)^d^ | | **―** | | **―** | **―** |
| **Abdominal wall defects** | |  |  | | | |  |  | |  | |  |  |
| Abdominal wall defects | Eide, 2006 [4]  1967-79, Norway | 72/206 | **―** | | | | **―** | **―** | | **―** | | *65.0*^f^ | **―** |
| **Gastroschisis** | |  |  | | | |  |  | |  | |  |  |
| surgical code DQ79.3, JAG10 | Risby, 2017 [40]  1997-2009 | 7/71 | *93.0 (83.7-97.4)* | | | | *91.5 (81.9-96.5)* | **―** | | **―** | | **―** | **―** |
|  | Schneuer, 2019 [8], 2004-2009  NSW, Australia | 9/109 | 91.7 (86.6–96.9) | | | | 91.7 (86.6–96.9) | **―** | | **―** | | **―** | **―** |
|  | Tennant, 2010 [6], 1985-2003, Northern England | 12/190 | 93.7 (89.2-96.4) | | | | 93.7 (89.2-96.4) | 93.7 (89.2-96.4) | | 93.7 (89.2-96.4) | | 93.7 (89.2-96.4) | **―** |
| ICD-9 756.73 | Wang, 2011 [7]  1983-2006, USA | 116/777 | 87.8 (85.3–89.9) | | | | 85.5 (82.8–87.8) | **―** | | 84.8 (82.0–87.2) | | **―** | 81.7 (74.0–87.3) |
|  | Wang, 2015 [13]  1999-2007, USA | 266/3698 | 92.8 (91.9-93.6) | | | | **―** | 92.1 (91.0-93.2)^d^ | | **―** | | **―** | **―** |
| **Omphalocele** | |  |  | | | |  |  | |  | |  |  |
|  | Tennant, 2010 [6], 1985-2003, Northern England | 6/47 | 87.2 (73.8-94.1) | | | | 87.2 (73.8-94.1) | 87.2 (73.8-94.1) | | 87.2 (73.8-94.1) | | 87.2 (73.8-94.1) | **―** |
| ICD-9 756.72 | Wang, 2011 [7]  1983-2006, USA | 200/639 | 69.5 (65.8–72.9) | | | | 68.8 (65.1-72.3) | **―** | | 68.6 (64.9-72.1) | | **―** | 68.6 (64.9-72.1) |
|  | Wang, 2015 [13]  1999-2007, USA | 367/1281 | 71.4 (68.8-73.7) | | | | **―** | 71.2 (68.0-74.1)^d^ | | **―** | | **―** | **―** |
| **Urinary system anomalies** | |  |  | | | |  |  | |  | |  |  |
| ICD-9 753.0–753.9 | Agha, 2006 [1]  1979-86, Canada | 451/ | 68.8 | | | | 67.2 | **―** | | **―** | | **―** | **―** |
|  | Dastgiri, 2 003 [3]  1980-1997, Scotland | 69/618 | 89.0 | | | | 88.8 | **―** | | **―** | | **―** | **―** |
| ICD-10 Q60-Q64 | Tennant, 2010 [6], 1985-2003, Northern England | 84/1258 | 93.9 (92.4-95.1) | | | | 93.5 (86.6-94.2) | 93.4 (91.9-94.6) | | 93.2 (91.6-94.5) | | 93.2 (91.6-94.5) | **―** |
| Bilateral renal agenesis |  | 21/21 | 0.0 | | | | **―** | **―** | | **―** | | **―** | **―** |
| Cystic kidney disease |  | 20/225 | 92.0 (87.6-94.9) | | | | 91.1 (86.6-94.2) | 91.1 (86.6-94.2) | | 91.1 (86.6-94.2) | | 91.1 (86.6-94.2) | **―** |
| Renal agenesis or dysgenesis – ICD-9 753.0 | Wang, 2011 [7]  1983-2006, USA | 693/1946 | 66.1 (63.9–68.1) | | | | 64.8 (62.6–66.9) | **―** | | 64.2 (62.0–66.3) | | **―** | 63.8 (61.6–66.0) |
| **Down syndrome** | |  |  | | | |  |  | |  | |  |  |
| 759.3 (ICD-8), 758.0 (ICD-9) and Q90.0, Q90.1, Q90.2 or Q90.9 (ICD-10). | Brodwall, 2018 [41], 1994-2009, Norway | 78/1251 (68/1251 – before 5 years) | 96.3 | | | | 94.2 | **―** | | **―** | | **―** | **―** |
|  | 1994-2009 – with CHD | 58/724 | 94.9 | | | | 92.0^e^ | **―** | | **―** | | **―** | **―** |
|  | 1994-2009 – without CHD | 15/527 | 98.3 | | | | 97.4^e^ | **―** | | **―** | | **―** | **―** |
|  | 1994-1999 |  | 94.2^a^ | | | | 91.8^e^ | **―** | | **―** | | **―** | **―** |
|  | 2000-2009 |  | 97.5^a^ | | | | 95.8^e^ | **―** | | **―** | | **―** | **―** |
| 758.0 (ICD-9) | Chua, 2020 [42], 1995-2014, Hong Kong | 83/1010 | 94.4 *(92.7-95.7)* | | | | *91.8*^b^ *(89.9-93.4)* | **―** | | **―** | | **―** | **―** |
|  | Dastgiri, 2003 [3]  1980-1997, Scotland | 33/210 | 87.1 (82.6-91.7)^b^ | | | | 84.3 (78.3-90.3)^b^ | **―** | | **―** | | **―** | **―** |
|  | Frid, 1999 [43]  (1973-1980), Sweden  All | 54/213 | 85.4 (*79.8-89.8*) | | | | ***77.4*** | 76.5 (*70.1-81.9*) | | 74.6 (*68.2-80.2*)^i^ | | **―** | **―** |
|  | with CHD | 48/102 | 72.5 (*62.7-80.7*)^b^ | | | | ***58.3*** | 55.9 (*45.7-65.6*)^e^ | | 52.9 (*42.8-62.8*^i^ | | **―** | **―** |
|  | without CHD | 6/111 | 97.3 (*91.7-99.3*)^b^ | | | | ***95.8*** | 95.5 (*89.3-98.3*)^e^ | | 94.6 (*88.1-97.8*)^i^ | | **―** | **―** |
|  | Glasson, 2016 [44], 1953-2010, W Australia | 245/1378 | **―** | | | | 88 (86-90) | 87 (85-89 | | **―** | | **―** | 83 (80-85) – at 30 years |
|  | 1980-2010 | 78/772 |  | | | |  |  | |  | |  |  |
|  | With CHD  (1980-2010) |  | 92 (89-94) | | | | 87 (83-90) | 87 (83-90) | |  | | 86 (82-89) | 86 (82-89) |
|  | Without CHD  (1980-2010) |  | 99 (97-100) | | | | 97 (94-98) | 96 (93-98) | |  | | 95 (93-97) | 95 (92-97) |
|  | 1980-1990 |  | 93 (89-96) | | | | 86 (81-89) | 85 (80-89) | |  | | 84 (79-88) | 82 (77-87) |
|  | 1991-2000 |  | 97 (94-99) | | | | 96 (92-98) | 95 (91-97) | |  | | 94 (90-96) | 94 (90-96) |
|  | 2001-2010 |  | 96 (92-98) | | | | 94 (90-96) | 94 (90-96) | | 94 (90-96) | | 94 (90-96) | 94 (90-96) |
|  | Halliday, 2009 [45], Australia |  |  | | | |  |  | |  | |  |  |
|  | 1988-90 | 25/236 | *94.1* | | | | 89.4 | **―** | | **―** | | **―** | **―** |
|  | 1998-2000 | 10/165 | *94.5* | | | | 93.9 | **―** | | **―** | | **―** | **―** |
|  | 1988-90 (isolated) | 2/115 | **―** | | | | 98.3 | **―** | | **―** | | **―** | **―** |
|  | 1998-2000 (isolated) | 1/76 | **―** | | | | 98.7 | **―** | | **―** | | **―** | **―** |
|  | 1988-1990 (with CHD) | 18/97 | **―** | | | | 81.4 | **―** | | **―** | | **―** | **―** |
|  | 1998-2000 (with CHD) | 9/75 | **―** | | | | 88.0 | **―** | | **―** | | **―** | **―** |
|  | Hayes, 1997 [46]  1980-89, Ireland | 63/389 | 88.2 (85-91) | | | | ***83 (79-87)*** | 83 (79-87) | | **―** | | **―** | **―** |
|  | 1980-1984 |  | 87 | | | | 82 | **―** | | **―** | | **―** | **―** |
|  | 1985-89 |  | 90 | | | | 86 | **―** | | **―** | | **―** | **―** |
|  | 1980-89 (isolated) | 22/143 | 96 | | | | 94 | **―** | | **―** | | **―** | **―** |
|  | 1980-89 (with CHD) | 41/155 | 80 | | | | 72 | **―** | | **―** | | **―** | **―** |
| BPA codes, or both BPA and ICD-9-CM, or ICD9-CM only (N Carolina and Colorado) | Kucik, 2013 [47]  1983-2003, USA  (20-year survival) | 1584/16506 | 92.9 (92.5-93.2) | | | | 91.0(90.5-91.4) | 90.7 (90.2-91.1) | | **―** | | 88.1 (87.0-89.0) | **―** |
|  | 1983-89 (20-year survival) | 334/2454 | 91.3 (90.0–92.4) | | | | 88.1 (86.8–89.3) | 87.4 (86.0–88.6) | | **―** | | 85.7 (84.1–87.1) | **―** |
|  | 1990-96 (10-year survival) | 624/5441 | 91.2 (90.5–92.0) | | | | 89.2 (88.3–90.0) | 88.4 (87.6–89.3) | | **―** | | **―** | **―** |
|  | 1997-2003 (5-year survival) | 608/8611 | 94.3 (93.8–94.8) | | | | 92.5 (91.9–93.0) | **―** | | **―** | | **―** | **―** |
|  | 1983-2003 (with CHD) | 1124/7930 | 89.6 (88.9–90.2) | | | | 86.0 (85.2–86.7) | 85.0 (84.1–85.8) | | **―** | | 82.2 (80.1–84.1) | **―** |
|  | 1983-2003 (without CHD) | 460/8576 | 96.0 (95.5–96.4) | | | | 95.0 (94.5–95.5) | 94.4 (93.8–94.9) | | **―** | | 93.0 (92.0–93.9) | **―** |
|  | Leonard, 2000 [48], 1980-96, W Australia | /440 | 91.7 (88.7-94.0) | | | | 87.0 (83.0-89.0) | 85.0 (81.0-89.0) | | **―** | | **―** | **―** |
|  | 1980-85 |  | 89 | | | | 80 (72-86)^e^ | 79 | | **―** | | **―** | **―** |
|  | 1986-90 |  | 92 | | | | 86 (79-91)^e^ | 85 | | **―** | | **―** | **―** |
|  | 1991-96 |  | 94 | | | | 93 (88-96)^e^ | **―** | | **―** | | **―** | **―** |
|  | 1980-96 (with CHD) |  | 88.0 (82.4-81.9) | | | | 78 (71-83)^e^ | **―** | | **―** | | **―** | **―** |
|  | 1980-96 (without CHD) |  | 94.4 (90.7-96.6) | | | | 93 (89-96)^b^ | **―** | | **―** | | **―** | **―** |
| Q900-Q902 | Rankin, 2012 [49],^n,^ Northern England  1985-2003 -All | 111/669 | 88.3 (85.7–90.6) | | | | 84.6 (81.6–87.1) | 83.9 (80.9–86.5) | | **―** | | 72.9 (79.7–85.7) | **―** |
|  | 1985-2003 (no additional CA) | 25/347 | 95.4 (92.6–97.2) | | | | 93.7 (90.5–95.8) | 93.3 (90.1–95.5) | | **―** | | 93.3 (90.1–95.5) | **―** |
|  | 1985-2003 (with CHD only) | 65/260 | 82.7 (77.5–86.8) | | | | 76.9 (71.3–81.6) | 75.6 (69.8–80.4) | | **―** | | 74.7 (68.8–79.7) | **―** |
|  | 1985–1990 | 54/235 | 86.0 (80.8–89.8) | | | | 79.2 (73.4–83.8) | 78.3 (72.5–83.0) | | **―** | | 77.5 (71.6–82.3) | **―** |
|  | 1991–1996 | 36/193 | 83.9 (78.0–88.4) | | | | 82.4 (76.2–87.1) | 81.9 (75.7–86.6) | | **―** | | 80.6 | **―** |
|  | 1997–2003 | 21/241 | 94.2 (90.4–96.5) | | | | 91.7 (87.4–94.6) | 91.2 (86.8–94.2) | | **―** | | 90.7 | **―** |
| ICD-9-CM (758.000-758.090) | Rasmussen, 2006 [50], 1979-98, USA | 70/645 | 92.9 (90.9-94.9) | | | | ***89.9 (87.3-92.1)*** | 88.6 (85.0-92.2**)** | | **―** | | 87.4 (84.3-90.5) | **―** |
|  | 1979-1998 (with CHD) | 45/266 | ***88.9*** | | | | ***84.1*** | ***82.8*** | | **―** | | 78.9 (71.5–86.2) | **―** |
|  | 1979-1998 (without CHD) | 25/379 | ***95.5*** | | | | ***93.9*** | ***93.2*** | | **―** | | 92.6 (89.7–95.5) | **―** |
|  | 1979-1998 (White) | 24/355 | ***95.5*** | | | | ***94.4*** | ***93.3*** | | **―** | | 91.8 (88.2–95.4) | **―** |
|  | 1979-1998 (Black) | 41/243 | ***88.9*** | | | | ***84.2*** | ***83.5*** | | **―** | | 80.9 (75.3–86.7) | **―** |
| ICD-9 758.0 | Wang, 2011 [7]  1983-2006, USA | 754/6819 | 92.0 (91.3-92.6) | | | | 89.9 (89.1-90.6) | **―** | | 88.9 (88.1-89.7) | | **―** | 87.5 (86.5-88.5) |
|  | Wang, 2015 [13]  1999-2007, USA | 944/15,939 | \| 94.1 (93.7-94.4) \| \| --- \| | | | | **―** | 92.8 (92.3-93.2)^d^ | | **―** | | **―** | **―** |
| **Trisomy 13** |  |  |  | | | |  |  | |  | |  |  |
|  | Meyer, 2016 [51], USA | 625/693 | 11.5 (9.3-14.1) | | | | 9.7 (7.2-12.5) | **―** | | **―** | | **―** | **―** |
| ICD-9, 758.1 or ICD-10, Q91.4-Q91.7 | Nelson, 2016 [52], 1991-2012, Canada | /174 | 19.8 (14.2-26.1) | | | | 15 (10-21) | 12.9 (8.4-18.5) | | **―** | | **―** | **―** |
|  | Tennant, 2010 [6], 1985-2003, Northern England | 26/29 | 13.8 (4.4-28.6) | | | | **―** ^o^ | **―** | | **―** | | **―** | **―** |
| ICD-9 758.1 | Wang, 2011 [7]  1983-2006 | 437/525 | 21.3 (17.9-24.9) | | | | 18.4 (15.3–21.9) | **―** | | 16.2 (13.0–19.7) | |  | 15.2 (12.0–18.8) |
| **Trisomy 18** |  |  |  | | | |  |  | |  | |  |  |
|  | Meyer, 2016 [51], USA | 984/1,113 | 13.4 (11.5-15.5) | | | | 12.3 (10.1-14.8) | **―** | | **―** | | **―** | **―** |
| ICD-9, 758.2 or ICD-10, Q91.0-Q91.3 | Nelson, 2016 [52], 1991-2012 Canada | /254 | 12.6 (8.9-17.1) | | | | 11 (8-16) | 9.8 (6.4-14.0) | | **―** | | **―** | **―** |
|  | Tennant, 2010 [6], 1985-2003, Northern England | 62/63 | 1.6 (0.1-7.5) | | | | **―** ^o^ | **―** | | **―** | | **―** | **―** |
| ICD-9 758.2 | Wang, 2011 [7]  1983-2006, USA | 667/773 | 18.8 (16.1-21.6) | | | | 15.2 (12.8-17.8) | **―** | | 13.2 (10.9–15.8) | | **―** | 12.3 (9.8–15.1) |
| **Skeletal dysplasia** | |  | : | | | |  |  | |  | |  |  |
| Osteogenesis Imperfecta  ICD-10 Q78.0 | Folkestad, 2016 [53], 1977-2012, Denmark | 24/366 (up to 20 years) | *94.8 (91.8-96.8)* | | | | *94.8 (91.8-96.8)* | **―** | | **―** | | *91.6 (88.2-94.2)* | **―** |
| Skeletal dysplasia | Schneuer, 2019 [8], 2004-2009  NSW, Australia | 15/75 | 80.0 (70.9–89.1) | | | | 80.0 (70.9–89.1) | **―** | | **―** | | **―** | **―** |
| Achondroplasia  BPA code 756.430 | Simmons, 2014 [54], 1996-2005m USA | 4/106 | *96.2 (90.1-98.8)* | | | | *96.2 (90.1-98.8)*^i^ | **―** | | **―** | | **―** | **―** |
| Achondroplasia/  Hypochondroplasia | Tennant, 2010 [6], 1983-2003, Northern England | 2/22 | 95.5 (71.9-99.4) | | | | 90.9 (68.3-97.7) | 90.9 (68.3-97.7) | | 90.9 (68.3-97.7) | | **―** | **―** |
| **Prader-Willi syndrome** | |  |  | | | |  |  | |  | |  |  |
|  | Lionti, 2012 [55]  1950-2010, Australia | 15/163 (to 35 years) | ***98.6 (95.2-99.7)*** | | | | ***98.6 (95.2-99.7)*** | 97 (93-99) | | ***96.3 (91.1-98.4)*** | | 94 (88-97) | ***89.4 (80.8-94.5*** |
| ICD-10 Q87.1 | Tennant, 2010 [6], 1983-2003, Northern England | 1/10 | 100.0 | | | | 90.0 (47.3-98.5) | 90.0 (47.3-98.5) | | **―** | | **―** | **―** |

**Note:**

Congenital anomaly subtypes were presented within the major congenital anomaly groups according to the EUROCAT classification [56].

Estimates (or 95% CI) in italics were not reported in the article but were estimated from the raw data provided and in italics and bold were extracted from Kaplan-Meier or actuarial survival curves. For calculation of 95% CIs, we used the efficient-score method (corrected for continuity) described by Newcombe, 1998 [57], based on the procedure outlined by Wilson, 1927 [58].

^a^18-year survival values; ^b^Provided by authors on request or confirmed by authors; ^c^survival at ≥5 years reported; ^d^8-year survival values; ^e^*p* values <0.05; ^f^overall survival reported, including all deaths (also without operation or liver transplantation), without specifying age at survival; ^g^deaths and secondary liver transplantation used in calculation of native liver survival; ^h^4-year survival values; ^i^14.5-year survival values; ^j^3-year survival values, ^k^2-year survival values, ^l^13-year survival values; ^m^overall survival (beyond one year of age) for all live births reported;  ^n^this article (Rankin, 2012 [49]) was included despite being a subset of the larger study analysing all types of congenital anomalies (Tennant et al [6]) because it reported survival by year period and explored predictors of survival. To avoid duplication in reporting, survival for Down syndrome from Tennant et al [6] was included neither in the tables of this review nor in the meta-analysis; ^o^ survival not reported as <5 cases at risk at the end of the time period.

CA, major congenital anomaly; KP, Kasai hepatoportoenterostomy; LT, liver transplantation; NLS, native liver survival, NSW, New South Wales.

References

1. Agha MM, Williams JI, Marrett L, To T, Dodds L. Determinants of survival in children with congenital abnormalities: a long-term population-based cohort study. Birth Defects Res A Clin Mol Teratol. 2006;76(1):46-54. PMID: 16397887.

2. Berger KH, Zhu BP, Copeland G. Mortality throughout early childhood for Michigan children born with congenital anomalies, 1992-1998. Birth Defects Res A Clin Mol Teratol. 2003;67(9):656-61. PMID: 14703790.

3. Dastgiri S, Gilmour WH, Stone DH. Survival of children born with congenital anomalies. Arch Dis Child. 2003;88(5):391-4. PMID: 12716706.

4. Eide MG, Skjaerven R, Irgens LM, Bjerkedal T, Oyen N. Associations of birth defects with adult intellectual performance, disability and mortality: population-based cohort study. Pediatr Res. 2006;59(6):848-53. PMID: 16641211.

5. Nembhard WN, Salemi JL, Ethen MK, Fixler DE, Canfield MA. Mortality among infants with birth defects: Joint effects of size at birth, gestational age, and maternal race/ethnicity. Birth Defects Res A Clin Mol Teratol. 2010;88(9):728-36. doi: <https://dx.doi.org/10.1002/bdra.20696>. PMID: 20672351.

6. Tennant PW, Pearce MS, Bythell M, Rankin J. 20-year survival of children born with congenital anomalies: a population-based study. Lancet. 2010;375(9715):649-56. doi: <https://dx.doi.org/10.1016/S0140-6736(09)61922-X>. PMID: 20092884.

7. Wang Y, Hu J, Druschel CM, Kirby RS. Twenty-five-year survival of children with birth defects in New York State: a population-based study. Birth Defects Res A Clin Mol Teratol. 2011;91(12):995-1003. doi: <https://dx.doi.org/10.1002/bdra.22858>. PMID: 21960515.

8. Schneuer FJ, Bell JC, Shand AW, Walker K, Badawi N, Nassar N. Five-year survival of infants with major congenital anomalies: a registry based study. Acta Paediatr Int J Paediatr. 2019;108(11):2008-18. doi: <http://dx.doi.org/10.1111/apa.14833>. PMID: 628179265.

9. Sutton M, Daly LE, Kirke PN. Survival and disability in a cohort of neural tube defect births in Dublin, Ireland. Birth Defects Res A Clin Mol Teratol. 2008;82(10):701-9. doi: 10.1002/bdra.20498. PMID: 18803309.

10. Bakker MK, Kancherla V, Canfield MA, Bermejo-Sanchez E, Cragan JD, Dastgiri S, et al. Analysis of Mortality among Neonates and Children with Spina Bifida: An International Registry-Based Study, 2001-2012. Paediatr Perinat Epidemiol. 2019;33(6):436-48. doi: 10.1111/ppe.12589. PMID: 31637749.

11. Borgstedt-Bakke JH, Fenger-Gron M, Rasmussen MM. Correlation of mortality with lesion level in patients with myelomeningocele: a population-based study. J Neurosurg Pediatrics. 2017;19(2):227-31. doi: <https://dx.doi.org/10.3171/2016.8.PEDS1654>. PMID: 27911247.

12. Shin M, Kucik JE, Siffel C, Lu C, Shaw GM, Canfield MA, et al. Improved survival among children with spina bifida in the United States. J Pediatr. 2012;161(6):1132-7. doi: 10.1016/j.jpeds.2012.05.040. PMID: 22727874.

13. Wang Y, Liu G, Canfield MA, Mai CT, Gilboa SM, Meyer RE, et al. Racial/ethnic differences in survival of United States children with birth defects: A population-based study. J Pediatr. 2015;166(4):819-26.e2. doi: <http://dx.doi.org/10.1016/j.jpeds.2014.12.025>. PMID: 601970400.

14. Wong LY, Paulozzi LJ. Survival of infants with spina bifida: a population study, 1979-94. Paediatr Perinat Epidemiol. 2001;15(4):374-8. PMID: 11703686.

15. Siffel C, Wong LY, Olney RS, Correa A. Survival of infants diagnosed with encephalocele in Atlanta, 1979-98. Paediatr Perinat Epidemiol. 2003;17(1):40-8. PMID: 12562471.

16. Bell JC, Nassar N, Bower C, Turner RM, Raynes-Greenow C. Long-term survival for infants born with orofacial clefts in Western Australia. Birth Defects Res A Clin Mol Teratol. 2016;106(3):172-7. doi: <https://dx.doi.org/10.1002/bdra.23473>. PMID: 26663708.

17. Garne E, Rasmussen L, Husby S. Gastrointestinal malformations in Funen county, Denmark - epidemiology, associated malformations, surgery and mortality. Eur J Pediatr Surg. 2002;12(2):101-6. PMID: 12015653.

18. Cassina M, Ruol M, Pertile R, Midrio P, Piffer S, Vicenzi V, et al. Prevalence, characteristics, and survival of children with esophageal atresia: A 32-year population-based study including 1,417,724 consecutive newborns. Birth Defects Res A Clin Mol Teratol. 2016;106(7):542-8. doi: <https://dx.doi.org/10.1002/bdra.23493>. PMID: 26931365.

19. Oddsberg J, Lu Y, Lagergren J. Aspects of esophageal atresia in a population-based setting: incidence, mortality, and cancer risk. Pediatr Surg Int. 2012;28(3):249-57. doi: <https://dx.doi.org/10.1007/s00383-011-3014-1>. PMID: 22020495.

20. Cassina M, Fascetti Leon F, Ruol M, Chiarenza SF, Scire G, Midrio P, et al. Prevalence and survival of patients with anorectal malformations: A population-based study. J Pediatr Surg. 2019;54(10):1998-2003. doi: <https://dx.doi.org/10.1016/j.jpedsurg.2019.03.004>. PMID: 30935729.

21. Löf Granström A, Wester T. Mortality in Swedish patients with Hirschsprung disease. Pediatr Surg Int. 2017;33(11):1177-81. doi: <http://dx.doi.org/10.1007/s00383-017-4150-z>. PMID: 618224664.

22. Chardot C, Buet C, Serinet MO, Golmard JL, Lachaux A, Roquelaure B, et al. Improving outcomes of biliary atresia: French national series 1986-2009. J Hepatol. 2013;58(6):1209-17. doi: 10.1016/j.jhep.2013.01.040. PMID: 23402746.

23. Davenport M, Ong E, Sharif K, Alizai N, McClean P, Hadzic N, et al. Biliary atresia in England and Wales: results of centralization and new benchmark. J Pediatr Surg. 2011;46(9):1689-94. doi: 10.1016/j.jpedsurg.2011.04.013. PMID: 21929975.

24. De Carvalho E, Santos JL, Silveira TR, Kieling CO, Silva LR, Porta G, et al. Biliary atresia: the Brazilian experience. J Pediatr (Rio J). 2010;86(6):473-9. doi: 10.2223/JPED.2054. PMID: 21140036.

25. de Vries W, Homan-Van der Veen J, Hulscher JB, Hoekstra-Weebers JE, Houwen RH, Verkade HJ, et al. Twenty-year transplant-free survival rate among patients with biliary atresia. Clin Gastroenterol Hepatol. 2011;9(12):1086-91. doi: 10.1016/j.cgh.2011.07.024. PMID: 21820397.

26. Grizelj R, Vukovic J, Novak M, Batinica S. Biliary atresia: the Croatian experience 1992-2006. Eur J Pediatr. 2010;169(12):1529-34. doi: 10.1007/s00431-010-1266-8. PMID: 20669030.

27. Lampela H, Ritvanen A, Kosola S, Koivusalo A, Rintala R, Jalanko H, et al. National centralization of biliary atresia care to an assigned multidisciplinary team provides high-quality outcomes. Scand J Gastroenterol. 2012;47(1):99-107. doi: <https://dx.doi.org/10.3109/00365521.2011.627446>. PMID: 22171974.

28. Leonhardt J, Kuebler JF, Leute PJ, Turowski C, Becker T, Pfister ED, et al. Biliary atresia: lessons learned from the voluntary German registry. Eur J Pediatr Surg. 2011;21(2):82-7. doi: <https://dx.doi.org/10.1055/s-0030-1268476>. PMID: 21157692.

29. McKiernan PJ, Baker AJ, Kelly DA. The frequency and outcome of biliary atresia in the UK and Ireland. Lancet. 2000;355(9197):25-9. doi: 10.1016/S0140-6736(99)03492-3. PMID: 10615887.

30. McKiernan PJ, Baker AJ, Lloyd C, Mieli-Vergani G, Kelly DA. British paediatric surveillance unit study of biliary atresia: outcome at 13 years. J Pediatr Gastroenterol Nutr. 2009;48(1):78-81. doi: 10.1097/MPG.0b013e31817d80de. PMID: 19172128.

31. Nio M, Ohi R, Miyano T, Saeki M, Shiraki K, Tanaka K. Five- and 10-year survival rates after surgery for biliary atresia: A report from the Japanese Biliary Atresia Registry. J Pediatr Surg. 2003;38(7):997-1000. doi: <http://dx.doi.org/10.1016/S0022-3468%2803%2900178-7>. PMID: 36828861.

32. Pakarinen MP, Johansen LS, Svensson JF, Bjornland K, Gatzinsky V, Stenstrom P, et al. Outcomes of biliary atresia in the Nordic countries - a multicenter study of 158 patients during 2005-2016. J Pediatr Surg. 2018;53(8):1509-15. doi: 10.1016/j.jpedsurg.2017.08.048. PMID: 28947328.

33. Schreiber RA, Barker CC, Roberts EA, Martin SR, Alvarez F, Smith L, et al. Biliary atresia: the Canadian experience. J Pediatr. 2007;151(6):659-65, 65 e1. doi: 10.1016/j.jpeds.2007.05.051. PMID: 18035148.

34. Tu CG, Khurana S, Couper R, Ford AW. Kasai hepatoportoenterostomy in South Australia: a case for 'centralized decentralization'. ANZ J Surg. 2015;85(11):865-8. doi: 10.1111/ans.12522. PMID: 24529070.

35. Wildhaber BE, Majno P, Mayr J, Zachariou Z, Hohlfeld J, Schwoebel M, et al. Biliary atresia: Swiss national study, 1994-2004. J Pediatr Gastroenterol Nutr. 2008;46(3):299-307. doi: 10.1097/MPG.0b013e3181633562. PMID: 18376248.

36. Burgos CM, Frenckner B. Addressing the hidden mortality in CDH: A population-based study. J Pediatr Surg. 2017;52(4):522-5. doi: <https://dx.doi.org/10.1016/j.jpedsurg.2016.09.061>. PMID: 27745705.

37. Gudbjartsson T, Gunnarsdottir A, Topan CZ, Larsson LT, Rosmundsson T, Dagbjartsson A. Congenital diaphragmatic hernia: Improved surgical results should influence abortion decision making. Scand J Surg. 2008;97(1):71-6. PMID: 351449967.

38. Hinton CF, Siffel C, Correa A, Shapira SK. Survival Disparities Associated with Congenital Diaphragmatic Hernia. Birth Defects Res A Clin Mol Teratol. 2017;109(11):816-23. doi: 10.1002/bdr2.1015. PMID: 28398654.

39. Jaillard SM, Pierrat V, Dubois A, Truffert P, Lequien P, Wurtz AJ, et al. Outcome at 2 years of infants with congenital diaphragmatic hernia: a population-based study. Ann Thorac Surg. 2003;75(1):250-6. PMID: 12537224.

40. Risby K, Husby S, Qvist N, Jakobsen MS. High mortality among children with gastroschisis after the neonatal period: A long-term follow-up study. J Pediatr Surg. 2017;52(3):431-6. doi: <https://dx.doi.org/10.1016/j.jpedsurg.2016.08.022>. PMID: 27665495.

41. Brodwall K, Greve G, Leirgul E, Klungsøyr K, Holmstrøm H, Vollset SE, et al. The five-year survival of children with Down syndrome in Norway 1994–2009 differed by associated congenital heart defects and extracardiac malformations. Acta Paediatr Int J Paediatr. 2018;107(5):845-53. doi: 10.1111/apa.14223.

42. Chua GT, Tung KTS, Wong ICK, Lum TYS, Wong WHS, Chow CB, et al. Mortality Among Children with Down syndrome in Hong Kong: A Population-Based Cohort Study from Birth. J Pediatr. 2020;218:138-45. doi: <http://dx.doi.org/10.1016/j.jpeds.2019.11.006>. PMID: 2004554180.

43. Frid C, Drott P, Lundell B, Rasmussen F, Anneren G. Mortality in Down's syndrome in relation to congenital malformations. J Intellect Disabil Res. 1999;43 ( Pt 3):234-41. PMID: 10392609.

44. Glasson EJ, Jacques A, Wong K, Bourke J, Leonard H. Improved Survival in Down Syndrome over the Last 60 Years and the Impact of Perinatal Factors in Recent Decades. J Pediatr. 2016;169:214-20.e1. doi: <https://dx.doi.org/10.1016/j.jpeds.2015.10.083>. PMID: 26651430.

45. Halliday J, Collins V, Riley M, Youssef D, Muggli E. Has prenatal screening influenced the prevalence of comorbidities associated with Down syndrome and subsequent survival rates? Pediatrics. 2009;123(1):256-61. doi: <https://dx.doi.org/10.1542/peds.2007-2840>. PMID: 19117890.

46. Hayes C, Johnson Z, Thornton L, Fogarty J, Lyons R, O'Connor M, et al. Ten-year survival of Down syndrome births. Int J Epidemiol. 1997;26(4):822-9. PMID: 9279615.

47. Kucik JE, Shin M, Siffel C, Marengo L, Correa A. Trends in survival among children with down syndrome in 10 regions of the united states. Pediatrics. 2013;131(1):e27-e36. doi: <http://dx.doi.org/10.1542/peds.2012-1616>. PMID: 368184663.

48. Leonard S, Bower C, Petterson B, Leonard H. Survival of infants born with Down's syndrome: 1980-96. Paediatr Perinat Epidemiol. 2000;14(2):163-71. PMID: 10791661.

49. Rankin J, Tennant PW, Bythell M, Pearce MS. Predictors of survival in children born with Down syndrome: a registry-based study. Pediatrics. 2012;129(6):e1373-81. doi: <https://dx.doi.org/10.1542/peds.2011-3051>. PMID: 22614780.

50. Rasmussen SA, Wong LY, Correa A, Gambrell D, Friedman JM. Survival in infants with Down syndrome, Metropolitan Atlanta, 1979-1998. J Pediatr. 2006;148(6):806-12. PMID: 16769392.

51. Meyer RE, Liu G, Gilboa SM, Ethen MK, Aylsworth AS, Powell CM, et al. Survival of children with trisomy 13 and trisomy 18: A multi-state population-based study. Am J Med Genet A. 2016;170A(4):825-37. doi: <https://dx.doi.org/10.1002/ajmg.a.37495>. PMID: 26663415.

52. Nelson KE, Rosella LC, Mahant S, Guttmann A. Survival and Surgical Interventions for Children With Trisomy 13 and 18. JAMA. 2016;316(4):420-8. doi: 10.1001/jama.2016.9819. PMID: 27458947.

53. Folkestad L, Hald JD, Canudas-Romo V, Gram J, Hermann AP, Langdahl B, et al. Mortality and Causes of Death in Patients With Osteogenesis Imperfecta: A Register-Based Nationwide Cohort Study. J Bone Miner Res. 2016;31(12):2159-66. doi: <https://dx.doi.org/10.1002/jbmr.2895>. PMID: 27345018.

54. Simmons K, Hashmi SS, Scheuerle A, Canfield M, Hecht JT. Mortality in babies with achondroplasia: revisited. Birth Defects Res A Clin Mol Teratol. 2014;100(4):247-9. doi: <https://dx.doi.org/10.1002/bdra.23210>. PMID: 24677650.

55. Lionti T, Reid SM, Rowell MM. Prader-Willi syndrome in Victoria: mortality and causes of death. J Paediatr Child Health. 2012;48(6):506-11. doi: <https://dx.doi.org/10.1111/j.1440-1754.2011.02225.x>. PMID: 22697408.

56. European Surveillance of Congenital Anomalies. Chapter 3.3: EUROCAT Subgroups of Congenital Anomalies (Version 2014). EUROCAT Guide 14 and reference documents. Newtownabbey, UK: EUROCAT Central Registry; 2013.

57. Newcombe RG. Two-Sided Confidence Intervals for the Single Proportion: Comparison of Seven Methods. Stat Med. 1998;17(8):857-72.

58. Wilson EB. Probable Inference, the Law of Succession, and Statistical Inference. J Amer Stat Assoc. 1927;22:209-12
