# Supplementary material for: Long-term survival of children born with congenital anomalies: A systematic review and meta-analysis of population-based studies
Source: PLoS Med. 2020 Sep 28;17(9):e1003356. doi: 10.1371/journal.pmed.1003356 (PMC7521740; doi:10.1371/journal.pmed.1003356)
Supplement: S6 Table — (DOCX) [file pmed.1003356.s009.docx]

# **S6 Table. Predictors of survival/mortality in the included studies that explored risk factors associated with survival at different age points, including infancy (n=35), by congenital anomaly group/subtype**

| **Congenital anomaly group/subtype** | | **Study** | **Risk factors** | **Category** | **Unadjusted**  **OR/HR/RR (95% CI)** | **Adjusted**  **aOR/aHR**  **(95% CI)** | **Factors adjusted for** | |
| --- | --- | --- | --- | --- | --- | --- | --- | --- |
| **All congenital anomalies** | | |  |  |  |  |  | |
|  | | Agha, 2006 [1] | Number of anomalies | 1  2  3  ≥4 | RR for risk of death at 10 years  1.0 (ref)  3.3 (3.1-3.7)  6.8 (6.2-7.6)  13.8 (12.7–15.0) | **―** | **―** | |
|  | |  | Number of previous stillbirths | 0  1  ≥2 | 1.0 (ref)  1.12 (1.04–1.2)  1.29 (1.2–1.4) | **―** | **―** | |
|  | |  | Weeks of gestation | ≤37  38-40  >40 | 1.1 (0.99-1.2)  1.0 (ref)  1.2 (1.1-1.3) | **―** | **―** | |
|  | |  | Birth weight (g) | ≤2500  2501-3000  3001-4000  >4000 | 2.2 (2.0-2.4)^c^  1.0 (ref)  0.6 (0.5-0.7)  0.5 (0.4-0.6) | **―** | **―** | |
|  | |  | Maternal age (y) | ≤20  21-34  ≥35 | 1.2 (1.03-1.3)^c^  1.0 (ref)  0.9 (0.8-1.1) | **―** | **―** | |
|  | | Berger, 2003 [2] | Race | White  Black | 7-year HR  1.0 (ref)  1.5 (1.4-1.6)^c^ | 7-year aHR  1.0 (ref)  1.0 (0.9-1.1)^g^ | Birth weight, sex, mother’s age, mother’s education, number of organ systems affected | |
|  | | Nembhard, 2010 [3] | Ethnicity | NHW:  NHB  Hispanic | 5-year HR  1.0 (ref)  1.3 (1.6-1.9)^f^  1.4 (1.3-1.5)^c^ | 5-year aHR  1.0 (ref)  1.5 (1.4-1.7)^f^  1.1 (1.01-1.2) | Maternal age, maternal education, infant sex, border county, and number of birth defects |  |
|  | |  | Size at birth | AGA  SGA  LGA | 1.0 (ref)  2.6 (2.4-2.8)^f^  0.6 (0.5-0.7)^f^ | 1.0 (ref)  2.1 (1.9-2.2)^f^  0.6 (0.5-0.7)^f^ | Maternal age, maternal education, infant sex, border county, and number of birth defects |  |
|  | |  | Gestational age (weeks) | ≥37 weeks  <37 weeks | 1.0 (ref)  3.0 (2.8-3.2)^f^ | 1.0 (ref)  2.7 (2.5-2.9)^f^ | Maternal age, maternal education, infant sex, border county, and number of birth defects |  |
|  | | Schneuer, 2019 [4] | Gestational age (weeks) | ≥37 weeks  <37 weeks | 5-year survival  95.6 (95.3-96.3)  79.4 (77.5–81.4)^e^ | n/a | n/a |  |
|  | | Wang, 2011 [5] | Gestational age (weeks), birthweight (g)^a^ | ≥37, 2500–3999  <37, <1500  <37, 1500-2499  <37, 2500-3999  <37, ≥4000  ≥37, <1500  ≥37, 1500-2499  ≥37, ≥4000 | **―** | 25-year aHR  1.0 (ref)  4.9 (4.6-5.2)^c^  2.7 (2.6-2.9)^c^  1.5 (1.4-1.6)^c^  1.8 (1.3-2.5)^c^  4.4 (3.7-5.2)^c^  2.9 (2.7-3.1)^c^  0.7 (0.6-0.8)^g^ | Infant sex, plurality, number of CAs, parity, maternal age, ethnicity, nativity and education, birth year period |  |
|  | |  | Plurality^a^ | Single  Multiple | **―** | 1.0 (ref)  0.8 (0.75-0.9)^c^ | Infant sex, birth weight, gestational age, number of CAs, parity, maternal age, ethnicity, nativity and education, birth year period |  |
|  | |  | Number of CAs^a^ | Isolated  Non-isolated | **―** | 1.0 (ref)  2.8 (2.7-3.0)^c^ | Infant sex, birth weight, gestational age, plurality, number of CAs, parity, maternal age, ethnicity, nativity and education, birth year period |  |
|  | |  | Maternal age (years) ^a^ | ≤19  20-24  25-29  30-34  ≥35 | **―** | 1.2 (1.1-1.3)^c^  1.1 (1.03-1.2)^c^  1.05 (1.0-1.1)^g^  1.0 (ref)  1.0 (0.9-1.0)^g^ | Infant sex, birth weight, gestational age, plurality, number of CAs, parity, maternal ethnicity, nativity and education, birth year period |  |
|  | |  | Maternal nativity^a^ | U.S. born  Others | **―** | 1.0 (ref)  1.1 (1.03-1.15)^c^ | Infant sex, birth weight, gestational age, plurality, number of CAs, parity, maternal age, ethnicity and education, birth year period |  |
|  | |  | Parity^a^ | 0  ≥1 | **―** | 1.0 (ref)  1.2 (1.1-1.2)^c^ | Infant sex, birth weight, gestational age, plurality, number of CAs, maternal age, ethnicity, nativity and education, birth year period |  |
|  | |  | Birth year period | 1982-1988  1989-1994  1995-2000  2001-2006^a^ | **―** | 1.8 (1.6-1.9)^c^  1.5 (1.4-1.6)^c^  1.3 (1.2-1.4)^c^  1.0 (ref) | Infant sex, birth weight, gestational age, plurality, number of CAs, parity, maternal age, ethnicity, nativity and education. |  |
| **Neural tube defects** | | |  |  |  |  |  |  |
| Excluding anencephaly | | Sutton, 2008 [6] | Presence of hydrocephalus at birth^a^ | Yes  No | 1-year survival  34.4%  56.6%^e^ | 1-year aHR  2.0 (1.5-2.6)  1.0 (ref) | Birth year, presence of hydrocephalus at birth, presence of other CAs, gestational age, infant sex, type of lesion |  |
|  | |  | Birth year | 1976-78  1979-81  1982-84  1985-87 | 1-year survival  41.5%  37.9%  43.8%  59.4%^c^ | aHR  2.5 (1.7-3.8)^c^  2.5 (1.6-3.7)^c^  1.6 (1.1-2.6)^c^  1.0 (ref) | Presence of hydrocephalus at birth, presence of other CAs, gestational age, infant sex, type of lesion |  |
|  | |  | Presence of other CAs | Syndrome/pos-  sible syndrome  Multiple  Isolated NTD | 1-year survival  5.6%  14.0%^e^  47.9% | aHR  3.9 (2.3-6.8)^e^  2.7 (1.9-3.9)^c^  1.0 (ref) | Presence of hydrocephalus at birth, gestational age, infant sex, type of lesion, birth year |  |
|  | |  | Gestational age (weeks) ^a^ | <37  ≥37 | 1-year survival  16.4%^e^  47.0% | aHR  2.8 (2.0-3.9)^e^  1.0 (ref) | Presence of hydrocephalus at birth, presence of other CAs, infant sex, type of lesion, birth year |  |
|  | |  | Type of lesion^a^ | Spina bifida n.o.s.  Encephalocele  Cranial meningocele  Spinal myelo-meningocele  Spinal meningocele | 1-year survival  25.5%^e^  32.9%^e^  75%  43.1%^e^  84.7% | aHR  7.1 (3.7–13.7^e^  5.9 (3.0–11.5)^e^  2.1 (0.3-16.1)^g^  3.3 (1.8-6.2)^e^  1.0 (ref) | Presence of hydrocephalus at birth, presence of other CAs, gestational age, infant sex, birth year |  |
| **Spina bifida** | |  |  |  |  |  |  |  |
| Myelomeningocele | | Borgstedt-Bakke, 2017 [7] | Birth period | Time trend 1990-2015 vs 1970-1979 and 1980-1989 | 25-year HR  0.7 (0.5-1.0), *p*=0.05 | **―** | **―** |  |
|  | |  | Child’s sex | Males vs females | 0.9 (0.4-1.9), *p*=0.73 | **―** | **―** |  |
|  | |  | Lesion level | Thoracic lesion vs lumbar | 3.4 (1.4-8.5), *p*=0.01 | **―** | **―** |  |
|  | | Shin, 2012 [8], 1997-2003^a^ | Maternal race/ethnicity | White  Black  Hispanic | 1-year survival  94.1 (92.6-95.4)  87.8 (82.5-91.6)^c^  92.2 (90.3-93.8) | 8-year aHR^a^ (for 1500-2499g)  1.0 (ref)  2.0 (0.7-5.8)  3.7 (1.8-7.8)^c^ | Presence of major CHD, birth cohort |  |
|  | |  | Presence of major CHD | No  Yes | 93.8 (92.6-94.7)^f^ 81.9 (75.4-86.8) | aHR^a^  1.0 (ref)  2.6 (1.3-5.0)^c^ (for 1500-2499g)  3.6 (2.1-6.1)^c^ (for ≥2500g) | Race/ethnicity, birth cohort |  |
|  | | Wang, 2015 [9] | Maternal ethnicity | NHW  NHB  Hispanic  A/PI  AI/AN | **―** | 8-year aHR  1.0 (ref)  1.3^g^  1.0^g^  0.2^g^  0.7^g^ | Birth weight and gestational age, maternal age, birth period, and state surveillance program |  |
|  | Wong, 2001 [10] ^a^ | | Birth weight (g) | <1500  1500-2499  ≥2500  <2500  ≥2500 | Survival to <18 years  (%, 95% CI)  33.3 (15-74)  68.2 (53-88)  82.8 (77-90)  **―** | 18-year aHR  2.3 (1.1-4.9)^c^  1.0 (ref) | Maternal ethnicity, location of the lesion, presence of multiple defects |  |
|  | |  | Level of lesion | Cervical  Thoracic  Lumbar  Sacral  High (cervical/thoracic)  Low (lumbar/sacral) | 87.5 (66, 100)  64.4 (50-83)  83.8 (72-97)  85.2 (74-98)  ― | aHR  3.4 (1.6-7.1)^c^  1.0 (ref) | Maternal ethnicity, birth weight, presence of multiple defects |  |
|  | |  | Maternal race/ethnicity | White  Black  Other | 82.8 (76-90)  67.1 (56-81)^c^  87.5 (63-100) | NS (Black vs White) - aHR not reported | Birth weight, presence of multiple defects, location of the lesion |  |
|  | |  | Multiple defects | Yes  No | 59.0 (42-84)  81.9 (76-88)^d^ | NS (Yes vs No) – aHR not reported | Maternal ethnicity, birth weight, location of the lesion |  |
| **Encephalocele** | |  |  |  |  |  |  |  |
|  | | Siffel, 2003 [11] | Presence of associated CAs | No  Yes | 20-year HR  1.0 (ref)  3.8 (1.7-8.6)^e^ | 20-year aHR  1.0 (ref)  2.8 (1.2-6.7)^c^ | Birth weight, race, birth cohort, gestational age |  |
|  | |  | Gestational age (weeks) | Preterm (<37)  Term (≥37) | 4.7 (2.1-10.5)^f^  1.0 (ref) | **―** |  |  |
|  | |  | Birth weight (g) | <2500  ≥2500 | 6.3 (2.7-14.4)^f^  1.0 (ref) | 5.2 (2.7-12.6)^f^  1.0 (ref) | Race, birth cohort, gestational age, presence of associated CAs |  |
|  | |  | Race | Black  Other | 2.7 (1.1-6.5)^c^  1.0 (ref) | 2.4 (0.95-5.9)^g^  1.0 (ref) | Birth weight, birth cohort, gestational age, presence of associated CAs |  |
|  | |  | Birth cohort | 1989-98  1979-88  1989-98 | 0.5 (0.2-1.2)^g^  1.0 (ref)  **―** | 0.4 (0.2-1.0)  1.0 (ref)  For <2500g only  0.3 (0.01-0.9)^c^ | Birth weight, race, gestational age, presence of associated CAs  Race, gestational age, presence of associated CAs |  |
|  | | Wang, 2015 [9]  Survival ≤8 years | Maternal ethnicity | NHW  NHB  Hispanic  A/PI  AI/AN | ― | 8-year aHR  1.0 (ref)  0.7^g^  1.3^g^  2.6^c^  2.8^c^ | Birth weight and gestational age, maternal age, birth period, and state surveillance program |  |
| **Orofacial clefts** | |  |  |  |  |  |  |  |
| Cleft palate only | | Wang, 2015 [9]  Survival ≤8 years | Maternal ethnicity | NHW  NHB  Hispanic  A/PI  AI/AN | ― | 8-year aHR  1.0 (ref)  1.4^c^  1.4^c^  1.1^g^  0.5^g^ | Birth weight and gestational age, maternal age, birth period, and state surveillance program |  |
| Cleft lip with or without cleft palate | | Wang, 2015 [9] | Maternal ethnicity | NHW  NHB  Hispanic  A/PI  AI/AN | ― | 8-year aHR  1.0 (ref)  1.3^c^  1.3^c^  0.6^c^  1.1^g^ | Birth weight and gestational age, maternal age, birth period, and state surveillance program |  |
| **Gastro-intestinal anomalies** | | |  |  |  |  |  |  |
| **Oesophageal atresia** | | |  |  |  |  |  |  |
|  | | Cassina, 2016 [12], 1981-2012 | Birth weight )g) | ≥2500  <2500 | ― | 25-year aHR  1.0 (ref)  3.7 (1.7-8.3)^d^ | Birth period, presence of additional anomalies |  |
|  | |  | Birth period | 1997+  Before 1997 | 10-year survival  (non-isolated only)  87.3 (81.2–93.4)^d^  58.7 (44.4-73.0) | 25-year aHR  1.0 (ref)  2.4 (1.3-4.8)^d^ | Birth weight, presence of additional anomalies |  |
|  | |  | Presence of additional CAs | Isolated  Non-isolated | 25-year survival  91.8 (86.9-96.7^c^  79.2 (72.9-85.5) | 1.0 (ref)  2.8 (1.3-6.0)^d^ | Birth period, birth weight |  |
|  | | Oddsberg, 2012 [13] | Presence of associated CAs | Any CA  Circulatory CA  Non-circulatory CA  None | 40-year HR  4.7 (3.5–6.3)  5.4 (3.9–7.5)  4.2 (3.0–5.8)  1.0 (ref) | 40-year aHR  4.9 (3.7–6.6)  5.6 (4.0–7.8)  4.5 (3.2–6.2)  1.0 (ref) | Gender, birth weight, birth year period |  |
|  | |  | Birth weight (g) | <1500  ≥1500 | ― | 7.0 (4.9-10.1)  1.0 (ref) | Gender, associated anomalies, birth year period |  |
|  | |  | Birth year period | 1964-69  1970-79  1980-89  1990-99  2000-2007 | ― | 4.6 (2.3–9.2)  3.1 (2.0–4.7)  2.1 (1.4–3.2)  1.2 (0.8–1.8)  1.0 (ref) | Gender, associated anomalies, birth weight |  |
|  | | Wang, 2015 [9] | Maternal ethnicity | NHW  NHB  Hispanic  A/PI  AI/AN | ― | 8-year aHR  1.0 (ref)  2.0^c^  1.4^c^  1.3^g^  1.2^g^ | Birth weight and gestational age, maternal age, birth period, and state surveillance program |  |
| **Anorectal atresia or stenosis** | | |  |  |  |  |  |  |
| Anorectal malformations | | Cassina, 2019 [14] | Presence of two or more associated anomalies | Yes  No | HR  7.9 (2.2-27.8)^d^  1.0 (ref) | ― | ― |  |
|  | |  | Birth weight (g) | <2500g  ≥2500 | 6.4 (2.3-17.9)^e^  1.0 (ref) | ― | ― |  |
|  | |  | Birth year period | 1990-1999  2000-2012 | 4.7 (1.8-11.8)^d^  1.0 (ref) | ― | ― |  |
| Anorectal atresia or stenosis | | Wang, 2015 [9] | Maternal ethnicity | NHW  NHB  Hispanic  A/PI  AI/AN | ― | 8-year aHR  1.0 (ref)  1.4^c^  1.4^c^  1.2^g^  1.5^g^ | Birth weight and gestational age, maternal age, birth period, and state surveillance program |  |
| **Hirschsprung disease** | | |  |  |  |  |  |  |
|  | | Löf Granström, 2017 [15] | Birth period | 1964-1980  1981-2000  2001-2013 | OR 1.0 (ref)  0.6 (0.1-4.2)^g^  0.4 (0.1-3.3)^g^ | ― | ― |  |
| **Biliary atresia** | |  |  |  |  |  |  |  |
|  | | Chardot, 2013 [16]  For 20-year NLS | Anatomical type (French classification) | Type 1  Type 2  Type 3  Type 4 | 20-year survival  82.5% (SE=8.0%)^f^  51.9% (SE=7.2%)  37.2% (SE=4.3%)  23.5% (SE=2.5%) | RR (20-year NLS)  0.13 (0.04-0.40)^f^  0.47 (0.32-0.71)  0.69 (0.54-0.87)  1.0 (ref) | Presence of BASM, age at Kasai operation |  |
|  | |  | BASM | Absent  Present | 31.2% (SE=2.3%)^f^  15.1% (SE=4.6%) | 0.59 (0.45-0.78)^e^  1.0 (ref) | Anatomical type, age at Kasai operation |  |
|  | |  | Age at KP (days) | ≤30 days  31-60 days  61-90 days  >90 days | 38.9% ((SE=7.5%)^d^  31.7% (SE=3.4%)  28.1% (SE=3.1%)  18.7% (SE=4.8%) | 0.54 (0.37-0.79)^f^  0.58 (0.45-0.75)  0.74 (0.37-0.79)  1.0 (ref) | Anatomical type, presence of BASM |  |
|  | | 5-year survival | Centre caseload^h^ – 1986-1996^c^ | ≥20  3 to5  ≤2 | 5-year survival (95% CI)  77.6 (72.1-83.1)  61.9 (51.1-72.7)  69.6 (62.5-76.7) | **―** | **―** |  |
|  | |  | Centre caseload^h^ – 1997-2002^g^ | ≥20  3 to5  ≤2 | 88.2 (82.5-93.9)  91.2 (84.5-97.9)  84.7 (76.7-92.7) | **―** | **―** |  |
|  | |  | Centre caseload^h^ – 2003-2009 ^g^ | ≥20  3 to5  ≤2 | 92.0 (87.7-96.3)  86.2 (78.4-94.0)  84.8 (76.8-92.8) | **―** | **―** |  |
|  | | Davenport, 2011 [17] - NLS | Age at KP (days) for isolated BA | <44 days  44-55  56-69  70+ | No significant difference in 10-yr NLS  χ^2^ =3.3, *p*=0.34 or between two most different (<44 and 44-55) groups: χ^2^ =2.1, *p*=0.15 (Fisher's Exact test.) | | **―** |  |
|  | | De Carvalho, 2010 [18] | Age at KP (days) | ≤60 days  61-90  >90 | 4-year NLS HR 1.0 (ref)  1.6 (1.2-2.3)^d^  1.9 (1.3-2.7)^d^ | **―** | **―** |  |
|  | | De Vries, 2011 [19] | Age at KP (days) (log-rank-test) | <45 days  45-60  60-75  >75 | 20-year NLS survival  14±9%^g^ (vs 45-60 or 60-75 days)  33±8%^g^ (vs 60-75)  42±10%^c^ (vs >75)  11±6% | **―** | **―** |  |
|  | | Leonhardt, 2011 [20] | Centre caseload | <5  ≥5 | 2-yr NLS  7.7%  26.4%^d^ | ― | **―** |  |
|  | |  | Centre caseload | <5  ≥5 | 2-yr overall survival after KP  73.5%  83.7%^g^ | **―** | **―** |  |
|  | | McKiernan, 2000 [21] | Centre caseload – overall survival^a^ | annual number of cases  <5  >5 | 5-year RR  1.0 (ref)  0.32 (0.11-0.94) | The only significant factor, RR not reported | Age at surgery, sex, gestational age, presence of BASM |  |
|  | |  | Centre caseload - NLS^a^ | <5  >5 | RR (5-year NLS)  1.0 (ref)  0.48 (0.28-0.86) | The only significant factor, RR not reported | Age at surgery, sex, gestational age, presence of BASM |  |
|  | | McKiernan, 2009 [22] | Centre caseload (annual number of cases) ^a^ | <5  >5 | Overall 13-year survival (95% CI)  75% (61.6–89.4)  89.5% (81.3–97.7) – *p*=0.052 | ― | ― |  |
|  | |  | Centre caseload (annual number of cases) ^a^ | <5  >5 | 13-year NLS (%)  27.3 (12.3-42.3)  54.0 (40.8-67.2)^d^ | ― | ― |  |
|  | | Pakarinen, 2018 [23] (5-year NLS) | Gender ^a^ | Female  Male | 5-year NLS  51 (39-63)^g^  60 (48-72) | 5-year aHR  0.5 (0.3-0.9)^c^  1.0 (ref) | Presence of associated CAs, age at KP; anatomical type of BA, presence of BASM, clearance of jaundice, European ethnicity, centre caseload |  |
|  | |  | Age at KP (days)^a^ | < 65  >65 | 66 (55-78)^d^  44 (32-56) | 1.5 (0.8-2.9)^g^  1.0 (ref) | Presence of associated CAs; gender; anatomical type of BA, presence of BASM, clearance of jaundice, European ethnicity, centre caseload |  |
|  | |  | Centre annual caseload^a^ | >3  <3 | 66 (54-77)^d^  44 (32-56) | 3.5 (1.8-6.8)^e^  1.0 (ref) | Presence of associated CAs, age at surgery, gender, anatomical type of BA, presence of BASM, clearance of jaundice, European ethnicity |  |
|  | |  | Clearance of jaundice after KP^a^ | <20 μmol/l  ≥20 μmol/l | 86 (78-94)^f^  4.3 (0-10) | 32.0 (14.8-69.2)^f^  1.0 (ref) | Presence of associated CAs, age at surgery, gender, anatomical type of BA, presence of BASM, centre caseload, European ethnicity |  |
|  | | Schreiber, 2007 [24] | Age at KP (days)^a^ | ≤30  31-90  >90 | 4-year NLS (%, 95% CI)  49 (26-69)^f^  36 (28-43)^f^  23 (12-37) ^f^ | ― | ― |  |
|  | | Wildhaber, 2008 [25] | Age at KP (days)^a^ | ≤45  46-75  >75 | 4-year NLS (%, ±SE)  75% ±15.3%  33.3% ± 10.3%  11.3% ± 10.6% | ― | ― |  |
| **Congenital diaphragmatic hernia (CDH)** | | |  |  |  |  |  |  |
|  | | Hinton, 2017 [26] | Treatment era^i^ | <1988  ≥1988 | 20-year HR  1.94 (1.25–3.27)  1.0 (ref) | 20-year aHR  2.11 (1.25–3.57)  1.0 (ref) | Neighbourhood poverty, presence of additional CAs |  |
|  | |  | Neighbourhood poverty | ≥10%  <10% | 1.83 (1.11–3.00)  1.0 (ref) | 1.72 (1.04–2.84)  1.0 (ref) | Treatment era, presence of additional CAs |  |
|  | |  | Presence of additional CAs | Other CAs  Isolated | 2.08 (1.24–3.48)  1.0 (ref) | 2.06 (1.22–3.49)  1.0 (ref) | Treatment era, neighbourhood poverty |  |
|  | | Wang, 2015 [9] | Maternal ethnicity | NHW  NHB  Hispanic  A/PI  AI/AN | ― | 8-year aHR  1.0 (ref)  1.4^c^  0.9^g^  0.8^g^  0.8^g^ | Birth weight and gestational age, maternal age, birth period, and state surveillance program |  |
| **Abdominal wall defects** | | |  |  |  |  |  |  |
| **Gastroschisis** | | Wang, 2015 [9] | Maternal ethnicity | NHW  NHB  Hispanic  A/PI  AI/AN | ― | 8-year aHR  1.0 (ref)  1.2^g^  1.1^g^  1.2^g^  1.7^g^ | Birth weight and gestational age, maternal age, birth period, and state surveillance program |  |
| **Omphalocele** | | Wang, 2015 [9] | Maternal ethnicity | NHW  NHB  Hispanic  A/PI  AI/AN | ― | 8-year aHR  1.0 (ref)  0.9^g^  1.1^g^  0.7^g^  0.8^g^ | Birth weight and gestational age, maternal age, birth period, and state surveillance program |  |
| **Down syndrome** | |  |  |  |  |  |  |  |
|  | | Brodwall, 2018 [27] - for 5-year survival | Presence of ECM or CHD^a^ | Down syndrome (no additional CAs)  ECM  AVSD, no ECM  AVSD + ECM  Conotruncal,^b^ no ECM  Conotruncal +  ECM  Other CHD, no ECM  Other CHD + ECM | ― | 5-year aHR  1.0 (ref)  2.6 (0.6-12)  4.2 (2.0-8.7)  13 (3.9-40)  7.4 (2.6–21)  28 (8.9-88)  4.3 (1.4-13)  19 (5.2-67) | Year of birth |  |
|  | | Chua, 2020 [28] | Birth weight | <2500  ≥2500 | n/a | 5-year aHR  1.9 (1.2 -3.0)^c^  1.0 (ref) | Age and sex |  |
|  | |  | Presence of CHD | Yes  No | n/a | 5-year aHR  1.9 (1.2 -3.0)^c^  1.0 (ref) | Age and sex |  |
|  | |  | Birth cohort | 1995-1999  2000-2004  2005-2009  2010-2014 | n/a | 5-year aHR  1.0 (ref)  0.4 (0.2-0.8)^c^  0.5 (0.3-1.0)^c^  0.5 (0.3-1.0)^g^ | Age and sex |  |
|  | | Glasson, 2016 [29] | Gestational age (weeks) | <37  ≥37 | 25-year HR  2.4 (1.5-3.7^e^  1.0 (ref) | 25-year aHR  1.9 (1.1-3.3)^c^  1.0 (ref) | Sex, birth cohort, aboriginality, presence of a CHD |  |
|  | |  | Birth weight | <2500  ≥2500 | 2.3 (1.4-3.7)^e^  1.0 (ref) | 1.8 (1.0-3.1)^c^  1.0 (ref) | Sex, birth cohort, aboriginality, presence of a CHD |  |
|  | |  | Aboriginality | Aboriginal  Non-aboriginal | 1.6 (0.7-3.8)^g^  1.0 (ref) | 1.1 (0.5-2.7)^g^  1.0 (ref) | Sex, birth cohort, presence of a CHD |  |
|  | |  | Birth cohort | 1980-1990  1991-2000  2001-2010 | 2.9 (1.7-5.2)^e^  0.9 (0.5-1.9)^g^  1.0 (ref) | 2.9 (1.6-5.2)^e^  0.7 (0.4-1.5)^g^  1.0 (ref) | Sex, aboriginality, presence of a CVD |  |
|  | |  | Presence of CHD | Yes  No | 2.9 (1.7-4.9)^e^  1.0 (ref) | 3.1 (1.8-5.3)^e^  1.0 (ref) | Sex, aboriginality, birth cohort |  |
|  | |  | Sex | Male  Female | 1.1 (0.7-1.7)^g^  1.0 (ref) | 1.2 (0.8-1.9)^g^  1.0 (ref) | Birth cohort, aboriginality, presence of a CHD |  |
|  | | Hayes, 1997 [30] | Presence of CAVD | No  Yes | 10-yr survival  90%  58% | RR  1.0 (ref)  5.6 (3.2-9.7)^e^ | Leukaemia (only significant variables in the bivariate model, i.e CAVD and leukaemia were included in the in the Cox proportional hazards model) |  |
|  | |  | Presence of leukaemia |  | Not reported | 11.2 (3.9-32.2)^e^ | CAVD |  |
|  | | Kucik, 2013 [31]) | Race/ethnicity | White  Black  Hispanic  Other | **―** | 20-year aHR  1.0 (ref)  1.4 (1.0–1.6)  0.8 (0.7–0.9)^c^  1.3 (1.1–1.6)^c^ | Birth weight, maternal age and education, presence of a CHD, birth period, and region of birth. |  |
|  | |  | Birth weight | <1500  1500-2499  ≥2500 | **―** | 8.5 (7.3–9.8)^c^  1.8 (1.6–2.0)^c^  1.0 (ref) | Race/ethnicity, maternal age and education, presence of a CHD, birth period, and region of birth |  |
|  | |  | Presence of CHD | Yes  No | **―** | 2.7 (2.4–3.0)^c^  1.0 (ref) | Race/ethnicity, birth weight, maternal age and education, birth period, and region of birth |  |
|  | |  | Birth cohort | 1983-1989  1990-1996  1997-2003 | **―** | 1-5-year aHR  1.0 (ref)  0.6 (0.5–0.8)^c^  0.5 (0.4–0.7)^c^ | Race/ethnicity, birth weight, maternal age and education, presence of a CHD and region of birth |  |
|  | | Leonard, 2000 [32] | Birth cohort | 1991-96  1983-89 | 10-year HR  0.4 (0.2-0.8)^d^  1.0 (ref) | 10-year aHR  0.3 (0.2-0.7)^d^  1.0 (ref) | Aboriginality, birth weight, presence of CHD, maternal age group, gender |  |
|  | |  | Birth weight (g) | <2500  ≥2500 | 2.3 (1.4-4.0)^d^  1.0 (ref) | 2.2 (1.2-3.7)^d^  1.0 (ref) | Aboriginality, presence of CHD, maternal age group, sex, birth cohort |  |
|  | |  | Aboriginality | Yes  No | 3.2 (1.4-7.4)^d^  1.0 (ref) | 3.2 (1.3-7.9)^d^  1.0 (ref) | Presence of CHD, birth weight, maternal age, sex, birth cohort |  |
|  | |  | Presence of CHD | Yes  No | 3.4 (2.0-5.9)  1.0 (ref) | 3.7 (2.1-6.7)^d^  1.0 (ref) | Aboriginality, birth weight, maternal age, sex, birth cohort |  |
|  | |  | Maternal age (years) | <20  ≥20 | 2.8 (1.1-7.1)^c^  1.0 (ref) | 2.4 (0.9-6.1)^g^  1.0 (ref) | Aboriginality, presence of CHD, sex, birth cohort, birth weight |  |
|  | |  | Sex | Female  Male | 1.9 (1.1-3.2)^c^  1.0 (ref) | 1.5 (0.9-2.5)^g^  1.0 (ref) | Aboriginality, presence of CHD, maternal age, birth cohort, birth weight |  |
|  | | Rankin, 2012 [33]  1985-2003^a^ | Birth year | Continuous | HR  0.93 (0.89–0.96)^e^ | 20-year aHR  0.89 (0.85–0.92)^e^ | Presence of additional structural anomalies, gestational age, maternal age, birth weight, karyotype, IMD, plurality, infant gender |  |
|  | |  | Gestational age | Continuous | 0.80 (0.76–0.84)^e^ | 0.76 (0.72–0.80)^e^ | Presence of additional structural anomalies, birth year, maternal age, birth weight, karyotype, IMD, plurality, infant gender |  |
|  | |  | Birth weight, z score | Continuous | 0.88 (0.77–1.0) | 0.81 (0.71–0.91) | Presence of additional structural anomalies, birth year, maternal age, gestational age, birth year, karyotype, IMD, plurality, infant gender |  |
|  | |  | Karyotype^c^ | Trisomy 21  Mosaic  Translocation | 1.0 (ref)  Inestimable  0.74 (0.23–2.32) | 1.0 (ref)  Inestimable (no deaths)  0.97 (0.30–3.11) | Presence of additional structural anomalies, birth year, maternal age, gestational age, birth year, IMD, plurality, infant gender, birth weight |  |
|  | |  | Additional structural CAs^e^ | None  CHD only  Digestive only  CHD and digestive only  Other(s) | 1.0 (ref)  3.8 (2.4-6.0)^e^  5.1 (2.1-12.4)  8.8 (3.3-18.0)^e^  3.5 (1.2-10.0)^c^ | 1.0 (ref)  5.0 (3.1-8.1)^e^  6.5 (2.6 (16.1)^e^  7.8 (3.8-16.4)^e^  5.1 (1.7-15.1)^d^ | Birth year, maternal age, gestational age, IMD, karyotype, plurality, infant gender, birth weight |  |
|  | | Schneuer, 2019 [4] | Presence of CHD | None  CHD | 5-year survival  93.7 ( 90.5–96.9)  92.0 ( 88.3–95.8)^g^ | n/a | n/a |  |
|  | | Wang, 2015 [9] | Maternal ethnicity | NHW  NHB  Hispanic  A/PI  AI/AN | **―** | 8-year aHR  1.0 (ref)  1.4^c^  1.0^g^  1.1^g^  1.4^g^ | Birth weight and gestational age, maternal age, birth period, and state surveillance program |  |
| **Trisomy 13** | | |  |  |  |  |  |  |
|  | | Meyer, 2016 [34]  (significant factors only included) | Gestational age (weeks) | <32  32-36  ≥37 | 1-yr survival probability  6.6 (3.1-11.9)^e^  8.1 (5.0-12.1)  15.2 (11.6-19.2) | 5-year aHR  1.9 (1.5-2.5)^c^  1.3 (1.0-1.6)  1.0 (ref) | Sex, maternal ethnicity, State, geographical area |  |
|  | |  | Participating State | Massachusetts  Other individual states  Texas | 10.8 (3.4-23.0)^g^  Ranging between 8.0 and 22  9.8 (6.5-13.8) | 1.7 (1.1-2.6)^c^  Not significant  1.0 (ref) | Sex, maternal ethnicity, geographical area |  |
| **Trisomy 18** | | |  |  |  |  |  |  |
|  | | Meyer, 2016 [34]^a^ | Gestational age (weeks) | <32  32-36  ≥37 | 1-yr survival  4.9 (2.5-8.4)^e^  9.4 (6.3-13.2)  17.2 (14.3-20.3) | 5-year aHR  2.7 (2.2-3.4)^c^  1.5 (1.2-1.8)^c^  1.0 (ref) | Sex, maternal ethnicity, plurality, presence of CHD, presence of omphalocele, State, geographical area |  |
|  | |  | Sex | Female  Male | 14.4 (11.9-17.1)^e^  10.8 (8.1-13.9) | 0.7 (0.6-0.9)^c^  1.0 (ref) | Gestational age, maternal ethnicity, plurality, presence of CHD, presence of omphalocele, State, geographical area |  |
|  | |  | Presence of CHD | Yes  No | 5.7 (3.0-9.6)^g^  15.0 (12.8-17.4) | 1.3 (1.1-1.6)^c^  1.0 (ref) | Gestational age, maternal ethnicity, plurality, sex, presence of omphalocele, State, geographical area |  |
|  | |  | Presence of omphalocele | Yes  No | 3.2 (1.4-13.0)^c^  13.8 (11.8-16.0) | 1.6 (1.1-2.3)^c^  1.0 (ref) | Gestational age, maternal ethnicity, plurality, sex, presence of CHD, State, geographical area |  |
|  | |  | Maternal ethnicity | NH White  NH Black  Hispanic  NH Asian/PI  Other/unknown | 13.6 (10.7-16.9)  17.3 (12.5-22.7)^c^  10.1 (7.3-13.5)  13.2 (4.8-25.8)  23.3 (10.3-39.4) | 1.0 (ref)  0.7 (0.6-0.9)^c^  0.9 (0.8-1.1)  0.8 (0.5-1.2)  1.0 (0.6-1.7) | Gestational age, plurality, sex, presence of CHD, presence of omphalocele, State, geographical area |  |
| **Prader-Willi syndrome** | | |  |  |  |  |  |  |
|  | | Lionti, 2012 [35] | Presence of obesity (BMI ≥30kg/m^2^ for >18 year-olds and sex- and age specific BMI centiles for 2-18 year-olds) | Yes  No | 25-year survival  85%^c^  100% | **―** | **―** |  |

**Note:**

^a^Only predictors with significant results in either unadjusted or adjusted analysis are shown.

^b^Conotruncal defects include Tetralogy of Fallot, double outlet right ventricle, conotruncal ventricular septal defects, aortic hypoplasia, truncus arteriosus and interrupted aortic arch;

^c^p<0.05 (also for those significant associations where the exact p value not reported), ^d^p<0.01, ^e^p<0.001, ^f^p<0.0001, ^g^not significant (p≥0.05)

^h^centre where the child was primarily treated

^i^Treatment eras are before 1988 (routine immediate surgical repair), and post-1988 (preoperative stabilization, delayed surgical repair, and addition of lung-sparing strategies).

AGA, appropriate for gestational age; aHR, adjusted Hazard Ratio; A/PI, Asian/Pacific Islander; AI/AN, American Indian/Alaska Native; AVSD, atrioventricular septal defect, BASM=biliary atresia splenic malformation syndrome; CAVD, complete atrio-ventricular defect; CHD, Congenital heart disease; ECM, extracardiac malformations; HR, Hazard Ratio; IMD, Index of Multiple Deprivation; KP, Kasai hepatoportoenterostomy; LGA, large for gestational age; NH, Non-Hispanic; NHB, Non-Hispanic Black; NHW, Non-Hispanic White; NLS, native liver survival; NS, not significant (results not reported); OR, odds ratio; RR, relative risk; SGA, small for gestational age.

**References**

1. Agha MM, Williams JI, Marrett L, To T, Dodds L. Determinants of survival in children with congenital abnormalities: a long-term population-based cohort study. Birth Defects Res A Clin Mol Teratol. 2006;76(1):46-54. PMID: 16397887.

2. Berger KH, Zhu BP, Copeland G. Mortality throughout early childhood for Michigan children born with congenital anomalies, 1992-1998. Birth Defects Res A Clin Mol Teratol. 2003;67(9):656-61. PMID: 14703790.

3. Nembhard WN, Salemi JL, Ethen MK, Fixler DE, Canfield MA. Mortality among infants with birth defects: Joint effects of size at birth, gestational age, and maternal race/ethnicity. Birth Defects Res A Clin Mol Teratol. 2010;88(9):728-36. doi: <https://dx.doi.org/10.1002/bdra.20696>. PMID: 20672351.

4. Schneuer FJ, Bell JC, Shand AW, Walker K, Badawi N, Nassar N. Five-year survival of infants with major congenital anomalies: a registry based study. Acta Paediatr Int J Paediatr. 2019;108(11):2008-18. doi: <http://dx.doi.org/10.1111/apa.14833>. PMID: 628179265.

5. Wang Y, Hu J, Druschel CM, Kirby RS. Twenty-five-year survival of children with birth defects in New York State: a population-based study. Birth Defects Res A Clin Mol Teratol. 2011;91(12):995-1003. doi: <https://dx.doi.org/10.1002/bdra.22858>. PMID: 21960515.

6. Sutton M, Daly LE, Kirke PN. Survival and disability in a cohort of neural tube defect births in Dublin, Ireland. Birth Defects Res A Clin Mol Teratol. 2008;82(10):701-9. doi: 10.1002/bdra.20498. PMID: 18803309.

7. Borgstedt-Bakke JH, Fenger-Gron M, Rasmussen MM. Correlation of mortality with lesion level in patients with myelomeningocele: a population-based study. J Neurosurg Pediatrics. 2017;19(2):227-31. doi: <https://dx.doi.org/10.3171/2016.8.PEDS1654>. PMID: 27911247.

8. Shin M, Kucik JE, Siffel C, Lu C, Shaw GM, Canfield MA, et al. Improved survival among children with spina bifida in the United States. J Pediatr. 2012;161(6):1132-7. doi: 10.1016/j.jpeds.2012.05.040. PMID: 22727874.

9. Wang Y, Liu G, Canfield MA, Mai CT, Gilboa SM, Meyer RE, et al. Racial/ethnic differences in survival of United States children with birth defects: A population-based study. J Pediatr. 2015;166(4):819-26.e2. doi: <http://dx.doi.org/10.1016/j.jpeds.2014.12.025>. PMID: 601970400.

10. Wong LY, Paulozzi LJ. Survival of infants with spina bifida: a population study, 1979-94. Paediatr Perinat Epidemiol. 2001;15(4):374-8. PMID: 11703686.

11. Siffel C, Wong LY, Olney RS, Correa A. Survival of infants diagnosed with encephalocele in Atlanta, 1979-98. Paediatr Perinat Epidemiol. 2003;17(1):40-8. PMID: 12562471.

12. Cassina M, Ruol M, Pertile R, Midrio P, Piffer S, Vicenzi V, et al. Prevalence, characteristics, and survival of children with esophageal atresia: A 32-year population-based study including 1,417,724 consecutive newborns. Birth Defects Res A Clin Mol Teratol. 2016;106(7):542-8. doi: <https://dx.doi.org/10.1002/bdra.23493>. PMID: 26931365.

13. Oddsberg J, Lu Y, Lagergren J. Aspects of esophageal atresia in a population-based setting: incidence, mortality, and cancer risk. Pediatr Surg Int. 2012;28(3):249-57. doi: <https://dx.doi.org/10.1007/s00383-011-3014-1>. PMID: 22020495.

14. Cassina M, Fascetti Leon F, Ruol M, Chiarenza SF, Scire G, Midrio P, et al. Prevalence and survival of patients with anorectal malformations: A population-based study. J Pediatr Surg. 2019;54(10):1998-2003. doi: <https://dx.doi.org/10.1016/j.jpedsurg.2019.03.004>. PMID: 30935729.

15. Löf Granström A, Wester T. Mortality in Swedish patients with Hirschsprung disease. Pediatr Surg Int. 2017;33(11):1177-81. doi: <http://dx.doi.org/10.1007/s00383-017-4150-z>. PMID: 618224664.

16. Chardot C, Buet C, Serinet MO, Golmard JL, Lachaux A, Roquelaure B, et al. Improving outcomes of biliary atresia: French national series 1986-2009. J Hepatol. 2013;58(6):1209-17. doi: 10.1016/j.jhep.2013.01.040. PMID: 23402746.

17. Davenport M, Ong E, Sharif K, Alizai N, McClean P, Hadzic N, et al. Biliary atresia in England and Wales: results of centralization and new benchmark. J Pediatr Surg. 2011;46(9):1689-94. doi: 10.1016/j.jpedsurg.2011.04.013. PMID: 21929975.

18. De Carvalho E, Santos JL, Silveira TR, Kieling CO, Silva LR, Porta G, et al. Biliary atresia: the Brazilian experience. J Pediatr (Rio J). 2010;86(6):473-9. doi: 10.2223/JPED.2054. PMID: 21140036.

19. de Vries W, Homan-Van der Veen J, Hulscher JB, Hoekstra-Weebers JE, Houwen RH, Verkade HJ, et al. Twenty-year transplant-free survival rate among patients with biliary atresia. Clin Gastroenterol Hepatol. 2011;9(12):1086-91. doi: 10.1016/j.cgh.2011.07.024. PMID: 21820397.

20. Leonhardt J, Kuebler JF, Leute PJ, Turowski C, Becker T, Pfister ED, et al. Biliary atresia: lessons learned from the voluntary German registry. Eur J Pediatr Surg. 2011;21(2):82-7. doi: <https://dx.doi.org/10.1055/s-0030-1268476>. PMID: 21157692.

21. McKiernan PJ, Baker AJ, Kelly DA. The frequency and outcome of biliary atresia in the UK and Ireland. Lancet. 2000;355(9197):25-9. doi: 10.1016/S0140-6736(99)03492-3. PMID: 10615887.

22. McKiernan PJ, Baker AJ, Lloyd C, Mieli-Vergani G, Kelly DA. British paediatric surveillance unit study of biliary atresia: outcome at 13 years. J Pediatr Gastroenterol Nutr. 2009;48(1):78-81. doi: 10.1097/MPG.0b013e31817d80de. PMID: 19172128.

23. Pakarinen MP, Johansen LS, Svensson JF, Bjornland K, Gatzinsky V, Stenstrom P, et al. Outcomes of biliary atresia in the Nordic countries - a multicenter study of 158 patients during 2005-2016. J Pediatr Surg. 2018;53(8):1509-15. doi: 10.1016/j.jpedsurg.2017.08.048. PMID: 28947328.

24. Schreiber RA, Barker CC, Roberts EA, Martin SR, Alvarez F, Smith L, et al. Biliary atresia: the Canadian experience. J Pediatr. 2007;151(6):659-65, 65 e1. doi: 10.1016/j.jpeds.2007.05.051. PMID: 18035148.

25. Wildhaber BE, Majno P, Mayr J, Zachariou Z, Hohlfeld J, Schwoebel M, et al. Biliary atresia: Swiss national study, 1994-2004. J Pediatr Gastroenterol Nutr. 2008;46(3):299-307. doi: 10.1097/MPG.0b013e3181633562. PMID: 18376248.

26. Hinton CF, Siffel C, Correa A, Shapira SK. Survival Disparities Associated with Congenital Diaphragmatic Hernia. Birth Defects Res A Clin Mol Teratol. 2017;109(11):816-23. doi: 10.1002/bdr2.1015. PMID: 28398654.

27. Brodwall K, Greve G, Leirgul E, Klungsøyr K, Holmstrøm H, Vollset SE, et al. The five-year survival of children with Down syndrome in Norway 1994–2009 differed by associated congenital heart defects and extracardiac malformations. Acta Paediatr Int J Paediatr. 2018;107(5):845-53. doi: 10.1111/apa.14223.

28. Chua GT, Tung KTS, Wong ICK, Lum TYS, Wong WHS, Chow CB, et al. Mortality Among Children with Down syndrome in Hong Kong: A Population-Based Cohort Study from Birth. J Pediatr. 2020;218:138-45. doi: <http://dx.doi.org/10.1016/j.jpeds.2019.11.006>. PMID: 2004554180.

29. Glasson EJ, Jacques A, Wong K, Bourke J, Leonard H. Improved Survival in Down Syndrome over the Last 60 Years and the Impact of Perinatal Factors in Recent Decades. J Pediatr. 2016;169:214-20.e1. doi: <https://dx.doi.org/10.1016/j.jpeds.2015.10.083>. PMID: 26651430.

30. Hayes C, Johnson Z, Thornton L, Fogarty J, Lyons R, O'Connor M, et al. Ten-year survival of Down syndrome births. Int J Epidemiol. 1997;26(4):822-9. PMID: 9279615.

31. Kucik JE, Shin M, Siffel C, Marengo L, Correa A. Trends in survival among children with down syndrome in 10 regions of the united states. Pediatrics. 2013;131(1):e27-e36. doi: <http://dx.doi.org/10.1542/peds.2012-1616>. PMID: 368184663.

32. Leonard S, Bower C, Petterson B, Leonard H. Survival of infants born with Down's syndrome: 1980-96. Paediatr Perinat Epidemiol. 2000;14(2):163-71. PMID: 10791661.

33. Rankin J, Tennant PW, Bythell M, Pearce MS. Predictors of survival in children born with Down syndrome: a registry-based study. Pediatrics. 2012;129(6):e1373-81. doi: <https://dx.doi.org/10.1542/peds.2011-3051>. PMID: 22614780.

34. Meyer RE, Liu G, Gilboa SM, Ethen MK, Aylsworth AS, Powell CM, et al. Survival of children with trisomy 13 and trisomy 18: A multi-state population-based study. Am J Med Genet A. 2016;170A(4):825-37. doi: <https://dx.doi.org/10.1002/ajmg.a.37495>. PMID: 26663415.

35. Lionti T, Reid SM, Rowell MM. Prader-Willi syndrome in Victoria: mortality and causes of death. J Paediatr Child Health. 2012;48(6):506-11. doi: <https://dx.doi.org/10.1111/j.1440-1754.2011.02225.x>. PMID: 22697408.
