# Supplementary material for: Long-term survival of children born with congenital anomalies: A systematic review and meta-analysis of population-based studies
Source: PLoS Med. 2020 Sep 28;17(9):e1003356. doi: 10.1371/journal.pmed.1003356 (PMC7521740; doi:10.1371/journal.pmed.1003356)
Supplement: S1 Text — PROSPERO, International Prospective Register of Systematic Reviews. (DOCX) [file pmed.1003356.s011.docx]

S2 Text. Protocol for PROSPERO registration

PROSPERO ID: CRD42017074675

# Review title and timescale

1. **Review title***

Long-term survival of children born with congenital anomalies: a systematic review of population-based studies

1. **Original language title**

See item 1.

1. **Anticipated or actual start date***

1^st^ November 2017

1. **Anticipated completion date***

31^st^ October 2018

1. **Stage of review at time of this submission***

The systematic review protocol has been finalised. Preliminary searches have been carried out.

**Review team details**

1. **Named contact***

Dr Svetlana Glinianaia

1. **Named contact email***

[svetlana.glinianaia@ncl.ac.uk](mailto:svetlana.glinianaia@ncl.ac.uk)

1. **Named contact address**

Institute of Health & Society, Newcastle University, Baddiley-Clark Building, Richardson Road, Newcastle upon Tyne, NE2 4AX, UK

1. **Named contact phone number**

+44 (0)191 208 5891

1. **Organisational affiliation of the review***

Institute of Health & Society, Newcastle University

<http://www.ncl.ac.uk/ihs/>

1. **Review team members and their organisational affiliation**

Dr Svetlana Glinianaia, Institute of Health & Society, Newcastle University

Dr Kate E Best, Institute of Health & Society, Newcastle University

Dr Rute Vieira, Institute of Health & Society, Newcastle University

Dr Michele Santoro, CNR (National Research Council) Institute of Clinical Physiology

Dr Alessio Coi, CNR (National Research Council) Institute of Clinical Physiology

Professor Judith Rankin, Institute of Health & Society, Newcastle University

1. **Funding sources/sponsors***

EU Horizon 2020 Research and Innovation Programme; Proposal number: 733001

1. **Conflicts of interest***

None

1. Collaborators

**Review methods**

1. **Review question(s)***

What is the long-term (beyond one year of age) survival of children with specific congenital anomalies?

What are the clinical and socio-demographic risk factors for survival of children with specific congenital anomalies?

1. **Searches***

In determining the search strategy, study selection and the presentation of the results, the MOOSE guidelines for Meta-Analyses and Systematic Reviews of Observational Studies will be followed.

We will search the following electronic bibliographical databases: MEDLINE, EMBASE, Scopus, PsycINFO, the Cochrane library and also the databases of the systematic reviews, i.e. PROSPERO, the JBI Database of Systematic Reviews and Implementation Reports.

The search strategy will consist of key words and MeSH headings (dependent on the database) combining the keywords for the population (birth, pregnancy, delivery), exposure (congenital anomaly, including specific anomaly groups) and outcome (long-term survival, mortality), incorporating elements of the PICO (Population/Patient Intervention Comparison Outcome) framework into our search strategy. We will search the reference lists of the included full papers and of relevant previous systematic reviews. We will search papers that have cited the included full papers (citation searching). We will also undertake keyword searches in key Journals (e.g. Birth Defects Research, Part A, Clinical and Molecular Teratology, Archives of Disease in Childhood, Pediatrics, The Journal of Pediatrics, Journal of Pediatric Surgery). Where there is not enough information in the study or where there is only a published abstract available, the study authors will be contacted for further information.

We will use the following limitations:

We will search for original population-based research studies in humans only;

time of publication will be restricted to the following period - 1^st^ January 1995 to 31 October 2017 - to focus on the survival during the last 30 years which greatly improved compared to 1960s-70s due to advances in prenatal diagnosis (resulting in a higher rate of terminations of pregnancy for a fetal anomaly, in particular for more severe or lethal conditions, thereby improving the survival of live births; in addition, prenatal diagnosis of conditions requiring surgery assured timely in-utero transfer to a tertiary centre and early surgery), neonatal care, including surgical interventions, ECMO etc;

for the initial searches, there will be no language limitations to provide the total number of publications in different languages (an abstract in English will be required) and to see if there are any relevant studies we could have missed; the final search will be restricted to publications in the English language.

1. **URL to search strategy**
2. **Condition or domain being studied***

Survival of children born with a major congenital anomaly

1. **Participants/population***

Individuals born with a major congenital anomaly, excluding congenital heart defects

1. **Intervention(s), exposure(s)***

Clinical (the use of anaesthesia, surgery, days spent in hospital, severity of the anomaly, additional anomalies) and sociodemographic (gender, maternal age, socioeconomic status) factors affecting survival

1. **Comparators(s)/control***

Background population of births, if this data is available in the study

# Inclusion criteria

- Population-based original research studies that report on long-term (beyond one year of life) survival (overall, pre-operative, post-operative, post discharge etc) of children born with a major congenital anomaly within a predefined geopolitical area.
- Studies will be included if they report survival estimates (or the number of patients born and the number or proportion alive at age > 1 years) on all congenital anomalies (overall survival) and by congenital anomaly group, and studies on selected congenital anomaly/ies

# Exclusion Criteria

- Studies not restricted to humans;
- Studies that were not written in the English language;
- Case studies and hospital-based studies, unless it is a specialised tertiary centre which covers a well-defined region;
- Studies that report survival during the first year of life only;
- Studies where patients were not followed from birth (e.g. follow up began after surgical correction);
- Population-based studies focused on children born with congenital heart disease (CHD) will be excluded because of the recently published systematic review on long-term survival of individuals born with CHD (Best and Rankin, Journal of the American Heart Association 2016;**5**(6):e002846)
- Studies exploring morbidity or quality of life when survival is not reported;
- Abstract only citations will be excluded, but where these are identified, the authors will be contacted for further information on the full paper;
- Reviews, editorials, position papers will be excluded, but the reference list will be scanned for relevant studies.

1. **Types of study to be included***

Population-based (register-based, retrospective and prospective cohort) studies

1. **Context**

Give summary details of the setting and other relevant characteristics which help define the inclusion or exclusion criteria

1. **Primary outcomes***

Long-term (beyond the first year of life) survival of children born with a major congenital anomaly

1. **Secondary outcomes***

No secondary outcomes

1. **Data extraction (selection and coding)**

Give the procedure for selecting studies for the review and extracting data, including the number of researchers involved and how discrepancies will be resolved. List the data to be extracted.

Using the search strategy, and the other sources described, titles and abstracts will be screened. A proportion of the titles and abstracts will be screened independently by a second reviewer. Full texts of potentially relevant citations will be retrieved and examined independently by two reviewers. If there are any disagreements between the reviewers, a discussion with a third reviewer will take place.

Information will be extracted from the included studies using a pre-piloted form. The form will include information on study details (study period, study location, type of a congenital anomaly etc.). Information will also be extracted on study methodology, case ascertainment and loss to follow-up. Odds ratios, hazard ratios, survival/mortality percentage will be extracted where applicable. Two reviewers will independently extract information from the included studies. Where there is not enough information in the study, the study authors will be contacted for further information.

1. **Risk of bias (quality assessment)***

State whether and how risk of bias will be assessed, how the quality of individual studies will be assessed, and whether and how this will influence the planned synthesis.

Only population-based studies will be included. In case of congenital anomaly register-based linkage studies, the percentage of successful linkage with birth and death certificates will be assessed. All studies will be included regardless of quality.

Quality will be assessed using the Newcastle-Ottawa scale for assessing the quality of non-randomised studies.

1. **Strategy for data synthesis***

Give the planned general approach to be used, for example whether the data to be used will be aggregate or at the level of individual participants, and whether a quantitative or narrative (descriptive) synthesis is planned. Where appropriate a brief outline of analytic approach should be given.

We plan to do a meta-analysis if, and where, sufficient data on specific groups of congenital anomalies are available.

1. **Analysis of subgroups or subsets***

Give any planned exploration of subgroups or subsets within the review. ‘None planned’ is a valid response if no subgroup analyses are planned.

We plan to analyse the survival of children with specific groups of congenital anomalies in addition to the overall survival.

Clinical and sociodemographic risk factors for survival will also be analysed.

Conditions detectable and not detectable by prenatal screening to analyse the effect of prenatal diagnosis on survival.

**General information**

1. **Type and method of review***

Select the type of review and the review method from the drop down list. Select the health area(s) of interest for your review. To select more than one hold down the Control key and click on your selections.

Meta-analysis, Systematic review

Child health

1. **Language**

Select the language(s) in which the review is being written and will be made available, from the drop down list. Use the control key to select more than one language

English

1. **Country**

Select the country in which the review is being carried out from the drop down list. For multi-national collaborations select all the countries involved. Use the control key to select more than one country.

England, Italy

1. **Other registration details**
2. **Reference and/or URL for published protocol**

Give the citation for the published protocol, if there is one.

Give the link to the published protocol, if there is one. This may be to an external site or to a protocol deposited with CRD in pdf format.

I give permission for this file to be made publicly available

NO

1. **Dissemination plans**

Give brief details of plans for communicating essential messages from the review to the appropriate audiences

Do you intend to publish the review on completion?

Yes

1. **Keywords**

Give words or phrases that best describe the review. (One word per box, create a new box for each term)

Long-term survival

Child mortality

Congenital anomaly

1. **Details of any existing review of the same topic by the same authors**

Give details of earlier versions of the systematic review if an update of an existing review is being registered, including full bibliographic reference if possible.

1. **Current review status***

Review status should be updated when the review is completed and when it is published.

Ongoing

1. **Any other information**

Provide any further information the review team consider relevant to the registration of the review

1. **Details of final report /publication(s)**

This field should be left empty until details of the completed review are available

***Appendix***

***NEWCASTLE - OTTAWA QUALITY ASSESSMENT SCALE COHORT STUDIES***

Note: A study can be awarded a maximum of one star for each numbered item within the Selection and Outcome categories. A maximum of two stars can be given for Comparability

# Selection

1. Representativeness of the exposed cohort

🞏 a) truly representative of the average (describe) in the community 🟑

🞏 b) somewhat representative of the average in the community 🟑

🞏 c) selected group of users eg nurses, volunteers d) no description of the derivation of the cohort

1. Selection of the non exposed cohort

🞏 a) drawn from the same community as the exposed cohort 🟑

🞏 b) drawn from a different source

🞏 c) no description of the derivation of the non exposed cohort

1. Ascertainment of exposure

🞏 a) secure record (eg surgical records) 🟑

🞏 b) structured interview 🟑

🞏 c) written self report

🞏 d) no description

1. Demonstration that outcome of interest was not present at start of study

🞏 a) yes 🟑

🞏 b) no

# Comparability

1) Comparability of cohorts on the basis of the design or analysis

🞏 a) study controls for (select the most important factor) 🟑

🞏 b) study controls for any additional factor 🟑 (This criteria could be modified to indicate specific control for a second important factor.)

# Outcome

1. Assessment of outcome

🞏 a) independent blind assessment 🟑

🞏 b) record linkage 🟑

🞏 c) self report

🞏 d) no description

1. Was follow-up long enough for outcomes to occur

🞏 a) yes (select an adequate follow up period for outcome of interest) 🟑

🞏 b) no

1. Adequacy of follow up of cohorts

🞏 a) complete follow up -all subjects accounted for 🟑

🞏 b) subjects lost to follow up unlikely to introduce bias -small number lost -> % (select an adequate %) follow up, or description provided of those lost) 🟑

🞏 c) follow up rate < % (select an adequate %) and no description of those lost

🞏 d) no statement

C-6
